# Unsaturated Phosphorus Electrophiles to Probe Protein Tyrosine Phosphatases

**DOI:** 10.1002/anie.202521902

**Published:** 2026-02-03

**Authors:** Eleftheria Poulou, Max Ruwolt, Christian E. Stieger, Kristin Kemnitz‐Hassanin, Christian P. R. Hackenberger

**Affiliations:** ^1^ Department of Chemical Biology Leibniz‐Forschungsinstitut Für Molekulare Pharmakologie (FMP) Berlin Germany; ^2^ Department of Chemistry Humboldt Universität zu Berlin Berlin Germany

**Keywords:** activity‐based probes, phosphatase, phosphorus, proteomics, proximity‐induced reactivity

## Abstract

Protein tyrosine phosphatases (PTPs) represent an important pharmacological target and subject of study. Although a number of broad‐spectrum electrophilic, phosphotyrosine‐mimicking probes have been developed to covalently capture the catalytic site of these enzymes, there is still a high demand for PTP probes with high target selectivity that are accessible in a synthetically straightforward way. Unsaturated phosphorus (V) (P(V)) compounds have recently emerged as powerful cysteine‐selective bioconjugation reagents (P5‐labeling). Herein, we introduce ethynyl‐substituted aryl phosphonamidic and phosphonic acids as phosphotyrosine mimics, which serve as active‐site‐directed, covalent probes for tyrosine phosphatases. We show that these P(V) electrophiles can be readily incorporated into a peptide sequence, allowing proximity‐enabled reactivity and selective targeting of the catalytic cysteine residue of an interacting phosphatase, as exemplified for PTP1B, a protein tyrosine phosphatase that acts as a key negative regulator of insulin signaling. Both ethynyl phosphonamidic acid and ethynyl phosphonic acid show no reactivity towards nontarget cysteine residues, though the phosphonamidic acid probe was notably less reactive toward its intended target. Proteomics experiments in human cell lysates demonstrated that the phosphonic acid probe selectively enriches its interacting phosphatase in the human proteome. Our study highlights a versatile strategy to obtain remarkably precise peptide‐based PTP probes, thereby enabling the characterization of phosphatase interactions with high specificity.

## Graphical abstract

Charged aryl‐ethynyl phosphonamidic and phosphonic acids are introduced as low‐reactivity electrophiles for peptide‐based activity probes that enable selective, target‐specific profiling of protein tyrosine phosphatases. The probes show no off‐target cysteine reactivity and engage only the interacting phosphatase in global proteomic analysis.

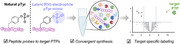

## 1 Introduction

Protein tyrosine phosphorylation is among the most important post‐translational modifications of proteins. This process is regulated by the synergistic effect of protein tyrosine kinases (PTKs) and phosphatases (PTPs) [1, 2]. Deviant tyrosine phosphorylation levels play a crucial role in protein function, signaling, health, and disease [3, 4, 5, 6]. Although tyrosine kinases have been extensively studied and targeted for therapy [7, 8], their counteracting partners for dephosphorylation gained interest only decades later [9, 10, 11, 12]. PTPs constitute the largest family of phosphatases [13], which share the signature catalytic motif HCX_5_R in which the cysteine residue is the essential nucleophile for catalysis [14]. This conservation of the active site has been one of the challenges to successfully study and target individual PTPs with high selectivity [12].

**FIGURE 1:**
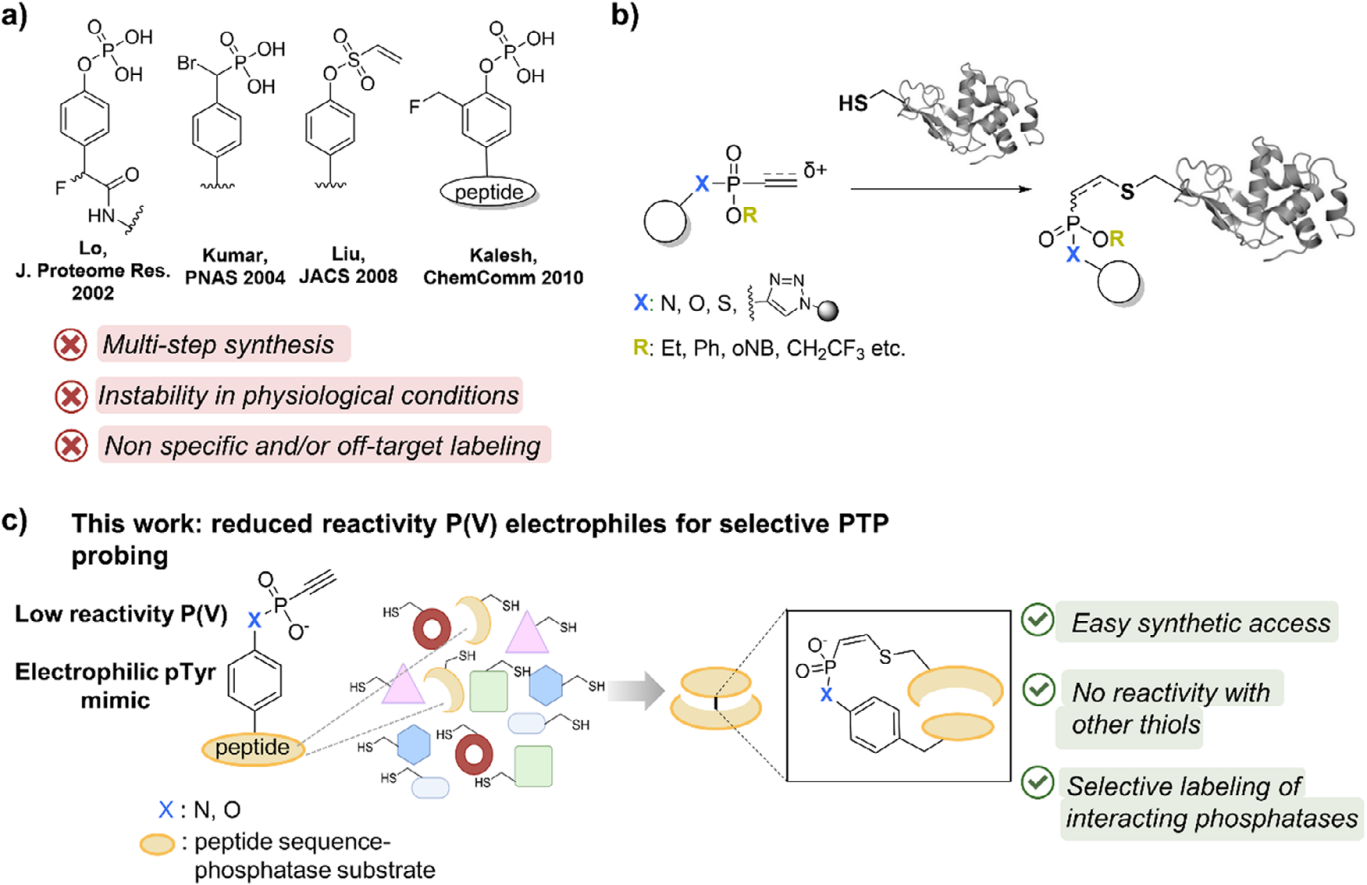
(a) Previous probes to target PTPs. (b) P5‐labeling for protein‐bioconjugation. (c) This work: Unsaturated P(V)‐electrophiles for specific PTP‐targeting.

A powerful technique that has found diverse applications in studying protein function, including PTPs, is activity‐based protein profiling (ABPP) [15, 16]. There, activity‐based probes (ABPs) are designed to covalently bind the protein of interest through an electrophilic moiety, and these stable adducts can be analyzed via several proteomic workflows [17]. By applying ABPP, significant developments have occurred in global PTP profiling employing broadly reactive probes. The first attempt for such class‐selective probes for PTPs was contributed by Lo and co‐workers by using a 4‐fluoromethylphenylphosphate (FMPP) reactive group (Figure 1a) [18]. Upon hydrolysis of the phosphate group by PTPs, an electrophilic quinone methide is released that can be captured by a proximal nucleophile. Nevertheless, the highly reactive nature of the quinone methide can lead to poor PTP specificity in a crude proteome [19, 20]. Kumar et al. designed ABPs with an α‐bromobenzylphosphonate (BBP) moiety (Figure 1a), which directly forms a covalent adduct with the active site cysteine [21, 22]. BBP‐bearing probes proved to be eminently PTP selective; however, significant hydrolysis took place at physiological pH values. Moreover, Liu and colleagues implemented phenyl vinyl sulfones (PVS) and sulfonates (PVSN) (Figure 1a) in active‐site irreversible probes for PTPs, which proved to be a useful tool for broad PTP profiling, as in the previous examples [20]. Although these tools advanced the collective study of PTPs within the proteome, the need for the development of covalent probes that would enable selectivity for an individual phosphatase remained largely unmet. To address this challenge, a study by Kalesh et al. focused on developing peptide‐based ABPs for potentially studying PTPs in a target‐specific manner. Toward this objective, the authors synthesized a 2‐fluoromethylphosphotyrosine (2‐FMPT) amino acid building block (Figure 1a), and incorporated it into several peptide substrates of PTPs, yet the most promising probe labeled several other proteins in human lysate [19]. It is important to highlight that significant progress has also been noted in the field, with the development of non‐covalent probes [23, 24, 25]. While these efforts marked a significant development in the targeted study of PTPs, recent studies uncovered major limitations of ABPs with the aforementioned reactive groups. Specifically, proteomics experiments revealed widespread off‐target labeling, in some cases involving over a thousand nonphosphatase proteins [26]. In view of this, ABPs bearing a novel reactive handle with higher selectivity and specificity for the cognate target remain elusive.

Previously, our group has introduced cysteine‐selective unsaturated P(V)‐electrophiles (P5‐labeling, Figure 1b) as versatile bioconjugation reagents to access protein‐protein and antibody‐drug conjugates [27, 28, 29, 30, 31, 32, 33]. These reagents display highly tunable reactivity depending on the nature of the unsaturated bond as well as the R and X substituents around the phosphorus atom (Figure 1b) [34, 35]. Recently, we have used less reactive vinyl phosphonamidates for ligand‐directed cysteine labeling to apply these reagents beyond bioconjugation [36].

Here, we aim to take advantage of the unique molecular composition of ethynyl phosphonamidic (**PN**) and phosphonic (**PO**) acids to enable their use as new covalent probes for PTP profiling. We hypothesized that these P(V)‐acids are deprotonated at physiological conditions, resulting in a negatively charged P─O substituent [37, 38]. This aspect would add electron density to the phosphorus center, thus rendering the ethynyl‐substituent less electrophilic and suitable for ABPP rather than bioconjugation. Furthermore, the negatively charged P(═O)─O moiety would offer stronger resemblance to a naturally occurring phosphotyrosine residue.

By taking advantage of a convergent synthetic strategy, we can incorporate the P(V)‐acids into a peptide sequence derived from a PTP substrate in a straightforward manner, without requiring a synthetically challenging building block. This approach enables access to electrophilic peptides that selectively target individual PTPs rather than acting as broad‐spectrum reactivity probes. We show that the unsaturated P(V)‐electrophile forms a covalent bond with the active site cysteine through proximity‐induced labeling (Figure 1c). Proteomic analysis showed superior performance of the phosphonic acid peptide probe to selectively label the designated phosphatase partner in human lysate.

## 2 Results and Discussion

### 2.1 Design and Synthesis of the Probes

We started our studies with the synthesis of ethynyl phosphonamidic (**PN**) and phosphonic (**PO**) acid‐containing peptides and selected an established phosphatase‐substrate pair as a proof of concept. Thereby, we focused on protein tyrosine phosphatase 1B (PTP1B) as it is one prominent member of classical PTPs [39, 40]. PTP1B has been implicated in diseases like diabetes and cancer and has gained a lot of interest as a pharmacological and study target [24, 41, 42, 43]. The substrate selected for our investigation is a peptide derived from one of the autophosphorylation sites in epidermal growth factor receptor (EGFR), specifically the amino acids 988–998 (DADEpYLIPQQG), due to the well‐characterized interaction with PTP1B, which catalyzes the hydrolysis of the pTyr residue [44].

**FIGURE 2:**
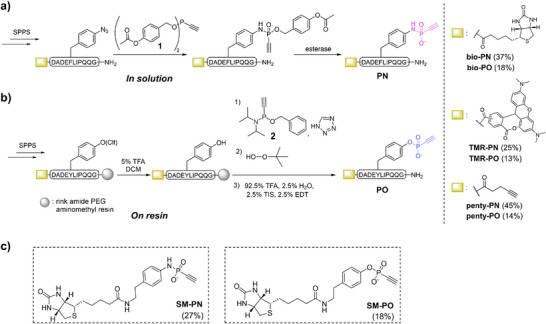
(a) Synthetic route to access **PN** peptide probes starting with a Staudinger‐phosphonite reaction on the peptide to produce a protected phosphonamidate peptide, which releases the free acid upon esterase treatment. (b) On‐resin approach for synthesizing the **PO** peptide probes via the reaction of the selectively deprotected peptide on tyrosine and compound **2**. (c) Structures of the equivalent to the peptides' small molecule controls (synthesis in Supporting Information, Section 3.3).

The synthesis of the **PN** probes was carried out according to published protocols of our group to generate ethynyl phosphonamidates via the Staudinger‐phosphonite reaction (SPhR) between an azide and an ethynyl phosphonite [45, 46, 47, 48]. Therefore, a para‐azido‐phenylalanine residue was incorporated into the sequence in place of pTyr via standard Fmoc solid‐phase peptide synthesis (SPPS). To achieve the final **PN** peptide, we aimed to synthesize an ethynyl‐phosphonite with cleavable substituents that enabled phosphonamidate synthesis and deprotection in solution at physiological pH to ensure integrity of the P‐N bond, analogously to the chemoselective synthesis of labile pLys peptides via Staudinger‐phosphite reactions [49]. Now, we opted for 4‐acetoxybenzyl‐ethynyl‐phosphonite **1** with esterase cleavable groups [50]. Notably, phosphonite **1** proved to be air‐stable and could be stored for 2 months at −20°C, unlike other previously reported phosphonites that required protection prior to handling [45, 46]. Subsequent reaction of **1** with an azido‐containing peptide, followed by treatment with esterase from porcine liver, yielded the final ethynyl phosphonamidic acid **(PN)** peptides. Specifically, we obtained the peptides **bio‐PN**, **TMR‐PN** and **penty‐PN** carrying biotin, tetramethylrhodamine, or a pentynoic handle at the N‐terminus of in good overall yields of 25%–45% for the following experiments (see Figure 2a and Supporting Information, Section 3.2).

To synthesize the **PO** probes, we developed a “one‐pot” approach, in which the installation of the phosphorus electrophile would occur on the solid support. We used a chlorotrityl‐(Clt)‐protected tyrosine residue during Fmoc‐SPPS on a resin, which could be selectively deprotected using 5% TFA, followed by 1*H*‐tetrazole‐mediated reaction with crude phosphonamidite **2** [51, 52]. Testing different solid supports for optimal swelling properties, we found that rink amide PEG aminomethyl resin showed optimal performance and high conversions in the reaction of **2** with the peptide (Figure S1). Oxidation of the intermediately formed phosphonite‐containing peptide with tert‐butyl peroxide, followed by global cleavage from the resin, led to the final product PO peptide, delivering **bio‐PO**, **TMR‐PO**, and **penty‐PO**, again in good overall yields of 13%–18% (Figure 2b). Additionally, we synthesized the corresponding small molecule phosphonamidic and phosphonic acids, abbreviated **SM‐PN** and **SM‐PO**, to test whether the peptide sequence guides the proximity‐induced reactivity and target specificity (Figure 2c, synthetic procedure in Supporting Information, Section 3.3).

### 2.2 Stability and Reactivity Studies

Next, we assessed the stability of the phosphonamidic and phosphonic acid in aqueous buffers at pH 2, pH 3.5, pH 7.4, pH 8.5, and pH 11. Therefore, **SM‐PN** and **SM‐PO** were added in the corresponding buffers and monitored for 48 h at room temperature via ^31^P‐NMR with triphenylphosphinoxide (TPPO) as an internal standard (Figure S2). The phosphonic acid group exhibited excellent stability in all the above‐mentioned conditions, as expected since phosphonates typically require harsh conditions to be hydrolyzed [53]. Phosphonamidates, on the other hand, demonstrate poor stability in acidic environments [28, 54, 55]. We observed about 80% hydrolysis of the starting material **SM‐PN** at pH 3.5, while at pH 2, the compound was already hydrolyzed in a few minutes before the measurement was completed (Figure S2), which is in accordance with previous observations for other phosphonamidic acid derivatives [38]. Furthermore, we evaluated the stability of the peptide probes **bio‐PN** and **bio‐PO** in human lysate for subsequent experiments. These peptides were added in HEK293T cell lysate, and changes in stability were recorded for 18 h via ^31^P‐NMR, in which inorganic phosphate served as an internal standard. The probe **bio‐PO** stayed unaltered for the experimental period, while 80% of the **bio‐PN** remained intact (Figure S3). Nonetheless, both peptides display sufficient stability for follow‐up studies.

**FIGURE 3:**
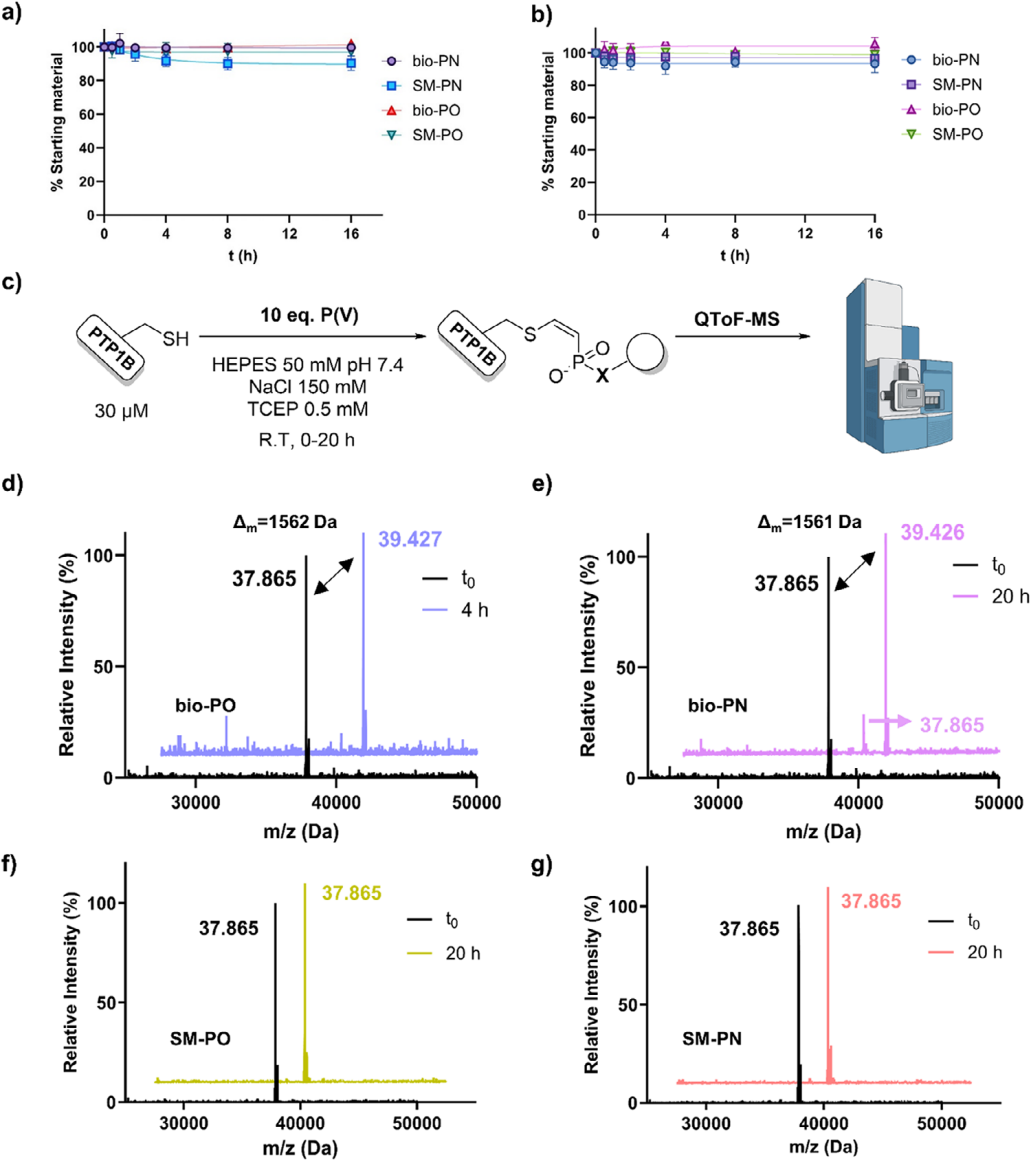
Time‐dependent decay of **bio‐PN**, **bio‐PO**, **SM‐PN**, and **SM‐PO** (1 mM) in the presence of 10 mM GSH with 1 mM caffeine or TPPO as internal standard. The assay was performed at 25°C in Tris buffer (10% DMSO) at (a) pH 7.4, (b) pH 8.5. Results are presented as mean ± standard deviation (*n* = 3). (c) Labeling conditions to assess by ESI‐MS the ability of **bio‐PN**, **bio‐PO**, **SM‐PN**, and **SM‐PO** to covalently bind recombinant PTP1B. Stacked deconvoluted MS spectra of the initial time point (t0) of PTP1B labeling with (d) bio‐PO after 4 h: full labeling, (e) **bio‐PN** after 20 h: ∼80% labeling, (f) SM‐PO after 20 h: no labeling, (g) **SM‐PN** after 20 h: no labeling.

After verifying the stability of the phosphonamidic and phosphonic acid warheads at physiological and slightly basic pH, we aimed to investigate the reactivity towards endogenous thiol concentrations. For this, 1 mM **bio‐PN** or **bio‐PO** and 1 mM caffeine as an internal standard, as well as 1 mM **SM‐PN** or **SM‐PO** and 1 mM TPPO (internal standard), were incubated at room temperature with 10 equivalents (10 mM) glutathione (GSH), and the outcome was followed by UPLC‐MS (Figure S4). For both peptide and small molecule phosphonamidic acids and phosphonic acids, no consumption of the starting material to a glutathione adduct was observed over the course of 16 h, either at pH 7.4 (Figure 3a) or at pH 8.5 (Figure 3b). Previously reported ethynyl phosphonamidates and phosphonates from our group, where the phosphorus ester has an ethyl substitution, showed relatively high reaction kinetics with GSH [28, 29], confirming our hypothesis that the thiol reactivity is abolished by removing this alkyl group.

Intrigued by this observation, we aimed to evaluate their ability to label the intended target, namely PTP1B. Recombinant PTP1B in a suitable buffer (HEPES pH 7.4, supplemented with 150 mM NaCl and 0.5 mM TCEP to keep the catalytic cysteine reduced) was reacted with 10 equivalents of the biotinylated peptide probes or the small molecule controls at 25°C and the progress was analyzed by QToF high resolution mass spectrometry (Figure 3c). Notably, incubation of peptide **bio‐PO** with PTP1B for 4 h led to complete conversion to the enzyme with one peptide unit covalently attached (Figure 3d). The phosphonamidic acid peptide, **bio‐PN**, was also able to label the enzyme; however, a slower reaction was observed, as even after 20 h, about 80% of the covalent adduct could be observed (Figure 3e). Interestingly, previous comparative studies of the thiol addition to ethynyl phosphonamidates and phosphonates demonstrated the superior reaction speed of the latter [34]. We observed that neither **SM‐PO** (Figure 3f) nor **SM‐PN** (Figure 3g) showed a covalent addition to PTP1B, eluding towards the necessity of the peptide sequence to direct the electrophile to the catalytic pocket.

Moreover, we tested other cysteine‐containing proteins for covalent labeling by **bio‐PN** and **bio‐PO**. Prolonged incubation of 20 h with recombinant albumin or TEV protease, which has an activated cysteine in the catalytic triad [56], did not yield a covalent adduct for either of the probes (Figure S5).

Next, we investigated whether the electrophilic peptide probes **bio‐PN** and **bio‐PO** target the active‐site cysteine (Cys215) of PTP1B as opposed to the other five cysteines present in the sequence [57]. On that account, samples from the labeling reaction with PTP1B were drawn at different time intervals, resolved by SDS‐PAGE, and prepared for LC/MS‐MS measurements [58]. Indeed, we detected that the covalent modification from the ethynyl phosphonamidic and phosphonic acid peptides was located primarily at the catalytic cysteine (Cys215) and found to increase over time (Figure S6). Besides C215, we found mild reactivity towards a few other cysteines within PTP1B, which may be due to the flexibility of the probe on the substrate sequence, enabling it span wider distances.

### 2.3 Evaluation of Affinity and Kinetic Parameters

**FIGURE 4:**
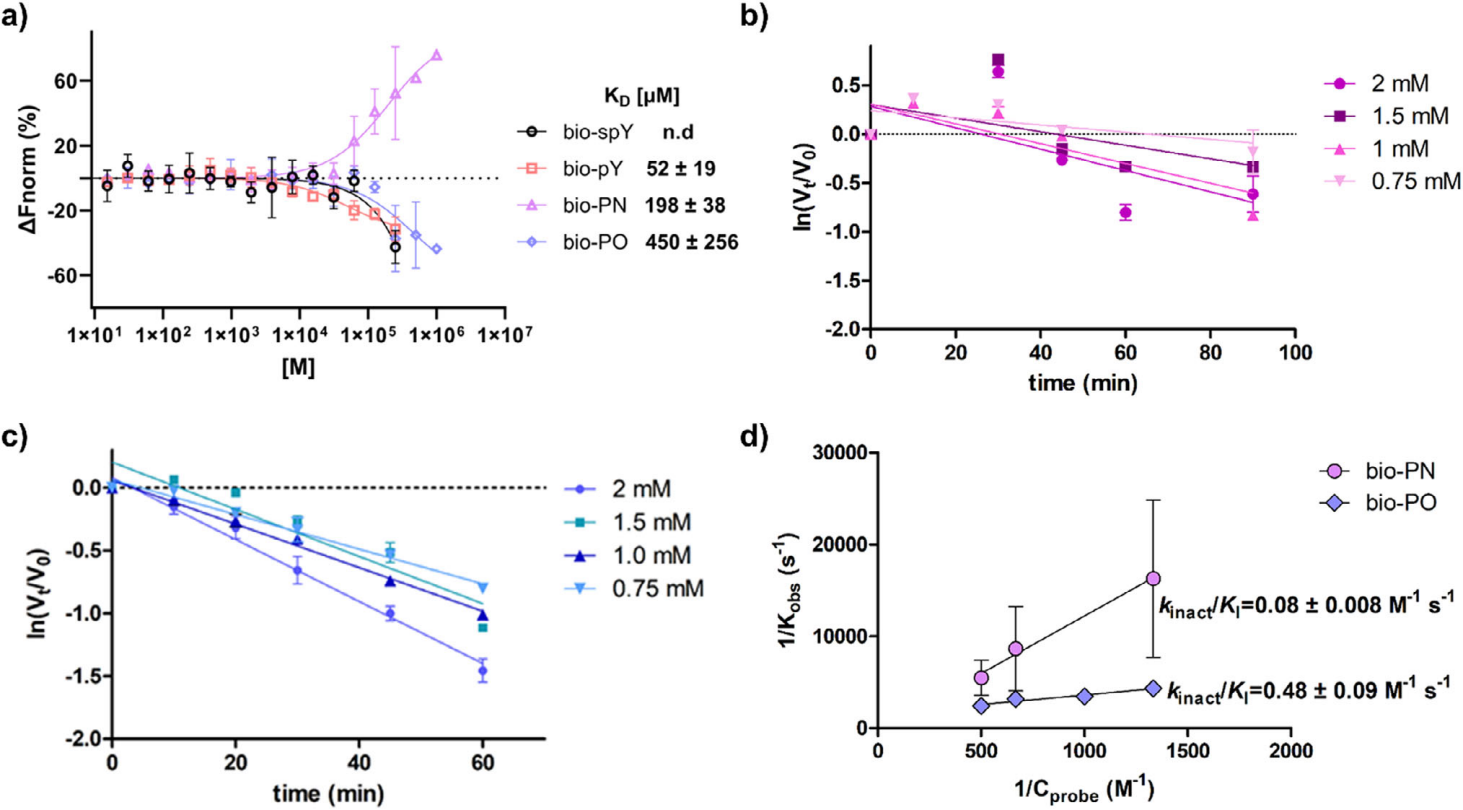
(a) MST binding curves and calculated K_D_ values with PTP1BC215A for all four peptides tested. Results are presented as mean ± standard deviation (*n* = 3–5). PTP1B inactivation graphs at the indicated probe concentrations and preincubation times of (b) **bio‐PN** and (c) **bio‐PO** as calculated by the pNPP assay. Each slope represents a k_obs_ value. Results are presented as mean ± standard error of the mean (*n* = 3). (d) Kitz–Wilson kinetic parameters (no saturation reached) of the covalent labeling between PTP1B and **bio‐PN** or **bio‐PO**. Errors were propagated from the inactivation plots.

Motivated by the successful covalent labeling of recombinant PTP1B, we desired to further evaluate the binding of the peptide probes. One crucial parameter for subsequent experiments in more complex environments is the affinity towards the enzyme. It is known that the negative charges of the phosphate ester play a significant role in substrate recognition and catalysis by interacting non‐covalently with important residues [59, 60, 61, 62]. In some of the previously reported PTP probes, the phosphate ester was implemented in the design of the warheads. Since we replaced one negatively charged oxygen atom with an ethynyl group, we performed microscale thermophoresis (MST) measurements to compare the affinities between the electrophilic peptide probes and the phosphatase. For this, a PTP1B mutant was employed, where the catalytic cysteine is replaced by alanine (PTP1B‐C215A) to exclude a covalent reaction and study only the non‐covalent peptide interaction. This mutant is catalytically inactive but retains its ability to bind phosphopeptides and has been widely used for substrate trapping experiments [63]. In addition, a natural phosphotyrosine (**bio‐pY**) as well as a nonbinding scrambled phosphotyrosine peptide (**bio‐spY**) with a biotin tag at the N‐terminus were synthesized for direct comparison (for structure and synthesis see Supporting Information, Section 3.2.2). No dissociation constant (K_D_) could be determined for the negative control **bio‐spY**, as no measurable binding was observed at any of the concentrations tested. The natural substrate **bio‐pY** exhibited a K_D_ value of 52 ± 19 µM, while **bio‐PN** and **bio‐PO** showed a considerably higher value of 198 ± 38 µM and 450 ± 256 µM, respectively (Figure 4a). These results demonstrate that replacing the negatively charged oxygen atom with the electrophilic alkyne significantly compromises the affinity of the substrate to the enzyme, leading to a four to eightfold increase in K_D_ values. Nevertheless, binding is retained, which contributed to the covalent capture of the wild type.

Following this, we aimed to determine the kinetic parameters of the covalent binding. The activity of PTP1B upon preincubation with **bio‐PN** and **bio‐PO** was measured by the commonly employed para‐nitrophenyl phosphate assay (*p*NPP) [64]. Consistent with irreversible binding, the inactivation of the enzyme by the probes followed concentration and time‐dependent kinetics (Figure 4b,c), and the pseudo‐first‐order rate constants k_obs_ could be extracted. Plotting k_obs_ versus probe concentration following the Kitz–Wilson kinetics [65] produced a linear relationship over the accessible concentration range without reaching a plateau. Therefore, only the composite second‐order efficiency *k*_inact_/*K*_I_ is identifiable, whereas these parameters cannot be estimated separately without approaching saturation kinetics [66]. The apparent second‐order rate constant (*k*_inact_/*K*_I_) was calculated to be 0.08 ± 0.008 M^−1^s^−1^ for **bio‐PN** and, about six times higher, 0.48 ± 0.09 M^−1^s^−1^ for **bio‐PO** (Figure 4d). These results are in accordance with the observed reactivity in PTP1B labeling monitored by QToF‐MS. As a reference, the second‐order rate constants for existing probes were calculated by their reported k_inact_ and K_I_ values. BBP probes exhibited a rate constant of 3.83 and 0.45 M^−1^s^−1^ with phosphatase YopH [21], PVS and PVSN showed a rate constant of 3.81 and 3.51 M^−1^s^−1^, respectively, also with YopH [20], while the most promising peptide 2‐FMPT probe demonstrated a second‐order rate constant of 12.2 M^−1^s^−1^ with PTP1B [19]. Although the **bio‐PO** probe remains comparatively slow, the kinetic profile is within an acceptable range for ABPP. In contrast, the **bio‐PN** probe falls on the lower end of the reactivity spectrum.

### 2.4 Pull‐Down Assays in Human Lysate

Moving forward, we tested the ability of the probes to label endogenous PTP1B in human lysates. Following the previous observations, only the **bio‐PO** probe was selected for further applications, as it exhibited more promising kinetic data. Given the delicate nature of PTP1B and its susceptibility to oxidative inactivation of the active site cysteine, we first set out to identify suitable conditions for cell lysate preparation (see Supporting Information and Figure S7). Having identified suitable conditions, we aimed to compare our target‐specific peptide **PO**‐probe with a broad reactivity PTP probe in order to visualize the different reactivity patterns in human lysate. Therefore, we probed the reactivity of **TMR‐PO** and Zhang's bromophosphonate probe, which was synthesized (Supporting Information, Section 3.3) with a tetramethylrhodamine tag (**TMR‐BBP**). Using the previously reported buffer conditions, HEK293T cell lysate (50 mM sodium succinate, pH 6.0, 150 mM NaCl, 1 mM EDTA, 1 mM DTT) was incubated for 1 h with **TMR‐BBP** (100 µM). Similarly, HEK293T cell lysate (with Tris buffer 50 mM, pH 7.4, 150 mM NaCl, 0.5 mM TCEP) was treated with **TMR‐PO** (100 µM), and both samples were resolved with SDS‐PAGE and visualized by fluorescence gel scanning. Consistent with our expectations, **TMR‐BBP** labeled many proteins across the whole proteome, while very distinct bands were observed for **TMR‐PO** (Figure S8), indicating superior selectivity.

To verify a consistent labeling profile by **TMR‐PO**, we tested additional PTP1B‐containing mammalian lysates. Interestingly, treatment of three different human lysate samples (HEK293T, MCF‐7, and Ramos) with **TMR‐PO** showed a comparable labeling pattern (Figure S9), although the overall degree of labeling appeared low. Therefore, we attempted to identify conditions where more intense labeling is noticeable. Given the low *k*_inact_/*K*_I_ we observed before, we incubated **bio‐PO** (100 µM) with HEK293T, MCF‐7, and Ramos lysate for up to 18 h, and blotted for both biotin and PTP1B (Figure S10). Increasing reaction time led to a proportional increase in biotin‐signal overlapping with the PTP1B band. It is relevant to highlight that in Ramos cell lysates, both signals exhibited very low intensity. Notably, no unspecific labeling was observed even at prolonged incubation times. A 4‐h incubation was chosen for the following experiments for ease of protocol and due to the robust labeling observed.

**FIGURE 5:**
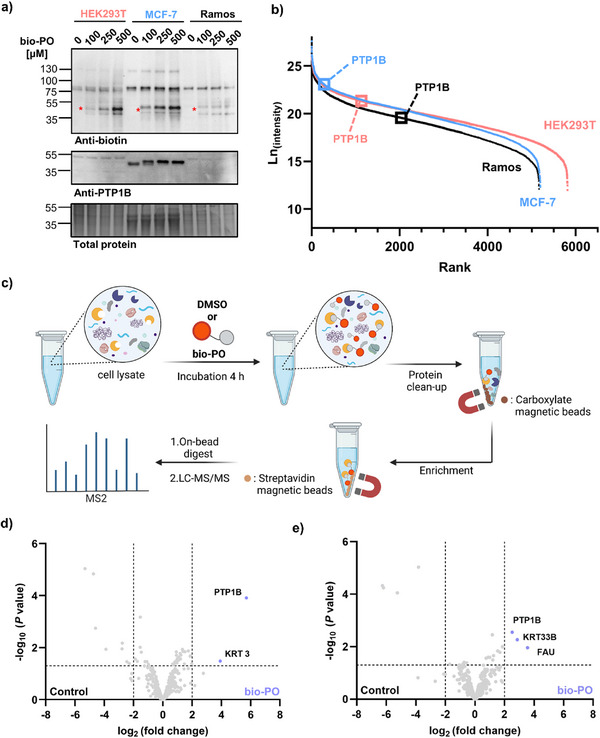
(a) Western blot analysis of three different human lysates incubated with varying concentrations of **bio‐PO**. Upper panel: biotin signal—putative PTP1B bands are marked with an asterisk. Middle panel: PTP1B signal in three different lysates. Lower panel: total protein staining of all proteins loaded. (b) Intensity‐Based Quantification plot for estimating PTP1B content in each of the three lysates. (c) Proteomic workflow for lysate labeling (400 µg) with **bio‐PO** (400 µM). (d) Volcano plot of proteomic profiling of MCF‐7 lysate with **bio‐PO** (right side) and DMSO (left side) as a control (enrichment >2 log_2_ fold change, *p* ≤ 0.05). (e) Volcano plot of proteomic profiling of HEK293T lysate with bio‐PO (right side) and DMSO (left side) as a control (enrichment >2 log_2_ fold change, *p* ≤ 0.05). Proteomics experiments were performed in biological triplicate.

In that respect, following a 4‐h incubation time, we tested increasing concentrations of **bio‐PO** up to 500 µM to further increase the labeling efficiency. By analyzing the biotin signal, a correlative increase in band intensity was noted upon increasing probe concentration. The most prominent probe signal was observed in MCF‐7 cells, followed by HEK293T, while Ramos showed low signal bands (Figure 5a). This highlights that labeling is also consistent with the abundance of PTP1B in these cell lines, which was measured by quantitative proteomics (Figure 5b). Again, no unspecific bands appeared at high concentrations of **bio‐PO**. The higher molecular weight bands observed in the biotin signal are also present in the DMSO control.

To verify that our probe is actually targeting the active site of PTP1B, we treated HEK293T and MCF‐7 lysate with 500 µM **bio‐PO** for 4 h in the presence of H_2_O_2_. As anticipated, treatment with the oxidizing reagent abolished labeling, due to oxidation of the active site cysteine (Figure S11).

To further test our hypothesis of the specific labeling arising as a result of the low reactivity warhead in combination with the peptide substrate, the corresponding *O*‐ethyl (OEt) substituted ehtynyl phosphonate group was installed on the exact same peptide sequence (Figure S12a, synthesis in Section 3.2). This **bio‐PO(OEt)** derivative showed significantly higher labeling across the whole MCF‐7 proteome, even with lower concentration and incubation time, as shown in a western blot experiment (Figure S12b). This result further supports the need for a low reactivity electrophile as the reactive group.

Finally, we employed bottom‐up proteomics to validate that the labeled protein corresponds to the intended target PTP1B. For this, first MCF‐7 cell lysate (400 µg) was treated with 500 µM **bio‐PO** for 4 h. Directly afterwards, we applied a protocol described by the Kielkowski group, termed SP2E, which integrates protein purification with magnetic carboxyl‐coated beads and enrichment using magnetic streptavidin beads (Figure 5c) [67]. Following this protocol, we were able to show that PTP1B was the most significantly enriched (>32‐fold) protein from MCF‐7 lysate (Figure 5d). Keratin (KRT3) was also highly enriched, though keratins are well‐known contaminants and not considered biologically relevant here [68]. Interestingly, there were no other proteins exhibiting such high enrichment, and more importantly, no other phosphatases were enriched, even though they were present in the proteome. Applying the exact same workflow to HEK293T cell lysate, we did not observe enrichment of PTP1B. We reasoned that this might be due to the lower abundance of PTP1B in HEK293T compared to MCF‐7 lysate. Therefore, the protocol was repeated by doubling the proteome input to 800 µg. Following this minor adjustment, PTP1B was then enriched in our analysis (>4‐fold) (Figure 5e). In this case, together with the desired target and a keratin contaminant, another protein was found enriched in the **bio‐PO**‐treated sample (Figure 5e), ubiquitin‐like and ribosomal protein S30 fusion (gene name: FAU). However, this protein is included in the contamination repository for affinity purification (CRAPome) [69] and appears in about half of such experiments as a contaminant. Given the comparably low PTP1B levels in Ramos lysate, we refrained from attempting pull‐down experiments from this cell line. According to our understanding, no other probe has been shown via global proteomic analysis to engage PTP1B without targeting any other phosphatase and, more importantly, PTPN2, also known as TCPTP, which bears more than 70% sequence homology with PTP1B and also acts as a modulator of glucose homeostasis and insulin sensitivity [70, 71].

This established selectivity of the PO peptide, together with the catalytic‐cysteine engagement and the loss of labeling under H_2_O_2_ treatment, suggests that our probes can be used to study active phosphatase pools in conditions where activity may change independently of expression. For example, this is relevant in settings such as during insulin‐induced or oxidative inactivation of PTP1B [72, 73, 74] or upon pharmacological inhibition of the active site. Comparable redox‐sensitive behaviour is well documented for other cysteine‐dependent phosphatases, including TCPTP [75], and the Low Molecular Weight‐PTPs (LMW‐PTPs) [76], making them suitable candidates for analogous peptide probes.

Finally, we probed the reactivity of **SM‐PN** and **SM‐PO** (25–500 µM, 4 h) in MCF‐7 lysate. No labeling was observed by the control **SM‐PN**, while for **SM‐PO**, a band with increasing intensity was apparent in the biotin signal upon concentration increase. This band overlapped with the most prominent one in the total protein stain signal at around 50 kDa, so we attributed it to nonspecific labeling likely due to the high abundance of proteins present in that region (Figure S13). To confirm that the labeling taking place in MCF‐7 lysate upon incubation with **SM‐PO** is nonspecific, we applied the same proteomic workflow as before, and neither PTP1B nor any other phosphatase was significantly enriched (Figure S14a). Instead, we detected several proteins found as contaminants in the CRAPome. In addition, adipocyte plasma membrane–associated protein (APMAP) was identified among the enriched proteins. Given its molecular weight of approximately 47 kDa, the presence of three cysteines, and its high abundance in MCF‐7 lysate (Figure S14b), it may account for the _∼_50 kDa band detected in the western blot. The lack of phosphatase enrichment upon labeling of the lysate with **SM‐PO** highlights the necessity of the peptide sequence for target engagement again.

## 3 Conclusion

In this study, we report novel activity‐based probes for target‐specific capture of tyrosine phosphatases. We designed cysteine‐reactive ethynyl phosphonamidic (**PN**) and phosphonic acid (**PO**) motifs as phosphotyrosine mimics. These mimics were integrated into a PTP1B peptide substrate sequence to probe proximity‐induced reactivity. The synthetic protocols take advantage of convergent strategies in solution or on the solid support, thus preventing the laborious synthesis of a building block. We were able to show the covalent modification of recombinant PTP1B with both peptide probes, while glutathione or other cysteine‐containing proteins remained unreactive. To study the labeling pattern in more complex samples, such as human lysate, **PO**‐peptides were used due to more favorable kinetics. We showed that the **PO** probe selectively enriches the intended target, PTP1B, across the whole proteome of MCF‐7 and HEK293T cell lysate. These findings indicate that combining our phosphorus‐based electrophiles with the peptide sequence of a phosphatase substrate places these probes within the narrow reactivity window needed for specific targeting.

Despite significant advances, the challenge to selectively probe individual PTPs remains stark. In view of the straightforward and practical nature of our protocol, as well as the achieved specificity, we envision our ethynyl phosphonic acids as a versatile chemical tool that, when installed on the desired substrate, could lead to the identification of novel phosphatase‐interaction partners. Future directions will also include efforts to label the corresponding enzyme in live cells, either by using simple electroporation for probe internalization or by applying our group's newly published cell‐penetrating delivery method, bioRAM [77]. The latter approach would only require the introduction of a linker–lysine motif into the peptide substrate sequence. Moreover, an ethynyl‐phosphonic‐acid–modified tyrosine residue may function as a noncanonical amino acid for genetic code expansion and, by adopting the established immunoprecipitation and MS/MS procedure described by Tang et al. [78], could allow selective crosslinking of phosphatases to their interactors in living cells.

## Conflicts of Interest

The authors declare no conflicts of interest.

## Supporting information




**Supporting File 1**: The authors have cited additional references within the Supporting Information [79, 80, 81, 82, 83, 84, 85, 86, 87, 88].

## Data Availability

The mass spectrometry proteomics data have been deposited to the ProteomeXchange Consortium via the PRIDE [89] partner repository with the dataset indentifier PXD068999.
