# Supplementary material for: Unsaturated Phosphorus Electrophiles to Probe Protein Tyrosine Phosphatases
Source: Angew Chem Int Ed Engl. 2026 Feb 3;65(11):e21902. doi: 10.1002/anie.202521902 (PMC12970513; doi:10.1002/anie.202521902)

**Supporting Information**

**Unsaturated Phosphorus Electrophiles to Probe Protein Tyrosine Phosphatases**

Eleftheria Poulou^[a][b]^, Max Ruwolt^[a]^, Christian E. Stieger^[a]^, Kristin Kemnitz-Hassanin^[a]^, Christian P. R. Hackenberger*^[a][b]^

[a] Eleftheria Poulou, Max Ruwolt, Christian E. Stieger, Kristin Kemnitz-Hassanin, Christian P. R. Hackenberger
Leibniz-Forschungsinstitut für Molekulare Pharmakologie (FMP)
Robert-Rössle- Straße 10, 13125 Berlin (Germany)
E-Mail: [hackenbe@fmp-berlin.de](mailto:hackenbe@fmp-berlin.de)

[b] Eleftheria Poulou, Christian P. R. Hackenberger
Department of Chemistry, Humboldt Universität zu Berlin
Brook-Taylor-Straße 2, 12489 Berlin (Germany)

**Reviewer Access Information (for peer review only – to be removed after acceptance)**

PRIDE accession number: **PXD068999**
Username: **reviewer_pxd068999@ebi.ac.uk**
Password: **TKJJW9UBx4Xk**

**Table of contents**

[1 Supplementary Figures 5](#_Toc214395734)

[2 General Information 19](#_Toc214395735)

[2.1 Chemicals and solvents 19](#_Toc214395736)

[2.2 Flash- and thin layer chromatography 19](#_Toc214395737)

[2.3 Semi-reparative HPLC 19](#_Toc214395738)

[2.4 NMR spectroscopy 19](#_Toc214395739)

[2.5 Cell culture 19](#_Toc214395740)

[2.6 Protein concentration determination 19](#_Toc214395741)

[2.7 Size-exclusion chromatography 19](#_Toc214395742)

[2.8 UPLC-UV/MS 20](#_Toc214395743)

[2.9 HR-MS 20](#_Toc214395744)

[2.10 Intact protein MS 20](#_Toc214395745)

[3 Experimental procedures and characterization data 21](#_Toc214395746)

[3.1 Protein expression and purification 21](#_Toc214395747)

[3.2 Peptide synthesis 23](#_Toc214395748)

[3.2.1 Procedure for phosphonamidate-based peptides 23](#_Toc214395749)

[3.2.2 Procedure for phosphonate-based peptides 31](#_Toc214395750)

[3.3 Organic synthesis 37](#_Toc214395751)

[3.3.1 Di-(4-acetoxy benzyl) ethynylphosphonite (1) 37](#_Toc214395752)

[3.3.2 1-(benzyloxy)-1-ethynyl-*N,N*-diisopropylphosphanamine (2) 38](#_Toc214395753)

[3.3.3 1-ethoxy-1-ethynyl-*N,N*-diisopropylphosphanamine (3) 38](#_Toc214395754)

[3.3.4 Synthetic route to SM-PN 39](#_Toc214395755)

[3.3.5 Synthetic route to SM-PO 41](#_Toc214395756)

[3.3.6 Synthetic route to TMR-BBP (reported phosphatase warhead) 44](#_Toc214395757)

[3.4 pH and lysate stability studies 47](#_Toc214395758)

[3.5 Thiol reactivity studies with glutathione 48](#_Toc214395759)

[3.6 Recombinant protein labeling 48](#_Toc214395760)

[3.7 LC-MS/MS for labeling site identification 49](#_Toc214395761)

[3.8 Microscale thermophoresis (MST) 50](#_Toc214395762)

[3.9 Kinetic characterization by *p*NPP assay 50](#_Toc214395763)

[3.10 Labeling in human lysate – gel scanning and western blotting 51](#_Toc214395764)

[3.10.1 General procedure for fluorescence gel scanning and western blotting 51](#_Toc214395765)

[3.10.2 Optimization of lysis conditions 52](#_Toc214395766)

[3.10.3 Further evaluation using TMR-PO 52](#_Toc214395767)

[3.10.4 Time course labeling using bio-PO 53](#_Toc214395768)

[3.10.5 Concentration dependent labeling using bio-PO 53](#_Toc214395769)

[3.10.6 Labeling of H_2_O_2_ treated lysate with bio-PO 53](#_Toc214395770)

[3.10.7 Lysate labeling with bio-PO(OEt) 53](#_Toc214395771)

[3.10.8 Lysate labeling with SM-PN and SM-PO 54](#_Toc214395772)

[3.11 Labeling in human lysate – LC-MS/MS-based proteomics 54](#_Toc214395773)

[3.11.1 Quantitative proteomic analysis for protein abundance 54](#_Toc214395774)

[3.11.2 Proteomic profiling of bio-PO 55](#_Toc214395775)

[3.11.3 Proteomic profiling of SM-PO 56](#_Toc214395776)

[4 Supplementary References 57](#_Toc214395777)

[5 Uncropped gels and western blots 59](#_Toc214395778)

[6 NMR Spectra 66](#_Toc214395779)

# **Supplementary Figures**


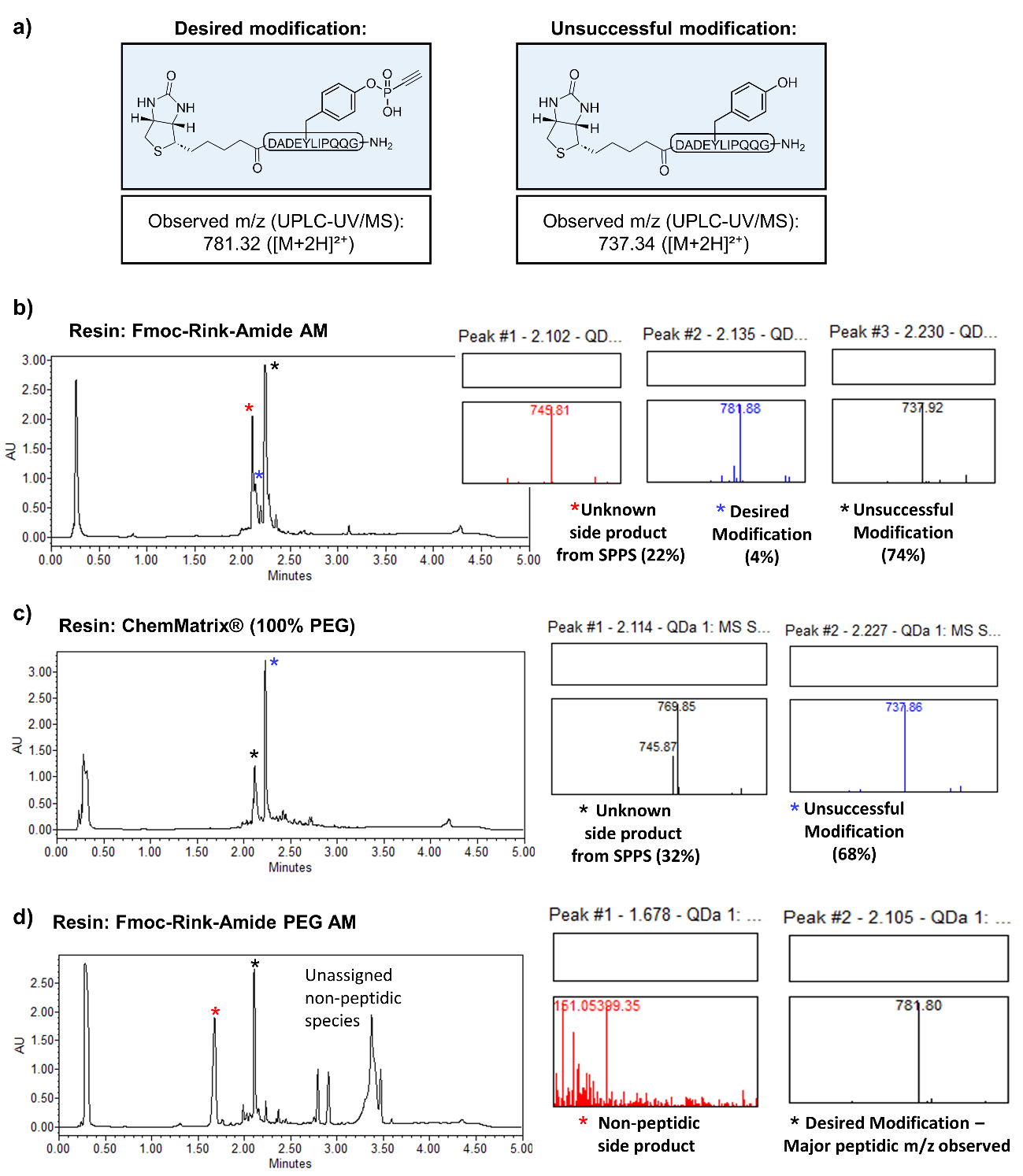


**Figure S1:** Overview of the optimization of the resin to establish a general procedure for phosphonic acid peptides. a) structures and calculated m/z (QDa, low resolution) for the desired product and the unmodified peptide. UPLC-UV/MS chromatograms and corresponding m/z information of the crude peptides synthesized on: b) Fmoc-Rink-Amide AM resin, c) ChemMatrix**®** (100% PEG) and d) Fmoc-Rink-Amide PEG AM resin.


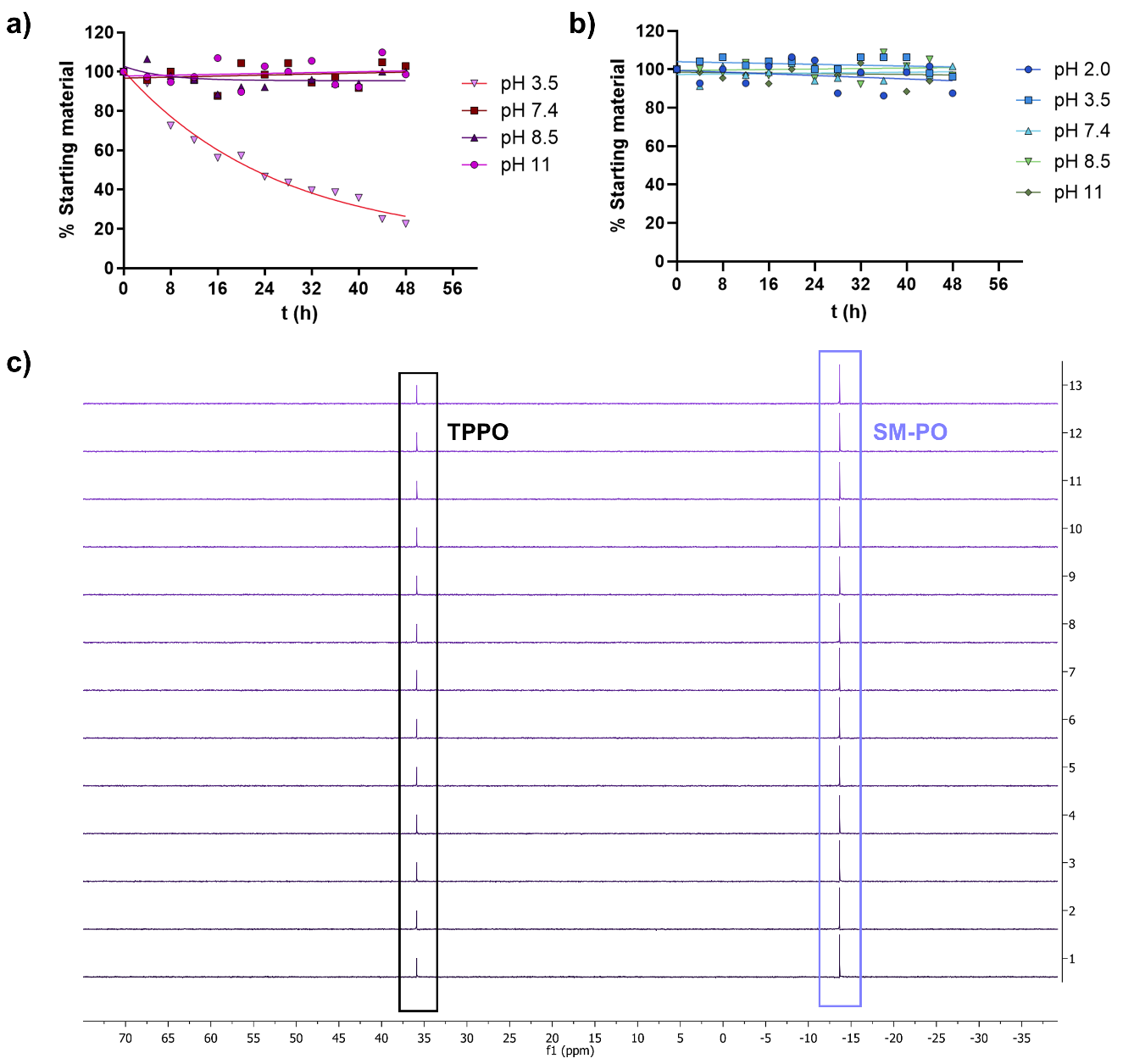


**Figure S2:** pH dependent stability of phosphonamidic and phosphonic acids at room temperature monitored by ^31^P NMR. a) Decay of starting material **SM-PN**. The compound shows good stability in neutral and basic pH and about 80% hydrolysis at pH 3.5 over 48 h. At pH 2.0 the compound was already hydrolyzed in minutes before the next measurement. b) Decay of **SM-PO** in identical conditions. **SM-PO** exhibits excellent stability in all tested pH values. c) Exemplary experimental set-up: stacked ^31^P NMR spectra of all measurements for **SM-PO** at pH 8.5 with highlighted signals of internal standard (TPPO) and the compound.

**
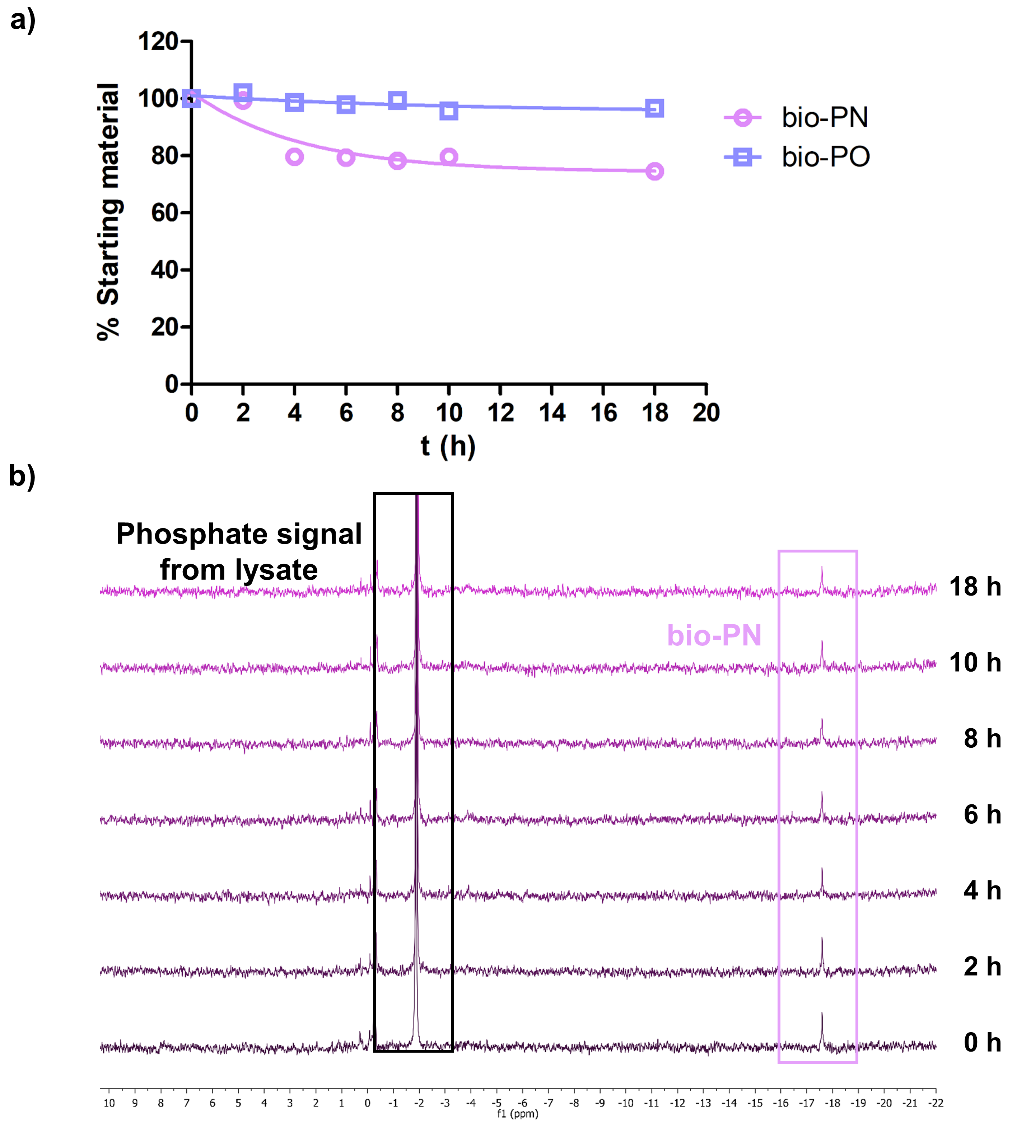
**

**Figure S3:** Stability of peptides **bio-PN** and **bio-PO** in HEK293T cell lysate. a) Decay of starting material **bio-PN** and **bio-PO** in lysate over the course of 18 hours at room temperature. A 20% decay of **bio-PN** is observed, while **bio-PO** remains intact. b) Exemplary experimental set-up: stacked ^31^P NMR spectra of all measurements for **bio-PN** with highlighted signals of internal standard (inorganic phosphate) and the compound.


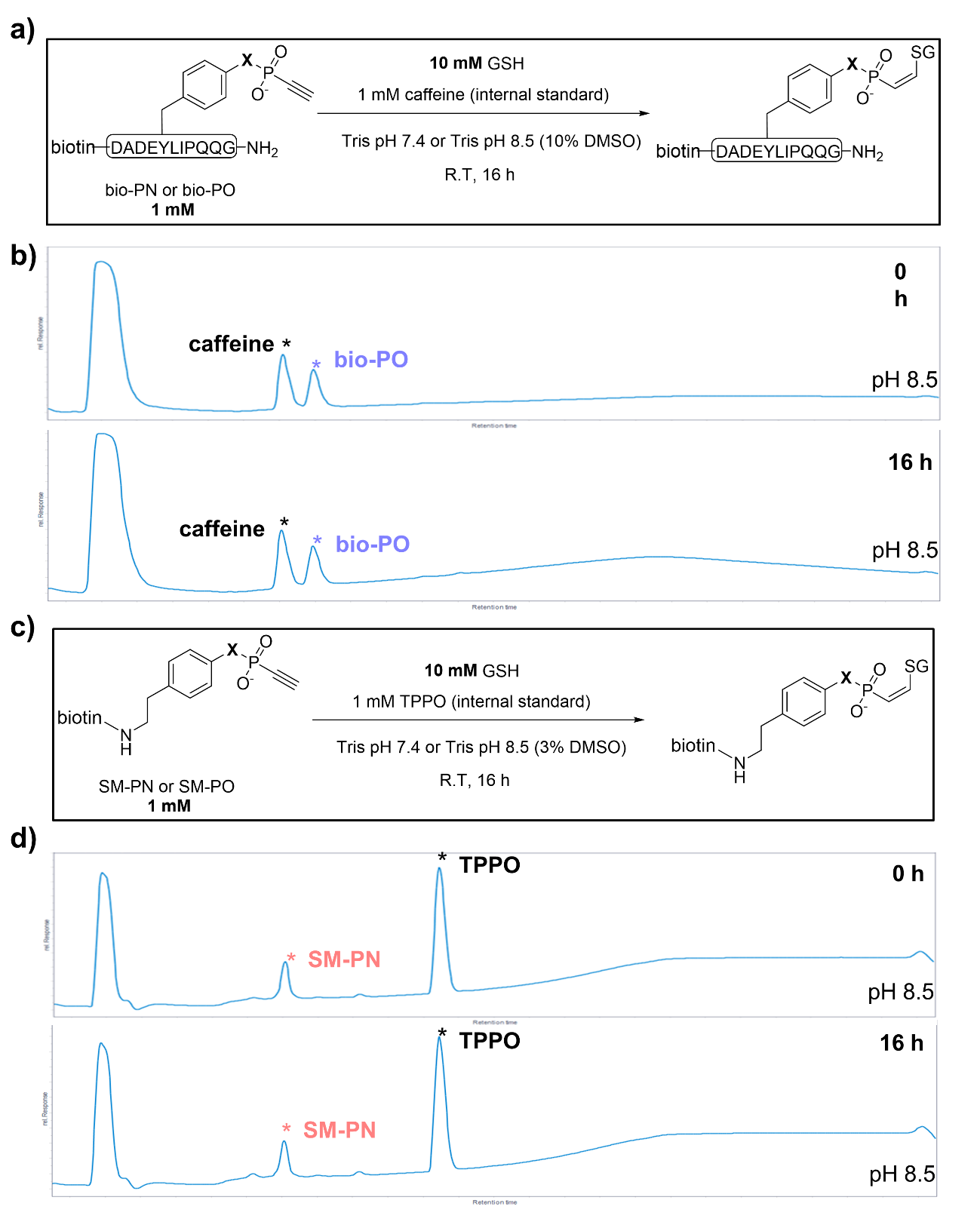


**Figure S4:** Monitoring of thiol reactivity with GSH as a model thiol of peptide and small molecule phosphonamidic and phosphonic acids by UPLC-UV/MS. a) Reaction conditions for **bio-PN** and **bio-PO**. b) Exemplary UV chromatograms of **bio-PO** at pH 8.5 and at 0 and 16 hours, highlighting the internal standard (caffeine) and the compound. c) Reaction conditions for **SM-PN** and **SM-PO**. d) Exemplary UV chrommatogramms of **SM-PN** at pH 8.5 and at 0 and 16 hours, highlighting the internal standard (TPPO) and the compound. All experiments were prepared in triplicates.

**
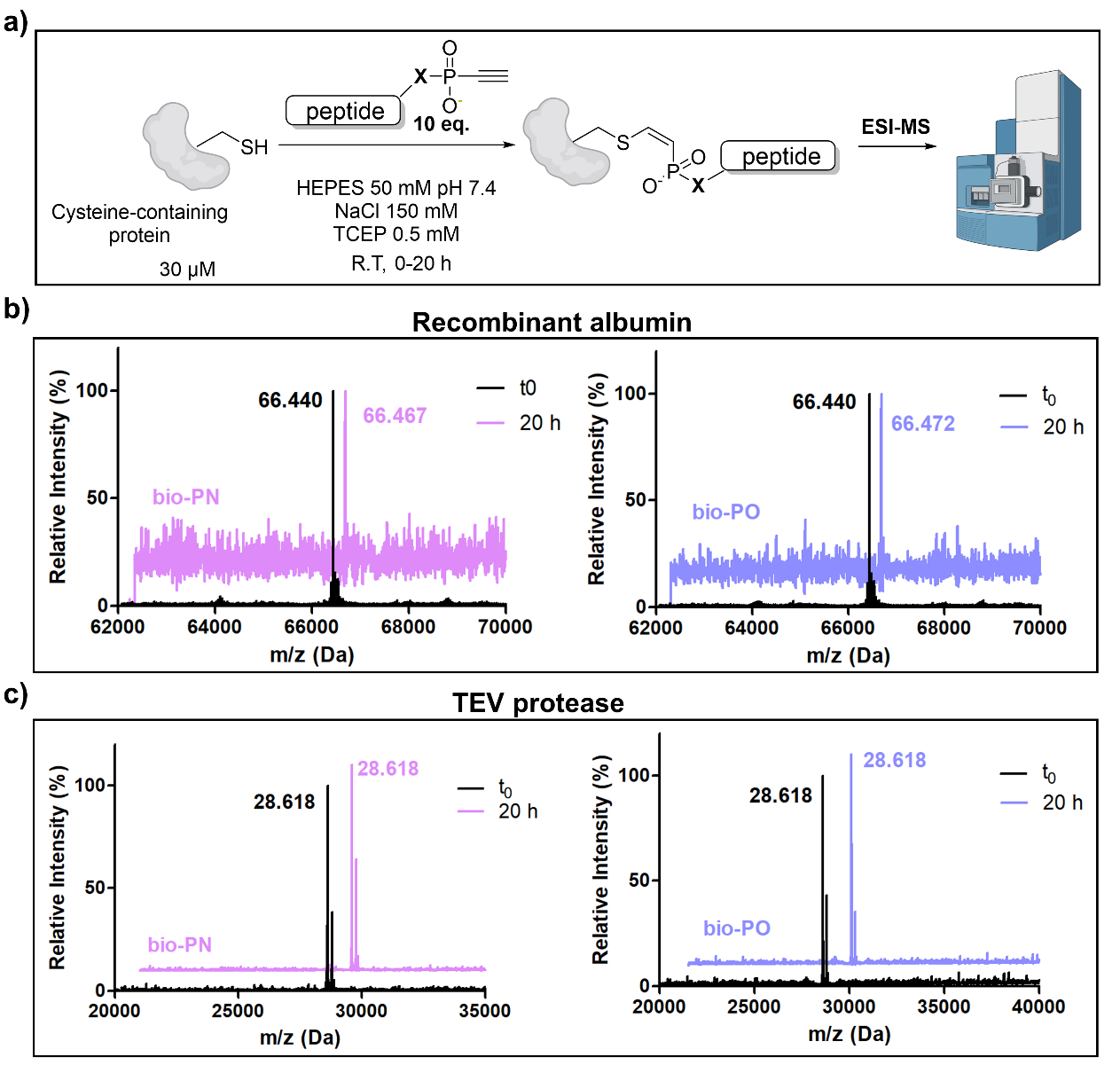
**

**Figure S5:** a) Workflow for labeling of cysteine containing proteins other than PTP1B follows the same conditions and read-out. b) Stacked deconvoluted MS spectra of the reaction between recombinant albumin and **bio-PN** (left) or **bio-PO** (right) at 0 and 20 h. No covalent labeling was observed. c) Stacked deconvoluted MS spectra of the reaction between TEV protease and **bio-PN** (left) or **bio-PO** (right) at 0 and 20 h. Similarly, no covalent labeling was detected.

**
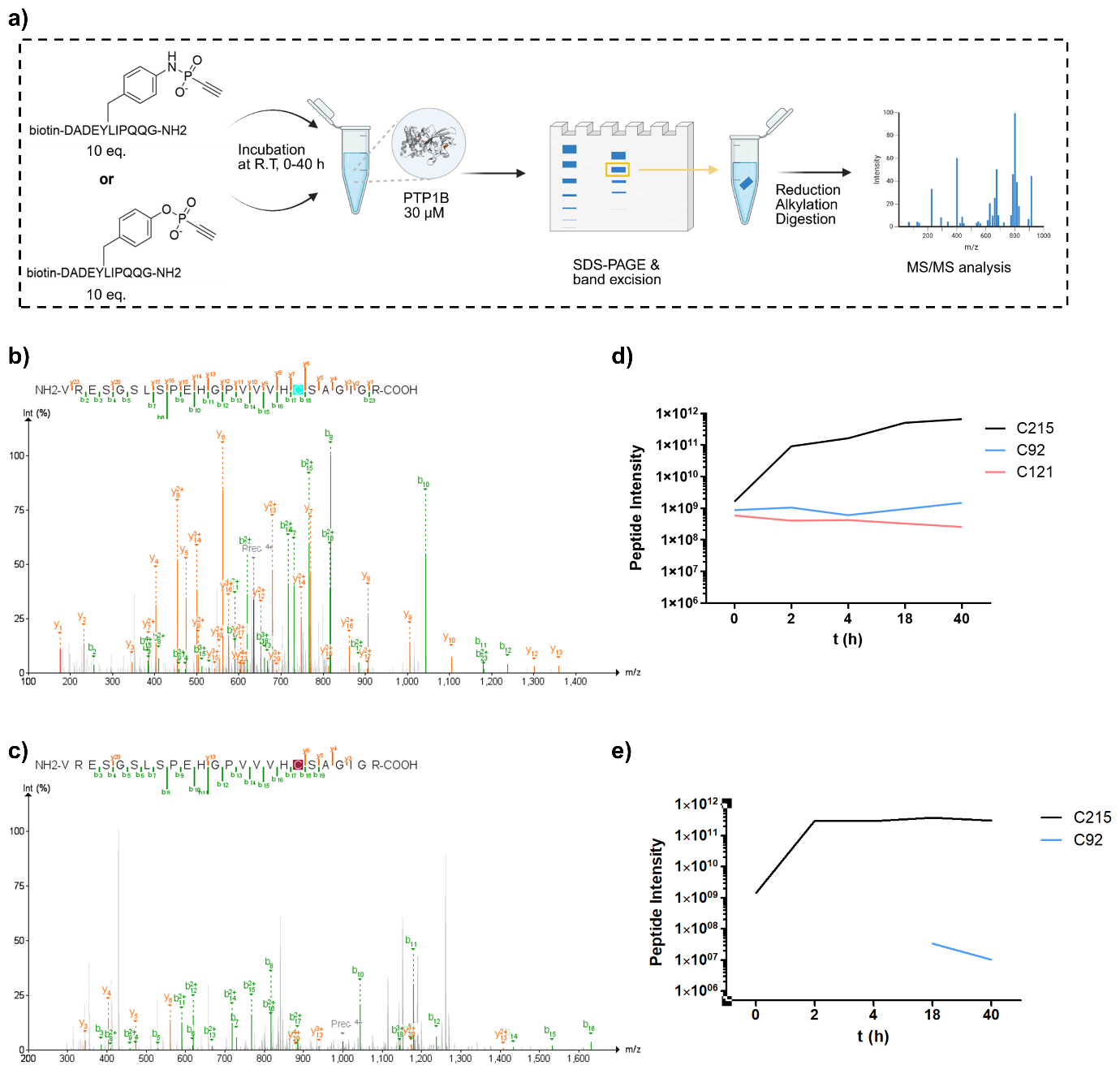
**

**Figure S6:** a) Schematic overview of the workflow to identify the site of labeling in PTP1B by **bio-PN** and **bio-PO**. MS/MS spectrum of the labeled peptide containing the modified catalytic cysteine (C215) b) with **bio-PN** and c) with **bio-PO**. Quantification graph of the intensity of the corresponding modified peptide showing the abundance of VRESGSLSPEHGPVVVHC(PV)SAGIGR (marked in black) d) in **bio-PN** sample and e) in **bio-PO** sample.


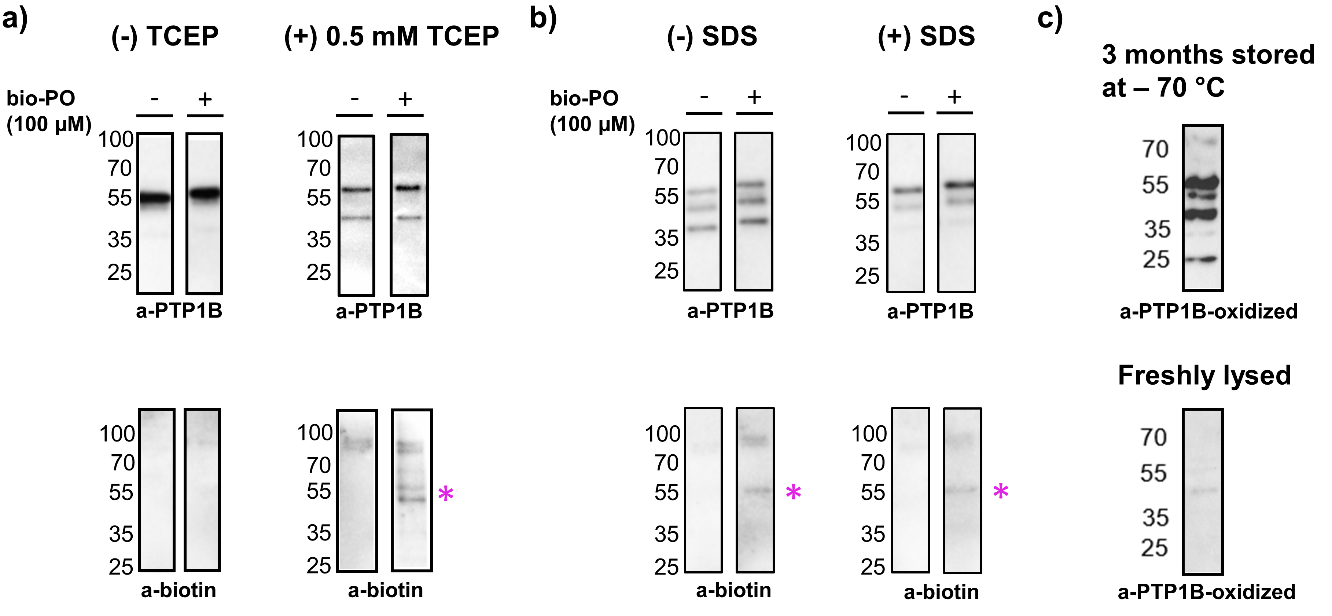


**Figure S7:** Western blot analysis of conditions where active and soluble PTP1B is present on HEK293T cell lysate (2 mg/mL) and where a biotin signal can be detected after treament with **bio-PO** (100 µM, 1 h., R.T). Tris buffer 50 mM, pH 7.4 and 150 mM NaCl was kept constant. a) Addition of 0.5 mM TCEP leads to a detection of biotin signal in the expected molecular weight range, while absence of it leads to no biotin signal. TCEP is crucial to keep the catalytic cysteine of PTP1B reduced. b) Buffer was supplemented with TCEP as well as detergents like SDS. No significant impact was obvious for this parameter. c) Cell lysate (50 mM Tris pH 7.4, 150 mM NaCl, 0.5 mM TCEP) stored at −70 °C for 3 months showed significant oxidation of the active site cysteine, while cell pellets lysed on the day of the labeling show no such effect. The asterisk represents the expected molecular weight range for PTP1B. Individual lanes are shown at the indicated lengths; each corresponds to an independent experiment.

Discussion

It is crucial in the context of our experiments that soluble, highly active and non-denatured PTP1B needs to be present in the lysate,^[1]^ therefore we screened important lysis parameters with Tris buffer and NaCl concentration remaining consistent. In principle, we wanted to identify conditions, in which we would observe labeling of HEK293T cell lysate at the desired molecular weight (~50 kDa) with 100 µM **bio-PO** for 1 hour by western blotting. In short, we observed bands at the expected molecular weight when the buffer was supplemented with TCEP to keep possible disulfides reduced, while addition of detergents such as low amounts of SDS (0.1%) did not have a notable impact in the labeling (Fig. S7 a, b). On the other hand, longer storage of the lysate (<1 month) even at −70 ºC, had a detrimental effect to enzyme activity as the catalytic cysteine was highly oxidized (Fig. S7c).^[2]^ In light of these observations, the preferred lysis buffer was Tris buffer 50 mM pH 7.4, 150 Mm NaCl, 0.5 mM TCEP and the lysate was always prepared freshly on the day of labeling form harvested cell pellets.

**Figure S8:** Comparison of labeling (1 h., R.T.) in HEK293T cell lysate (2 mg/mL) between a) the target specific probe **TMR-PO** and b) a general phosphatase probe **TMR-BBP** by fluorescence gel scanning. A more specific pattern is observed when the lysate is treated with 100 µM **TMR-PO**. Labeling with 1 mM (as was previously published ^[3]^) TMR-BBP shows a global proteome labeling as expected. Individual lanes are shown at the indicated length; each corresponds to an independent experiment.


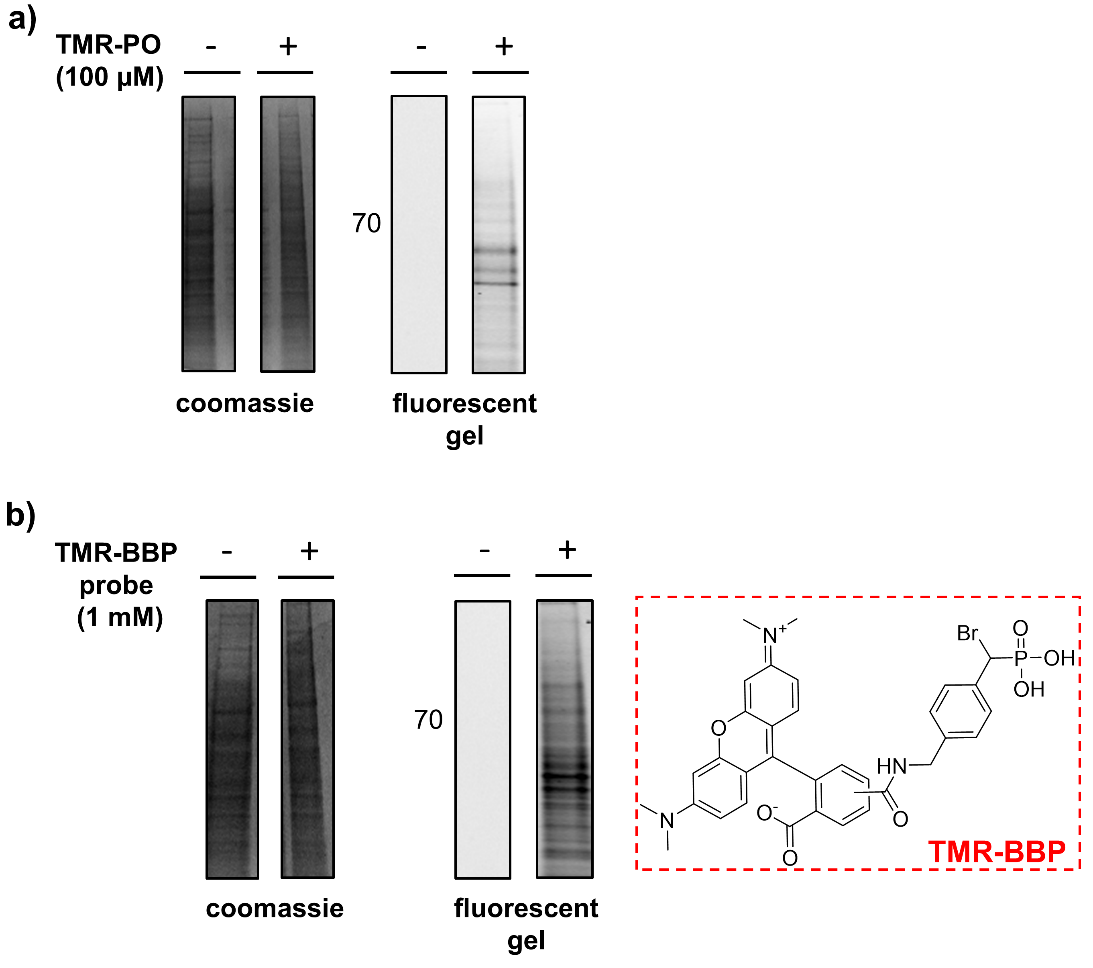


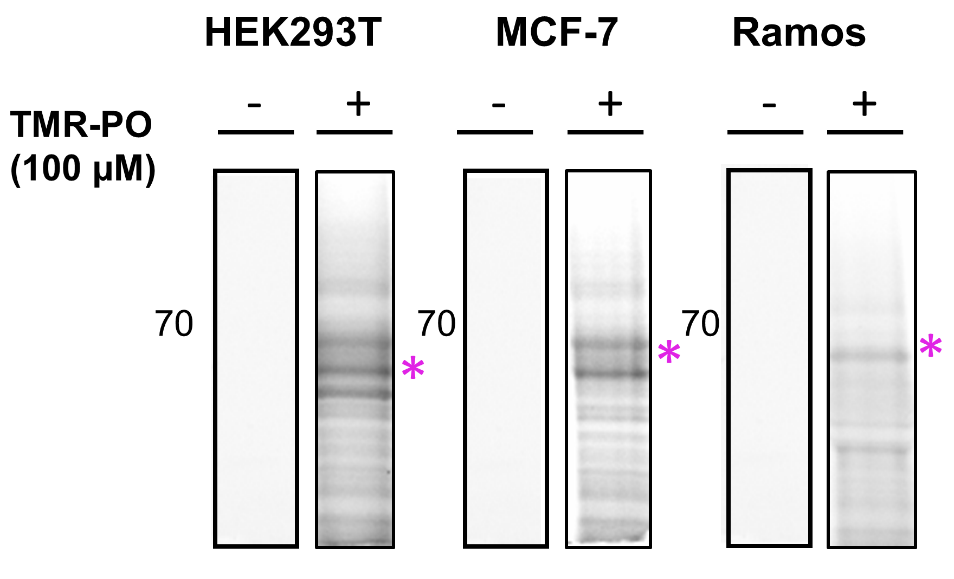


**Figure S9:** Labeling with **TMR-PO** (100 µM, 1h., R.T) across three different PTP1B-containing mammalian cell lysates (4 mg/mL). Similar distinct bands can be observed in all three experiments.The asterisk represents the expected molecular weight range for PTP1B. Individual lanes are shown at the indicated length; each corresponds to an independent experiment.


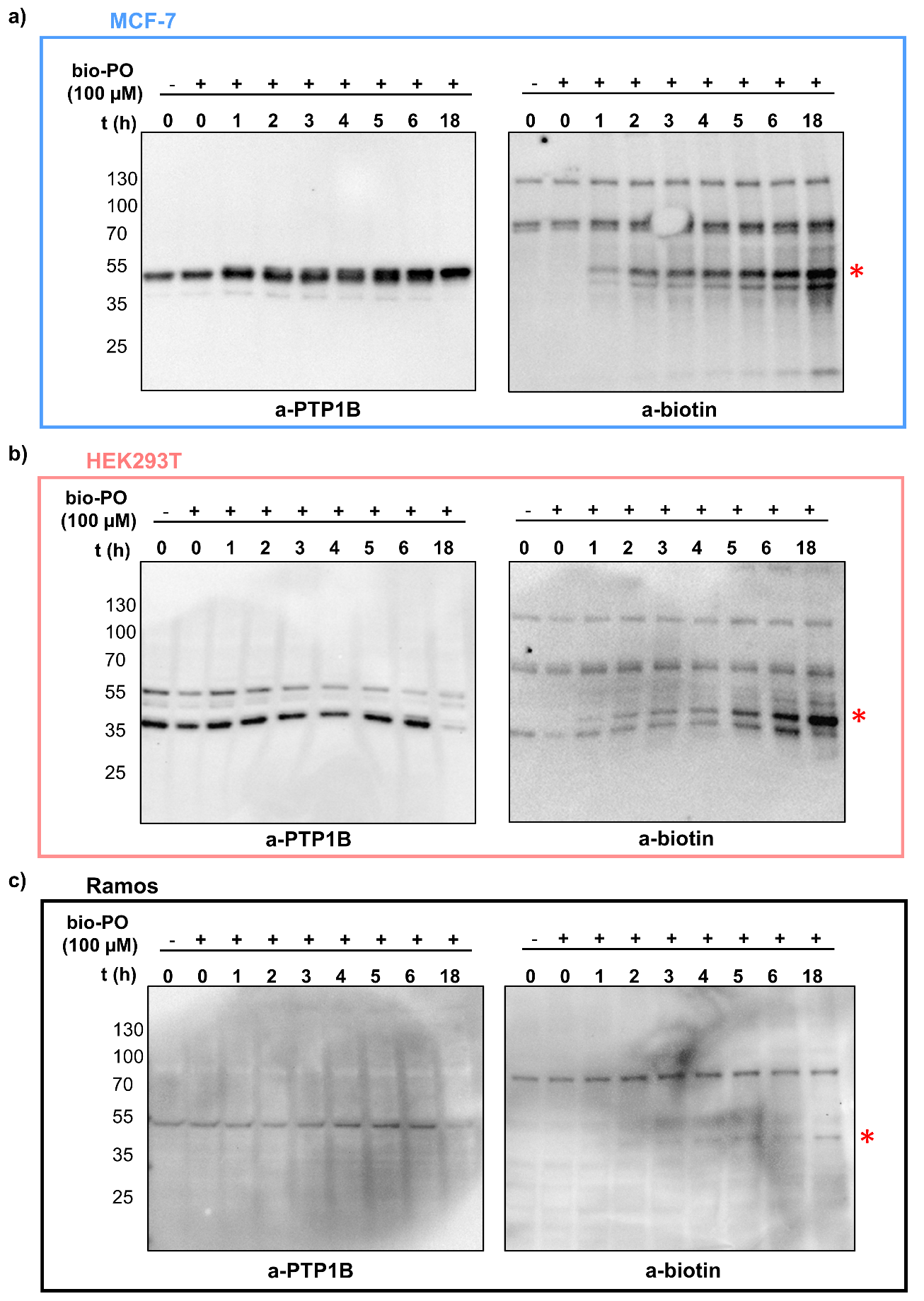


**Figure S10:** Time course labeling of lysate (4 mg/mL) with 100 µM **bio-PO**. a) Labeling in MCF-7 cell lysate b) HEK293T cell lysate c) Ramos lysate. Increasing biotin signal is observed with increasing incubation for all different lysates. Despite prolonged incubation times, labeling remains specific in all three experiments. MCF-7 lysate displays the most intense biotin signal followed by HEK293T. Biotin signal intensity is too low for Ramos lysate, which is in accordance with the low PTP1B signal as well. The asterisk represents the expected molecular weight range for PTP1B.


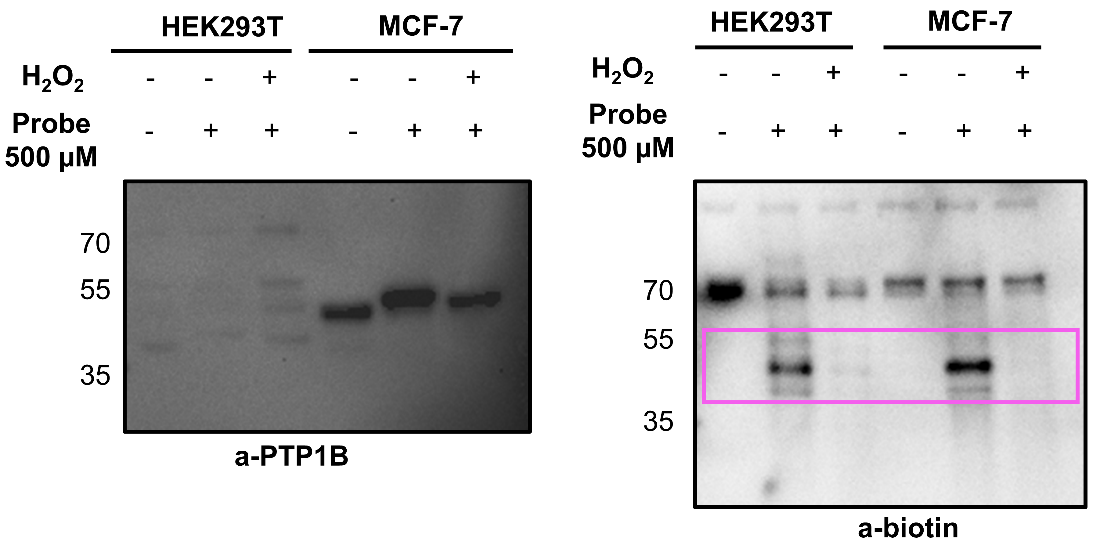


**Figure S11:** Labeling of HEK293T and MCF-7 (4 mg/mL) native and H_2_O_2_-treated lysate with **bio-PO** (400 µM) at room temperature for 4 h (visualization by western blotting left: PTP1B signal; right: biotin signal). Labeling is present at the only probe treated lanes but abolished upon treatment with H_2_O_2_ as the catalytic cysteine of PTP1B is oxidized.


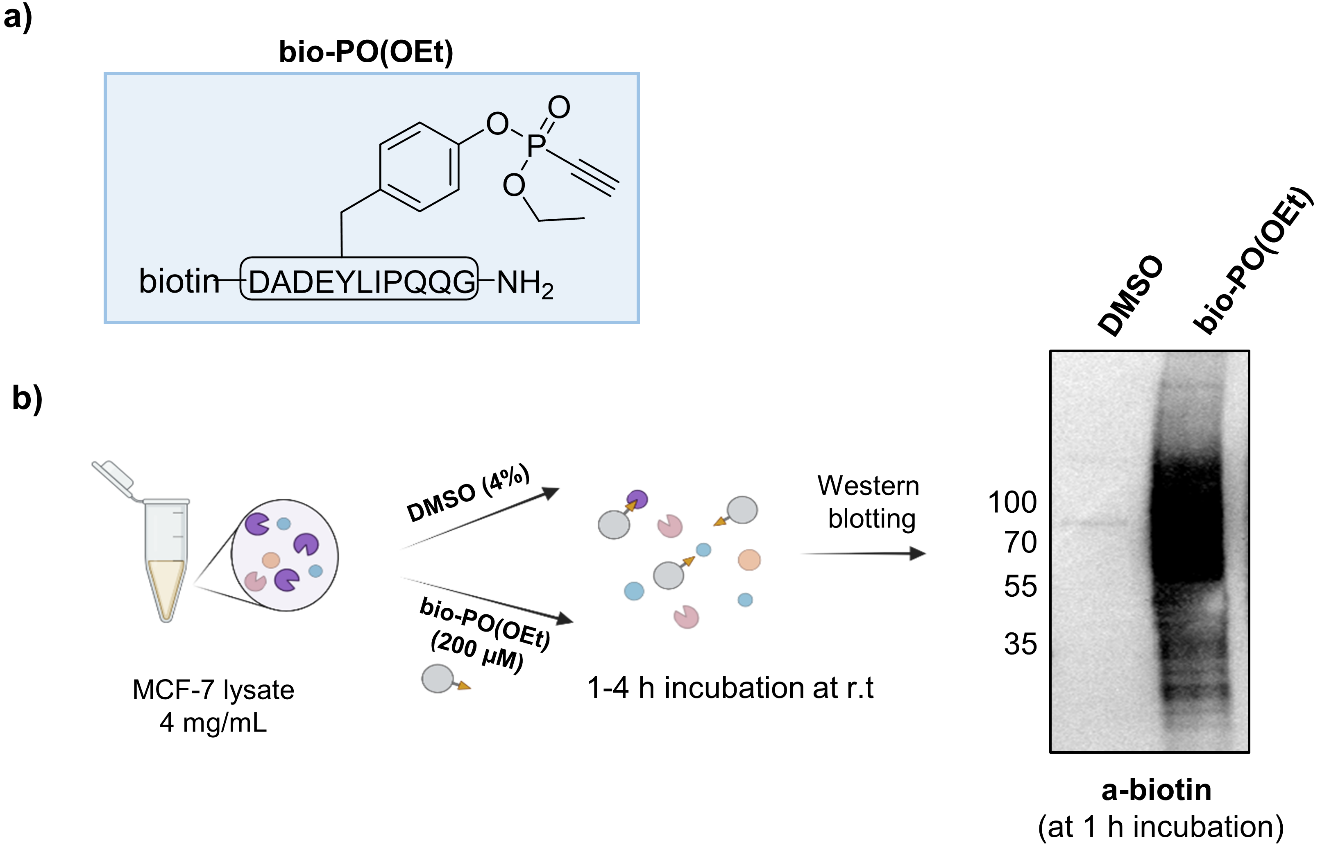


**Figure S12:** a) Structure of the more reactive O-ethyl substituted ethynyl phosphonate (**bio-PO(OEt)**) peptide used as a highly electrophilic control. b) Workflow of incubation of the peptide (200 µM) with 4 mg/mL MCF-7 lysate. A saturated biotin signal is already observed after 1 hour of incubation, emphasizing the importance of lacking –OR substitution for achieving selective labeling.


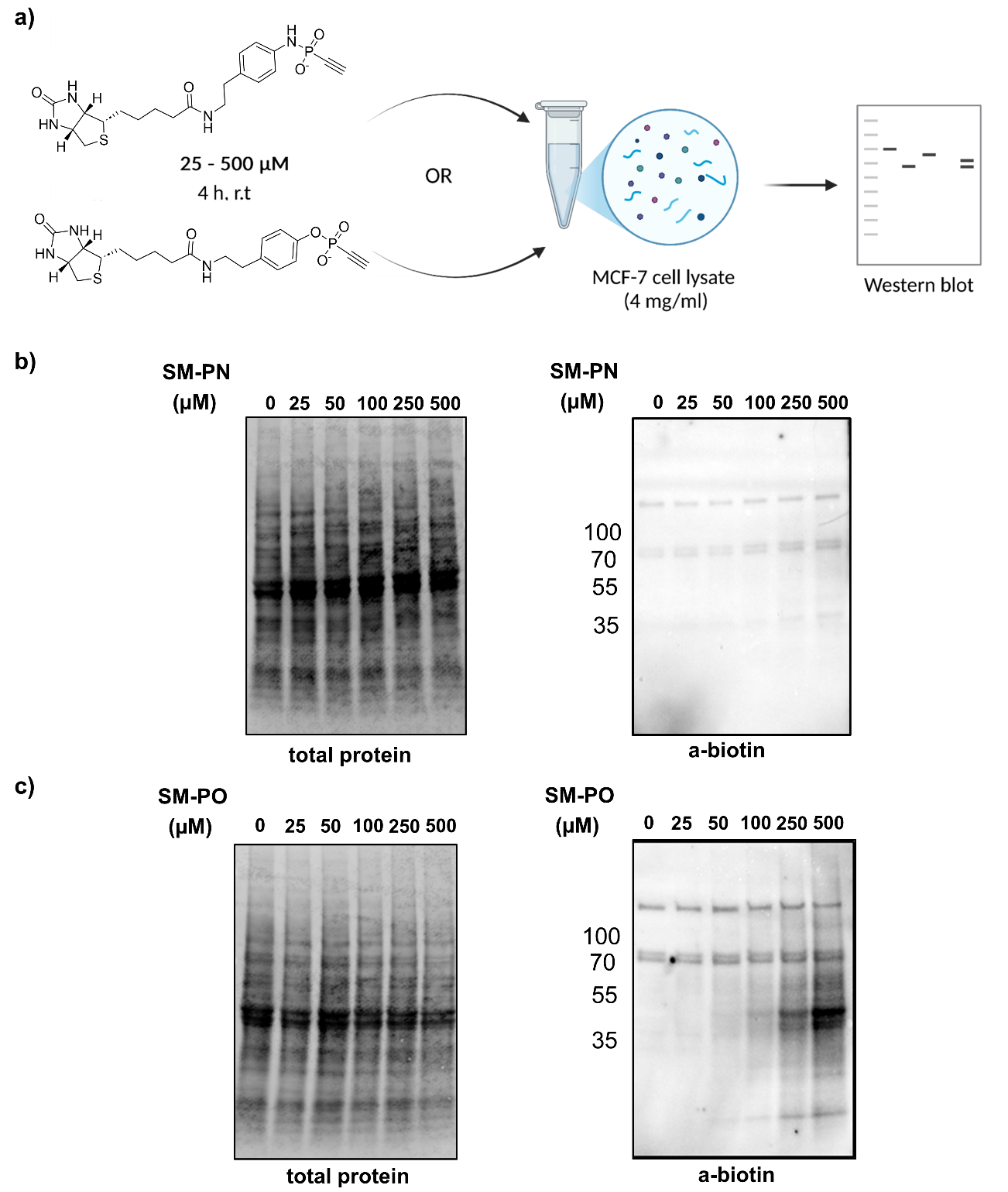


**Figure S13:** a) Overview of the workflow for labeling MCF-7 lysate (4 mg/mL) with various concentrations of **SM-PN** or **SM-PO**. b) No specific signal was observed, as only bands also present in the DMSO control (no probe) were detected with **SM-PN** treatment. c) A higher biotin signal upon concentration increase was observed for **SM-PO**, which overlapped with the most prominent band in the protein stain signal, eluding towards unspecific interactions.


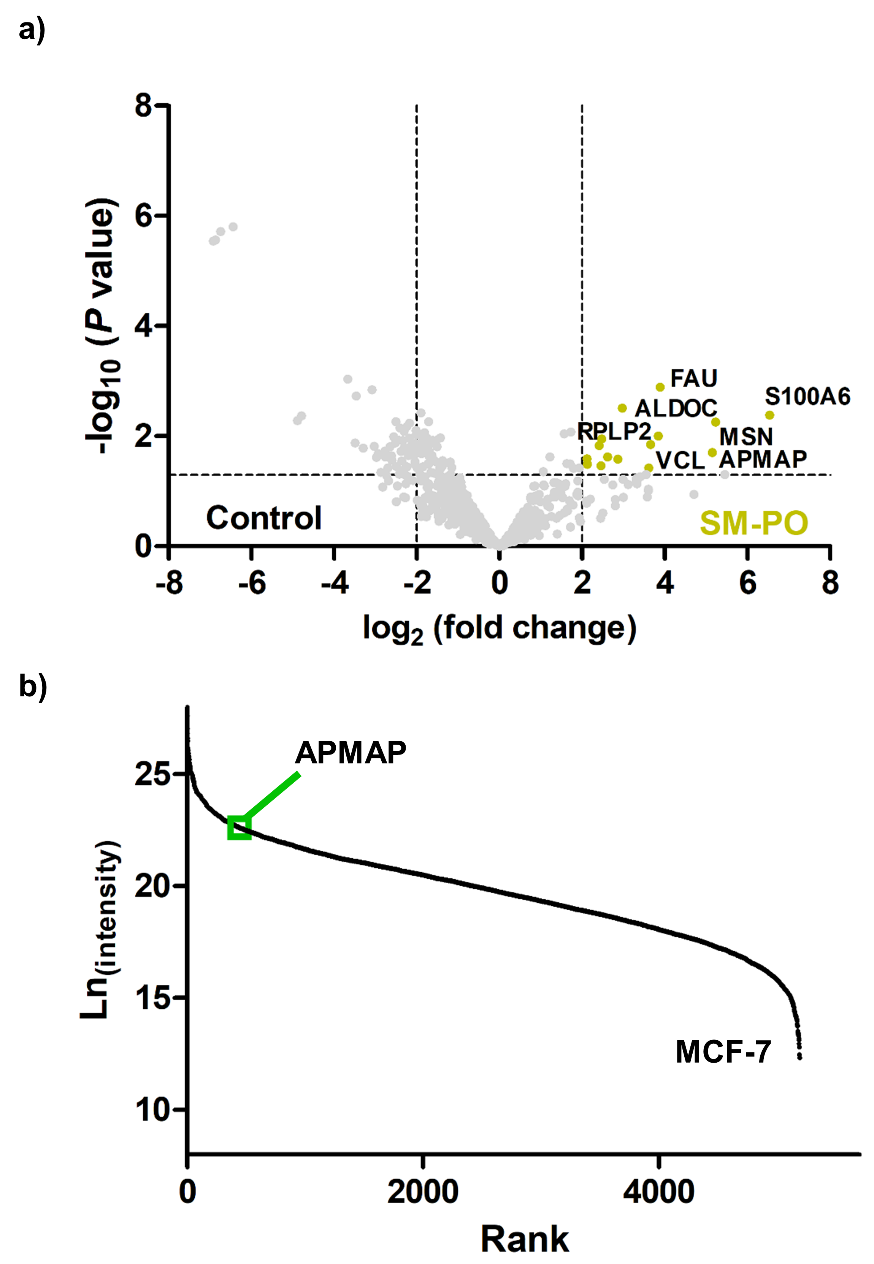


**Figure S14:** a) Volcano plot of proteomic profiling of MCF-7 lysate with **SM-PO** (right side) and DMSO (left side) as a control (enrichment >2 log_2_ fold change, p ≤ 0.05). No enrichment of PTP1B or any relevant phosphatase was observed b) Abundance of adipocyte plasma membrane–associated protein (APMAP) in MCF-7 lysate.

# **General Information**

## **Chemicals and solvents**

Chemicals and solvents were purchased from Sigma-Aldrich (Merck Group, Germany), TCI (Tokyo chemical industry CO., LTD., Japan) and Acros Organics (Thermo Fisher scientific, USA), BLD Pharm (BLD Pharmatech, China), MedChemExpress (MedChemExpress LLC, Monmouth Junction, NJ, USA) and used without further purification. Dry solvents were purchased from Acros Organics (Thermo Fisher scientific, USA). Aminoacids, coupling reagents and resins for SPPS were purchased from Novabiochem (Merck, USA) or Iris Biotech GmbH (Germany).

## **Flash- and thin layer chromatography**

Flash column chromatography was performed, using NORMASIL 60® silica gel 40–63 μm (VWR international, USA). Analytical thin layer chromatography (TLC) was performed on aluminum foil pre-coated with SiO_2_–60 F254 (Macherey-Nagel, DE). Spots were visualized by fluorescence depletion with a 254nm lamp or manganese staining (10 g K_2_CO_3_, 1.5 g KMnO_4_, 0.1 g NaOH in 100 mL H_2_O), followed by heating.

## **Semi-reparative HPLC**

Semi-preparative HPLC was performed on a Gilson PLC 2020 system (Gilson Inc, WI, Middleton, USA) using a VP 250/32 Macherey-Nagel Nucleodur C18 HTec Spum column (Macherey-Nagel, DE) and on a Shimadzu prominence HPLC system (Shimadzu Corp., Japan) with a CBM20A communication bus module, a FRC–10A fraction collector, 2 pumps LC–20AP, and a SPD-20A UV/VIS detector, using a VP250/21 Macherey-Nagel Nucleodur C18 HTec Spum column (Macherey-Nagel GmbH & Co. Kg, Germany).

## **NMR spectroscopy**

NMR spectra were recorded with a Bruker AV-III 300 MHz spectrometer and a Bruker AV-III 600 MHz spectrometer, both equipped with a broadband probe (BBFO). (CDCl₃: 7.26 ppm; DMSO-d₆: 2.50 ppm; MeOD-d₄: 3.31 ppm for ^1^H-spectra and CDCl₃: 77.16 ppm; DMSO-d₆: 39.52 ppm; MeOD-d₄: 49.00 ppm for ^13^C-spectra). Coupling constants J are stated in Hz.

## **Cell culture**

MCF-7, RAMOS and HEK293T were purchased from DSMZ (German Collection of Microorganisms and Cell Cultures GmbH) and cultured at 37 °C under a humidified 5% CO2 atmosphere with RPMI 1640 medium (BioWest) supplemented with 10% FCS (MCF-7, RAMOS) or DMEM High Glucose medium (BioWest) supplemented with 10% FCS (HEK293T). Cells were used maximum until passage 20.

## **Protein concentration determination**

Protein concentrations were determined by BCA assay (Thermo Fisher Scientific, USW) according to the manufacturer`s protocol.

## **Size-exclusion chromatography**

Protein purification by size-exclusion chromatography was conducted with an ÄKTA FPLC system (GE Healthcare, United States) equipped with a P-920 pump system, a UPC-900 detector, a FRAC-950 fraction collector and a 5 mL Superdex 75 10/300 GL column (GE Healthcare, USA) with a flow of 0.8 mL/min.

## **UPLC-UV/MS**

Samples were mostly recorded on a Waters H-class instrument equipped with a quaternary solvent manager, a Waters autosampler, a Waters TUV detector and a Waters Acquity QDa detector with an Acquity UPLC BEH C18 1.7 μm, 2.1 x 50 mm RP column with a flow rate of 0.6 mL/min (Waters Corp., USA). The following gradients were used: A = H2O + 0.1% TFA in; B = MeCN + 0.1% TFA. Long gradient 1: 5% B 0 -1.5 min, 5- 95% B 1.5-13.0 min, 95% B 13.0-13.9 min, 5% B 13.9-15 min. Long gradient 2: 3% B 0 -1.5 min, 3-60% B 1.5-13.0 min, 60% B 13.0-13.9 min, 5% B 13.9-15 min. Short gradient 1: 5% B 0 - 0.5 min, 5-95% B 0.5-3.0 min, 95% B 3.0-3.9 min, 5% B 3.9-5 min. Short gradient 2: 3% B 0 - 0.5 min, 3-60% B 0.5-3.0 min, 95% B 3.0-3.9 min, 3% B 3.9-5 min. In some cases samples were recorded on an Agilent instrument, equipped with 1290 High-speed pump (GG7120A), 1290 Multisampler (G7167B), 1260 DAD-HS (G7117C), 1290 MCT (G7116B) equipped with a Zorbax SB-C18 2.1x50 mm 1.8-micorn column and InfinityLab LC/MSD XT single quadruple mass spectrometer. Short gradient 3: A 5% B 0.0 - 0.5 min, 5-95% B 0.5 - 3.0 min, 95% B 3.0 - 4.5 min, 95-5% 4.5 - 5.0 min.

**Peptide purity (section 3.2) was determined either with long gradient 1 or 2. UPLC gradients were based on inherited methods and were not systematically optimized for each peptide, but all runs ensured adequate separation for purity assessment.*

## **HR-MS**

High resolution ESI-MS spectra were recorded on a Waters H-class instrument equipped with a quaternary solvent manager, a Waters sample manager-FTN, a Waters PDA detector and a Waters column manager with an Acquity UPLC protein BEH C18 column (1.7 μm, 2.1 mm x 50 mm). Samples were eluted with a flow rate of 0.3 mL/min. The following gradient was used: “QTof”: 0.01% FA in H_2_O; B: 0.01% FA in MeCN. 5 % B: 0-1 min; 5 to 95 % B: 1-7min; 95 % B: 7 to 8.5 min. Mass analysis was conducted with a Waters XEVO G2-XS QTof analyzer.

## **Intact protein MS**

Intact proteins were analyzed using a Waters H-class instrument equipped with a quaternary solvent manager, a Waters sample manager-FTN, a Waters PDA detector and a Waters column manager with an Acquity UPLC protein BEH C4 column (300 Å, 1.7 μm, 2.1 mm x 50 mm). Proteins were eluted with a flow rate of 0.3 mL/min with 80 °C or 40 °C column temperature. The following gradient was used: A: 0.01 % FA in H_2_O; B: 0.01 % FA in MeCN. 5-95 % B 0-6 min. Mass analysis was conducted with a Waters XEVO G2-XS QTof analyzer. Raw data was deconvoluted with MaxEnt 1.

# **Experimental procedures and characterization data**

## **Protein expression and purification**

**Tobacco Etch Virus nuclear-inclusion-a endopeptidase (TEV) Protease**

Plasmid MBP-H6-TEV S219V R5 (addgene pRK793) encoding TEV protease was transformed into *E. coli* BL21 (DE3). 10 mL starter cultures were prepared in LB (Luria- Bertani) medium with 50 µg/mL carbenicillin and grown at 37°C overnight. The next day, a 1 L LB medium culture with carbenicillin was inoculated with 10 mL of the starter culture and incubated at 37°C to an OD_600_ of 0.6-0.8, and expression was induced by the addition of 1 mM IPTG. Induction proceeded for ∼18 hours at 18°C. The cells were harvested by centrifugation for 15 minutes at 4000 x g and at 4 ˚C. Cell pellets were stored at −20°C. For the protein purification, cell pellets were lysed in TEV buffer (100 mM Na-Phosphate, 200 mM NaCl, 10% Glycerol, pH 7.4) using ultra-sonic (Branson sonifier, 6 minutes, 1 sec. on, 1 off), 25% intensity). The lysate was clarified by centrifugation at 50.000 x g (15 min, 4 °C) and was supplemented with 1 mM **dithiothreitol** (DTT). The lysate was then loaded on Ni-NTA indigo beads pre-equilibrated with lysis buffer. The bound protein was washed with Buffer A (TEV buffer supplemented with 20 mM imidazole) and eluted with Buffer B (TEV buffer with 500 mM imidazole). Elution fractions were pooled and dialyzed overnight against dialysis buffer (TEV buffer) with 1:5 (v/v) TEV protease. After purification, protein aliquots were shock-frozen and stored at −70°C.

**Protein Tyrosine Phosphatase 1B (PTP1B) WT & alanine mutant (PTP1B-C15A)**

The coding sequence of human PTP1B (residues 1–321) was PCR-amplified using a plasmid encoding residues 1–301 (Addgene #102719) as a template.^[4]^ The coding region was extended to residue 321 by site directed mutagenesis using the QuickChange kit and cloned into **pET–28a(+)** downstream from His-tag and TEV cleavage site by restriction cloning using NheI and XhoI restriction sites. The resulting plasmid was transformed into E. coli DH5α for plasmid amplification and verified by sequencing. The **C215A** mutant was generated by site-directed mutagenesis as well. The amino acid sequence of the expressed constructs is provided below. For protein expression the plasmids were transformed into *E. coli* BL21 (DE3). 5 mL starter cultures were prepared in LB (Luria–Bertani) medium with 50 µg/mL Kanamycin and grown at 37°C overnight. The next day, a 500 mL LB medium culture with kanamycin was inoculated with 2 mL of the starter culture and incubated at 37°C to an OD_600_ of ∼0.6, and expression was induced by the addition of 1 mM IPTG. Induction proceeded for ∼20 hours at 18°C. The cells were harvested by centrifugation for 15 minutes at 4000 x g. Cell pellets were stored at −20°C. For the protein purification, cell pellets were lysed in lysis buffer (25 mM Tris pH 8.0, 500 mM NaCl, 5 mM imidazole, 0.1% Triton X-100) using ultra-sonic (Branson sonifier, 3 x 2 minutes, 30% intensity). The lysate was clarified by centrifugation at 25000 x g). The lysate was then loaded on Ni-NTA beads pre-equilibrated with lysis buffer. The bound protein was then washed with Buffer A (50 mM Tris pH 8.0, 500 mM NaCl, 20 mM imidazole) and eluted with Buffer B (50 mM Tris pH 8.0, 500 mM NaCl, 250 mM imidazole). The protein was then further purified using Size Exclusion Chromatography (SEC, Superdex 75 16/60) into assay buffer (50 mM HEPES, pH 7.4, 150 mM NaCl, 0.5 mM TCEP). After purification, protein aliquots were shock-frozen and stored at –70°C.

**PTP1B WT- amino acid sequence**

GSDKIHHHHHHENLYFQGHMASMEMEKEFEQIDKSGSWAAIYQDIRHEASDFPCRVAKLPKNKNRNRYRDVSPFDHSRIKLHQEDNDYINASLIKMEEAQRSYILTQGPLPNTCGHFWEMVWEQKSRGVVMLNRVMEKGSLKCAQYWPQKEEKEMIFEDTNLKLTLISEDIKSYYTVRQLELENLTTQETREILHFHYTTWPDFGVPESPASFLNFLFKV RESGSLSPEH GPVVVHCSAGIGRSGTFCLA DTCLLLMDKR KDPSSVDIKK VLLEMRKFRM GLIQTADQLR FSYLAVIEGAKFIMGDSSVQ DQWKELSHED LEPHN

**
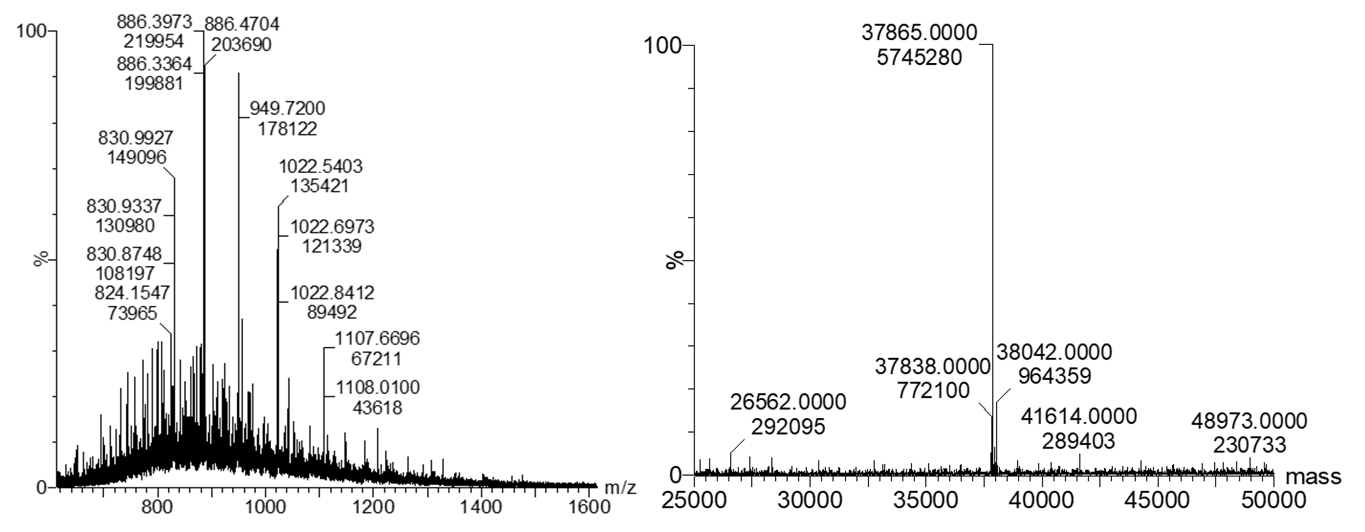
**

Non-deconvoluted (left) and deconvoluted (right) spectrum of WT PTP1B after SEC purification.

**PTP1B-C215A - amino acid sequence**

GSDKIHHHHHHENLYFQGHMASMEMEKEFEQIDKSGSWAAIYQDIRHEASDFPCRVAKLPKNKNRNRYRDVSPFDHSRIKLHQEDNDYINASLIKMEEAQRSYILTQGPLPNTCGHFWEMVWEQKSRGVVMLNRVMEKGSLKCAQYWPQKEEKEMIFEDTNLKLTLISEDIKSYYTVRQLELENLTTQETREILHFHYTTWPDFGVPESPASFLNFLFKVRESGSLSPEHGPVVVHASAGIGRSGTFCLADTCLLLMDKRKDPSSVDIKKVLLEMRKFRMGLIQTADQLRFSYLAVIEGAKFIMGDSSVQ DQWKELSHED LEPHN

**
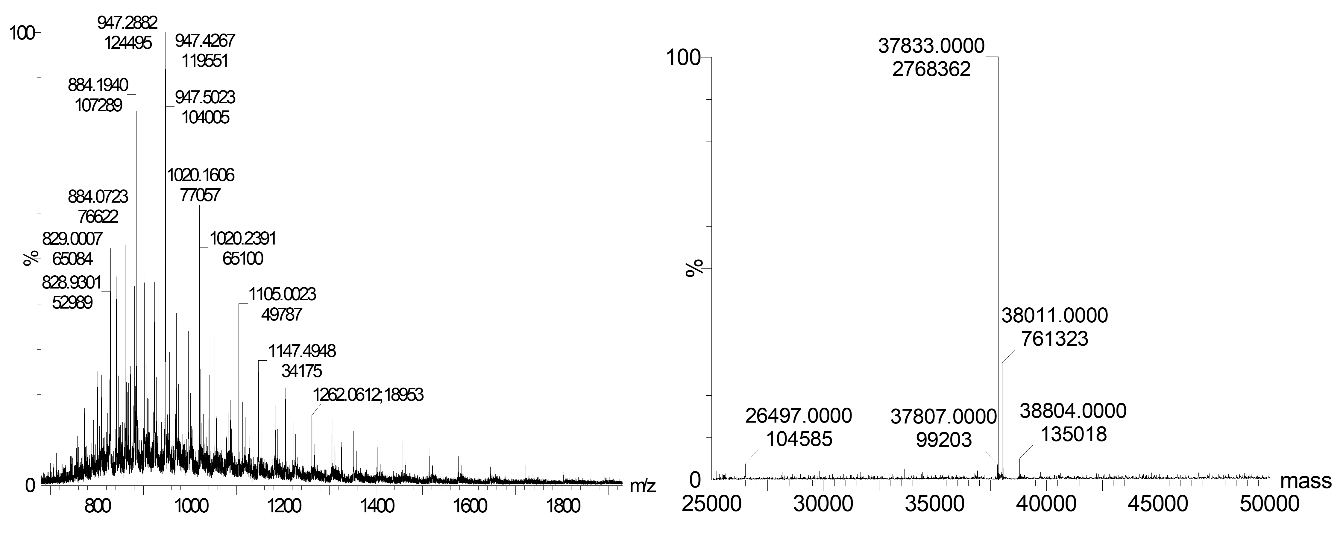
**

Non-deconvoluted (left) and deconvoluted (right) spectrum of PTP1B-C215A after SEC purification.

## **Peptide synthesis**

### **Procedure for phosphonamidate-based peptides**

#### **Synthesis and purification of azido containing precursor peptides**

**General procedure**

Peptides with the sequence DADEF(N_3_)LIPQQG-NH_2_ were synthesized by Fluorenylmethoxycarbonyl (Fmoc)-solid-phase peptide synthesis (SPPS) using a peptide synthesizer (Automated microwave peptide synthesizer, CEM) on Fmoc-Rink-Amide aminomethyl (Fmoc-RA-AM) polystyrene resin (Iris Biotech, cat. No. **BR-1320, 0.4 mmol/g, 0.05-0.1 mmol scale**). Couplings were achieved by reacting 0.2 M Fmoc-AA-OH with 0.25 M DIC and 0.25 M Oxyma in DMF. A solution of 20% piperidine in DMF was used to remove the Fmoc protection group. Afterwards, biotin (Iris Biotech, cat. No. **LS-1070), 5(6)-tetramethylrhodamine (TAMRA) (MedChemExpress, cat. No.** HY-15944) or pentynoic acid (Sigma- Aldrich, cat. No 232211) were coupled manually at the C-terminus using 0.2 M solutions with 0.2 M HATU and 0.4 M DIPEA, incubated for 2 hours at room temperature. Crude peptides were washed five times with dry diethyl ether. Final deprotection was done by adding 95:2.5:2.5 of TFA/TIS/H_2_O for 2-3 hours at 25 ˚C. Peptides were purified by preparative reverse phase-high performance liquid chromatography (RP-HPLC) on using gradient: A = H_2_O +0.1% TFA, B = MeCN + 0.1% TFA, 0-50 min, 20-80% B in A, flowrate: 10 mL/min). Products were obtained as a white powder after lyophilization and analyzed by UPLC-UV and HR-MS.

Peptide **bio-N3**: biotin-DADEF(N_3_)LIPPQG-NH_2_

Isolated yield: 36 mg (24 µmol, 47 %) starting from 50 µmol scale.

**HR-MS (ESI)** *m*/*z* calcd. for C_64_H_95_N_19_O_21_S^2+^:749.8411 [M+2H^+^]; found: 749.8483.


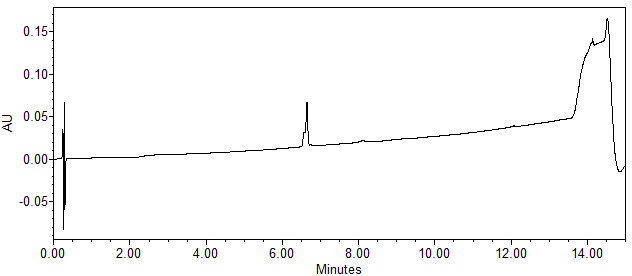


Peptide **TMR-N3**: 5/6 TAMRA-DADEF(N_3_)LIPPQG-NH_2_

Isolated yield: 11 mg (6.5 µmol, 26 %) starting from 25 µmol scale.

**HR-MS (ESI)** *m*/*z* calcd. for C_79_H_102_N_19_O_23_^2+^:843.3770 [M+2H^+^]; found: 843.3748.

**
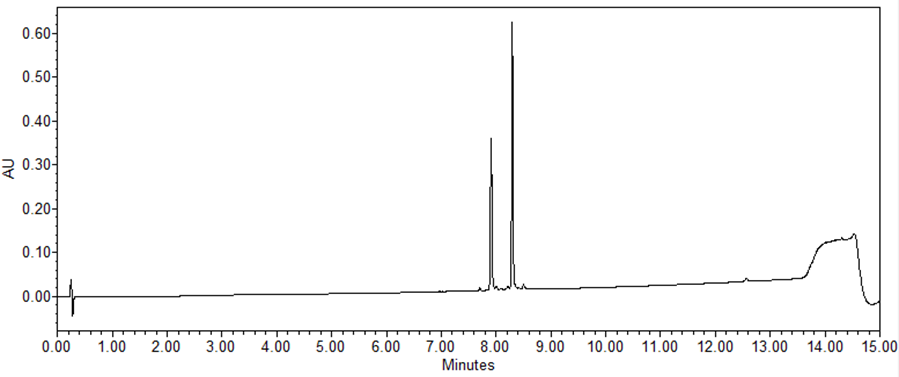
**

Peptide **penty-N3**: pentynoic-DADEF(N_3_)LIPPQG-NH_2_

Isolated yield: 26 mg (19 µmol, 76 %) starting from 25 µmol scale.

**HR-MS (ESI)** *m*/*z* calcd. for C_59_H_85_N_17_O_20_^2+^:676.8154 [M+2H^+^]; found: 676.8147.

**
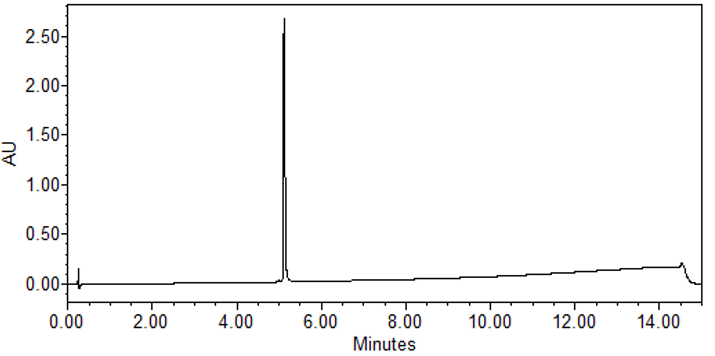
**

#### **Synthesis and purification of intermediate phosphonamidate peptides**

**General procedure**

A microcentrifuge tube (1.5 mL) was charged with azido-peptide (1.0 eq.). Ethynylphosphonite **1** was subsequently added (2.0 eq.) from a concentrated stock in dry DMF (maximum added volume 250 µL). The reaction tube was flushed with nitrogen, sealed with parafilm and stirred overnight at 750 rpm, at room temperature. The next day, the reaction was diluted with a mixture of 20% MeCN and 80% H_2_O containing 0.1% TFA and was immediately purified by HPLC (gradient: A = H2O +0.1% TFA, B = MeCN + 0.1% TFA, 0-60 min, 25-85% B in A, flowrate: 10 mL/min).

**Bio**- **phosphonamidate** peptide

The peptide was prepared according to general procedure from **bio-N3** (25 mg, 16 µmol, 1.0 eq.) and ethynylphosphonite **1** (32 µmol, 2.0 eq.; see section 3.3.1) in 215 µL dry DMF (0.15 M). Next, HPLC purification followed and the peptide was obtained as a white powder after lyophilization.

Isolated yield: 12 mg (7 µmol, 44 %) starting from 16 µmol.

**HR-MS (ESI)** *m*/*z* calcd. for C_75_H_106_N_17_O_25_PS^2+^: 854.8578 [M+2H^+^]; found: 854.8672.

**
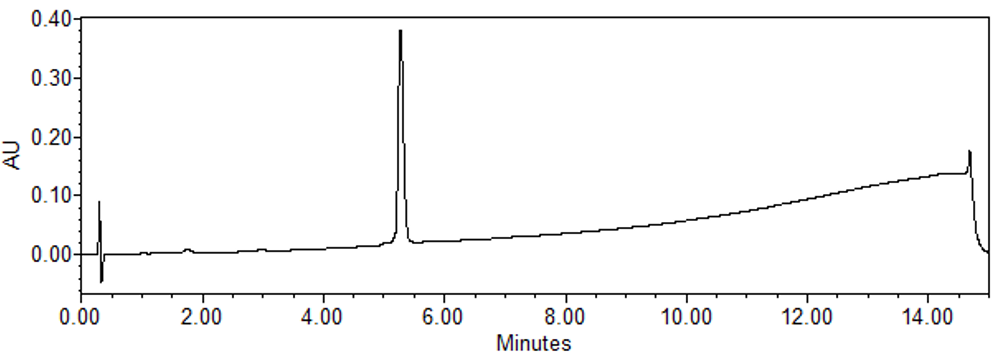
**

**5/6 TMR**- **phosphonamidate** peptide

The peptide was prepared according to general procedure from **TMR-N3** (9.3 mg, 5.5 µmol, 1.0 eq.) and ethynylphosphonite **1** (11 µmol, 2.0 eq.; see section 3.3.1) in 220 µL dry DMF (0.05 M). The isolated peptide was obtained as a pink powder after HPLC purification and lyophilization.

Isolated yield: 3 mg (1.6 µmol, 29 %) starting from 5.5 µmol.

**HR-MS (ESI)** *m*/*z* calcd. for C_90_H_113_N_17_O_27_P^2+^: 948.3937 [M+2H^+^]; found: 948.4032.


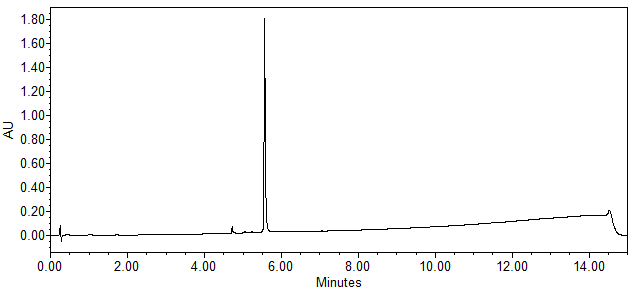


**Penty-phosphonamidate** peptide

The peptide was synthesized according to general procedure from **penty-N3** (10 mg, 7.5 µmol, 1.0 eq.) and ethynylphosphonite **1** (15 µmol, 2.0 eq.; see section 3.3.1) in 150 µL dry DMF (0.1 M). After HPLC purification and lyophilization the peptide was obtained as a white powder.

Isolated yield: 6.6 mg (4 µmol, 53 %) starting from 7.5 µmol.

**HR-MS (ESI)** *m*/*z* calcd. for C_70_H_96_N_15_O_24_P^2+^: 781.8320 [M+2H^+^]; found: 781.8311.

**
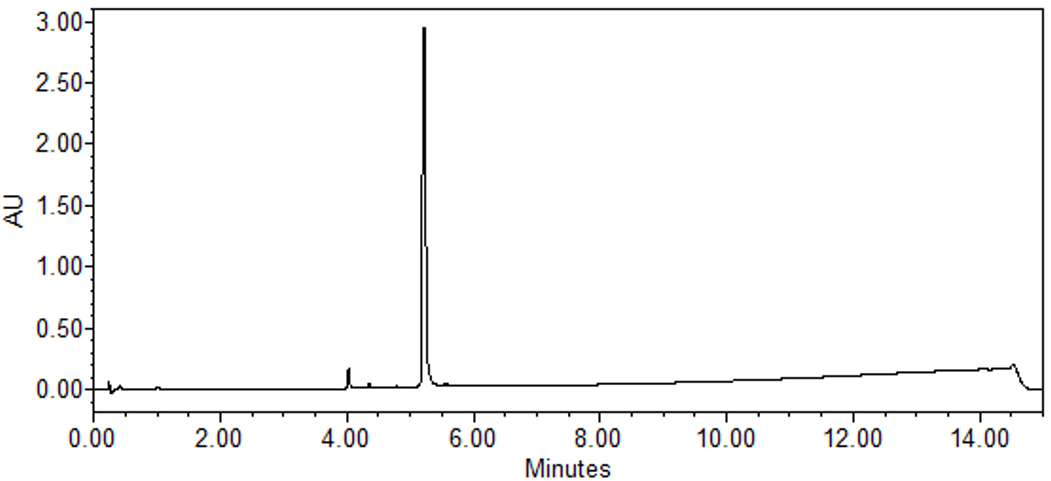
**

#### **Synthesis and purification of the final phosphonamidic acid peptides**

**General procedure**

In a microcentrifuge tube (2 mL), cleavable phosphonamidate peptide (1.0 eq.) was dissolved in 50 mM Tris buffer pH 7.4 with 20% MeCN. Equal volume of a 50 µM solution of esterase from porcine liver (lyophilized powder, Sigma-Aldrich, cat. No. E3019-3.5KU) in Tris buffer pH 7.4 was subsequently added (final peptide concentration 2.5 mM). The enzymatic reaction was stirred at 450 rpm, at room temperature for two hours, followed by spin filtration (10k MWCO) to remove the enzyme. Crude peptide was diluted with 10 mM NH_4_OAc pH 8.5 and was directly purified by semi-preparative RP-HPLC using a basic eluent system (A: 100% 10 mM NH_4_OAc pH 8.5, B: 90% MeCN + 10% A, 0-45 min, 10-80% B in A, flowrate: 5mL/min).

Peptide **bio-PN**

The peptide was prepared according to general procedure from **bio-phosphonamidate** peptide (6.5 mg, 3.8 µmol, 1.0 eq.) in total 760 µL of buffer and 25 µM esterase. The isolated peptide was obtained as a white powder after HPLC purification and lyophilization.

Isolated yield: 4.9 mg (3.2 µmol, 84 %) starting from 3.8 µmol.

**HR-MS (ESI)** *m*/*z* calcd. for C_66_H_98_N_17_O_23_PS^2+^: 780.8315 [M+2H^+^]; found: 780.8361.

**
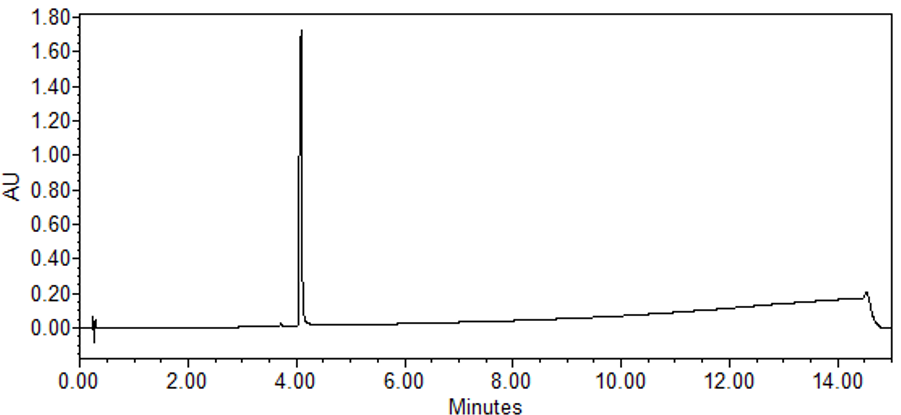
**

Peptide **TMR-PN**

The peptide was prepared according to general procedure from **TMR-phosphonamidate** peptide (3 mg, 1.6 µmol, 1.0 eq.) in total 600 µL of buffer and 25 µM esterase. The isolated peptide was obtained as a pink powder after HPLC purification and lyophilization.

Isolated yield: 2.5 mg (1.4 µmol, 87 %) starting from 1.6 µmol.

**HR-MS (ESI)** *m*/*z* calcd. for C_81_H_105_N_17_O_25_P^2+^: 874.8595 [M+2H^+^]; found: 874.8576.


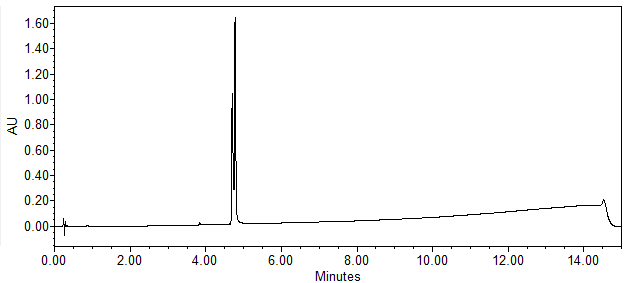


Peptide **penty-PN**

The peptide was prepared according to general procedure from **penty-phosphonamidate** peptide (6.6 mg, 4.2 µmol, 1.0 eq.) in total 840 µL of buffer and 25 µM esterase. The isolated peptide was obtained as a white powder after HPLC purification and lyophilization.

Isolated yield: 5 mg (3.5 µmol, 84 %) starting from 4.2 µmol.

**HR-MS (ESI)** *m*/*z* calcd. for C_61_H_88_N_15_O_22_P^2+^: 707.8058 [M+2H^+^]; found: 707.8044.


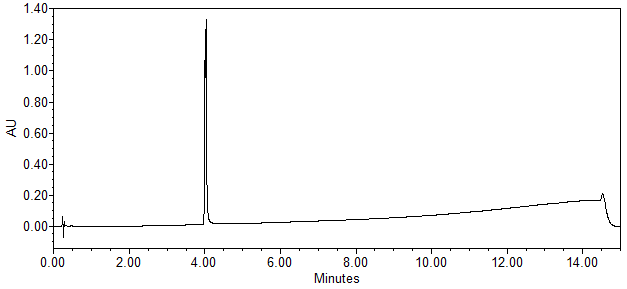


### **Procedure for phosphonate-based peptides**

#### **Synthesis and purification of phosphonic acid (PO) peptides**

**General procedure**

Peptides with the sequence DADEY(Clt)LIPQQG-NH_2_ (YClt: N_α_-Fmoc-O-2-chlorotrityl-L-tyrosine, Iris Biotech, cat. No**. FAA1235**) were synthesized by Fluorenylmethoxycarbonyl (Fmoc)-solid-phase peptide synthesis (SPPS): either manually or using an automated peptide synthesizer (room temperature synthesis) on Fmoc-Rink-Amide PEG AM resin (Iris Biotech, cat. No. BR-**1360, 0.36 mmol/g, 0.025-0.05 mmol scale**). Couplings were achieved by reacting 0.2 M Fmoc-AA-OH with 0.25 M DIC and 0.25 M Oxyma in DMF with the exception of Y(Clt) that was successfully coupled with 0.2 M HATU and 0.4 M DIPEA. A solution of 20% piperidine in DMF was used to remove the Fmoc protection group. Lastly, biotin (Iris Biotech, cat. No. **LS-1070), 5(6)-tetramethylrhodamine (TAMRA) (MedChemExpress, cat. No.** HY-15944) or pentynoic acid (Sigma- Aldrich, cat. No 232211) were coupled manually at the *C-*terminus using 0.2 M solutions with 0.2 M HATU and 0.4 M DIPEA, incubated for 2 hours at room temperature. Afterwards, the chlorotrityl group was selectively deprotected by agitating the resin with a solution of 2% TFA in CH_2_Cl_2_ for 15 minutes, repeated three times, followed by treatment with 5% TFA in CH_2_Cl_2_ for 5 minutes. Crude peptides were washed ten times with dry CH_2_Cl_2_ and five times with dry diethyl ether. Then, the dried peptide was added in a round bottom flask containing 1 mmol of crude phosphonamidite **2** or **3** (for the ethyl substituted phosphonate peptide-see section 3.3) together with 2 mL dry CH_2_Cl_2_ and stirred overnight at room temperature. The following day 2 mmol of tert-Butyl hydroperoxide (250 µL) solution 70 wt. in H₂O was added at 0 ˚C, allowed to warm up and incubated for 30-60 minutes at room temperature[.](https://www.google.com/url?sa=t&source=web&rct=j&opi=89978449&url=https://www.sigmaaldrich.com/DE/en/product/aldrich/458139%3Fsrsltid%3DAfmBOoq5w-bqlGpS-NNuu5hZ-BFEKMr5strzs_FApIxU8qvUvcwXda96&ved=2ahUKEwi5z5f4s4qOAxVjbfEDHTW5AbIQFnoECE8QAQ&usg=AOvVaw1_53jjspF5ITHIHbNdjWqS) Modified crude peptides were collected with a pipette tip, with the end cut to widen the bore, back to the reactor and washed three times with H_2_O to remove reaction salts, five times with CH_2_Cl_2_ and finally five times with dry diethyl ether. Final deprotection was done by adding 92.5:2.5:2.5:2.5 of TFA/TIS/H_2_O/EDT for 3 hours at 25 ˚C. Peptides were purified by preparative reverse phase-high performance liquid chromatography (RP-HPLC) on using gradient: A = H_2_O +0.1% TFA, B = MeCN + 0.1% TFA, 0-60 min, 20-85% B in A, flowrate: 10 mL/min). Products were obtained as a white powder after lyophilization and analyzed by UPLC-UV and HR-MS.

#### **Solid support screening for on-resin modification**

Three resins were tested for compatibility with the on-resin modification described above for the general synthesis of phosphonic acid peptides, using as exemplary model peptide **bio-PO**: A. Fmoc-Rink-Amide AM resin (Iris Biotech, cat. No. **BR-1320,** B. ChemMatrix® resin (100% PEG, originally from SEQENS/Iris Biotech; discontinued as of October 2022), C. Fmoc-Rink-Amide PEG AM resin (Iris Biotech, cat. No. BR-**1360). The ability of all resins to swell in the solvents used in the synthesis of phosphonamidite 3 and therefore allow the modification of tyrosine was assessed by following the full general procedure and performing test-cleavage on a portion of the resin with** 92.5:2.5:2.5:2.5 of TFA/TIS/H_2_O/EDT. The reaction outcome was monitored by UPLC-UV/MS. Results are summarized in figure S1 below. Fmoc-Rink-Amide PEG AM resin provided the best results and was used in all subsequent experiments.

Peptide **bio-PO**

The peptide was prepared according to general procedure from **bio-DADEY(Clt)LIPQQG-NH_2_** (25 µmol SPPS scale) and 1 mmol of crude **2**. The isolated peptide was obtained as a white powder after HPLC purification and lyophilization.

Isolated yield: 7.1 mg (4.5 µmol, 18 %) starting from 25 µmol on resin.

**HR-MS (ESI)** *m*/*z* calcd. for C_66_H_97_N_16_O_24_PS^2+^: 781.3235 [M+2H^+^]; found: 781.3278.


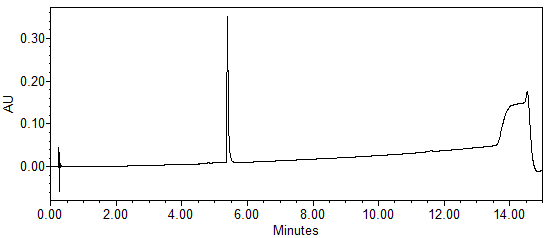


Peptide **TMR-PO**

The peptide was prepared according to general procedure from **5/6 TMR-DADEY(Clt)LIPQQG-NH_2_** (25 µmol SPPS scale) and 1 mmol of crude **2**. The isolated peptide was obtained as a light pink powder after HPLC purification and lyophilization.

Isolated yield: 5.4 mg (3.1 µmol, 13 %) starting from 25 µmol on resin.

**HR-MS (ESI)** *m*/*z* calcd. for C_81_H_104_N_16_O_26_P^2+^: 874.8595 [M+2H^+^]; found: 874.8514.


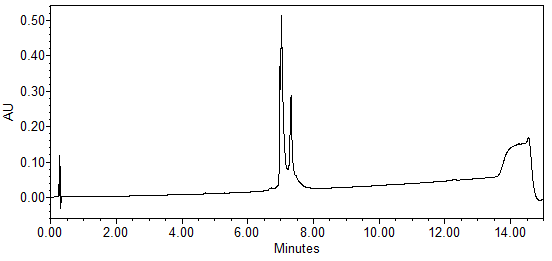


Peptide **penty-PO**

The peptide was prepared according to general procedure from **penty-DADEY(Clt)LIPQQG-NH_2_** (25 µmol SPPS scale) and 1 mmol of crude **2**. The isolated peptide was obtained as a white powder after HPLC purification and lyophilization.

Isolated yield: 4.9 mg (3.5 µmol, 14 %) starting from 25 µmol on resin.

**HR-MS (ESI)** *m*/*z* calcd. for C_61_H_87_N_14_O_23_P^2+^: 708.2978 [M+2H^+^]; found: 708.3025.


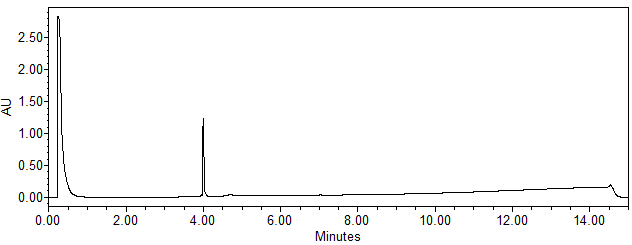


*O*-ethyl substituted phosphonate peptide – **bio-PO(OEt)**

The peptide was prepared according to general procedure from **bio-DADEY(Clt)LIPQQG-NH_2_** (12.5 µmol SPPS scale) and 1 mmol of crude phosphonamidite **3**. The isolated peptide was obtained as a white powder after HPLC purification and lyophilization.

Isolated yield: 1.6 mg (1 µmol, 8 %) starting from 12.5 µmol on resin.

**HR-MS (ESI)** *m*/*z* calcd. for C_68_H_101_N_16_O_24_PS^2+^: 795.3391 [M+2H^+^]; found: 795.3419.


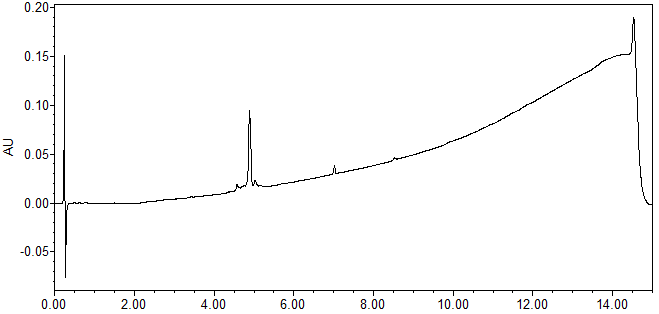


#### **Procedure for phosphotyrosine peptide (pY) and scrambled phosphotyrosine peptide (spY)**

Peptides with the sequence biotin-DADEpYLIPQQG-NH_2_ or biotin-GEDIpYPQLDQA-NH_2_ were synthesized by Fluorenylmethoxycarbonyl (Fmoc)-solid-phase peptide synthesis (SPPS) using a peptide synthesizer (Automated peptide synthesizer, PTI) on Fmoc-Rink-Amide aminomethyl (Fmoc-RA-AM) polystyrene resin (Iris Biotech, cat. No**. BR-1320, 0.4 mmol/g, 0.05-0.1 mmol scale**). For the incorporation of phosphotyrosine the building block Fmoc-Tyr(PO(OBzl)OH)-OH (Sigma Aldrich/ Novabiochem®, Cat. No. 8520710001) was used. Couplings were achieved by reacting 0.2 M Fmoc-AA-OH with 0.25 M DIC and 0.25 M Oxyma in DMF. A solution of 20% Piperidine in DMF was used to remove the Fmoc protection group. Crude peptides were washed five times with dry diethyl ether. Final deprotection was done by adding 95:2.5:2.5 of TFA/TIS/H_2_O for 2-3 hours at 25 ˚C. Peptides were purified by preparative reverse phase-high performance liquid chromatography (RP-HPLC) on using gradient: A = H_2_O +0.1% TFA, B = MeCN + 0.1% TFA, 0-60 min, 20-80% B in A, flowrate: 10 mL/min). Products were obtained as a white powder after lyophilization and analyzed by UPLC-UV and HR-MS.

Peptide **bio-pY**

Isolated yield: 26 mg (17 µmol, 34 %) starting from 50 µmol scale.

**HR-MS (ESI)** *m*/*z* calcd. for C_64_H_97_N_16_O_25_PS^2+^: 777.3210 [M+2H^+^]; found: 777.3190.


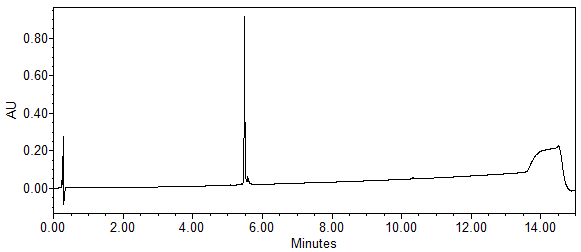


Peptide **bio-spY**

Isolated yield: 9.8 mg (6.3 µmol, 25 %) starting from 25 µmol scale.

**HR-MS (ESI)** *m*/*z* calcd. for C_64_H_97_N_16_O_25_PS^2+^: 777.3210 [M+2H^+^]; found: 777.3190.


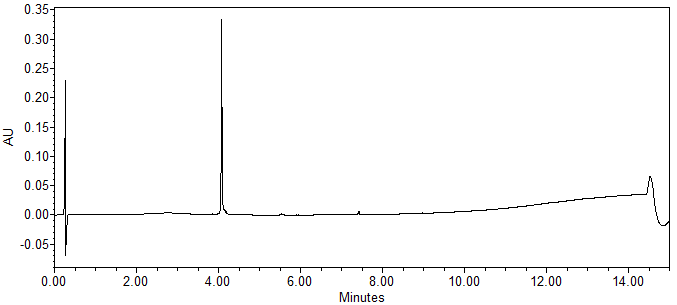


## **Organic synthesis**

### **Di-(4-acetoxy benzyl) ethynylphosphonite (1)**

Di-(4-acetoxy benzyl) ethynylphosphonite **1** was synthesized according to a modified literature procedure for the synthesis of unsaturated phosphonites.^[5-6]^ A 25-mL Schlenk flask was loaded with 267 mg bis(diisopropylamino)chlorophosphine (Sigma-Aldrich, 1.0 mmol, 1.0 eq.) under an argon atmosphere, cooled to −78 °C and 2.2 mL ethynylmagnesium bromide solution (Sigma-Aldrich, 0.5 M in THF, 1.1 mmol, 1.1 eq.) was added drop wise. The yellowish solution was allowed to warm to room temperature and stirred for 30 minutes. A solution of 415 mg 4-acetoxybenzyl alcohol (Sigma-Aldrich, 2.5 eq., 2.5 mmol) in 5.6 mL tetrazole (0.45 M in MeCN, 2.5 mmol, 2.5 eq.) was added and the suspension was stirred for 2 hours at room temperature. The reaction mixture was directly applied on a silica gel flash column for purification (30% EtOAc in hexane) and compound **1** was obtained as a pale oil (233 mg, 0.6 mmol, 60%), which was directly used for subsequent reactions with peptides.

**^1^H NMR** (600 MHz, CDCl_3_) δ 7.37 – 7.30 (m, 4H), 7.15 – 7.04 (m, 4H), 5.01 – 4.90 (m, 4H), 3.20 (d, *J* = 2.2 Hz, 1H), 2.32 (s, 6H).

**^31^P NMR** (243 MHz, CDCl_3_) δ 131.19.

**^13^C NMR** (151 MHz, CDCl_3_) δ 169.4, 150.4, 135.3 (d, *J*_C-P_= 4.3 Hz), 128.9, 121.7, 92.7, 84.5 (d, *J*_C-P_ = 47.8 Hz), 69.3 (d, *J*_C-P_ = 6.8 Hz), 21.1.

*Further characterization by HR-MS was performed after Staudinger-reaction with azido-peptides (see section 3.2.1.2).*

### **1-(benzyloxy)-1-ethynyl-*N,N*-diisopropylphosphanamine (2)**

A 25-mL Schlenk flask was loaded with 267 mg bis(diisopropylamino)chlorophosphine (Sigma-Aldrich, 1.0 mmol, 1.0 eq.) under an argon atmosphere, cooled to −78 °C and 2.2 mL ethynylmagnesium bromide solution (Sigma-Aldrich, 0.5 M in THF, 1.1 mmol, 1.1 eq.) was added drop wise. The solution was allowed to warm to room temperature and stirred for 30 minutes. A solution of 104 µL benzyl alcohol (1.0 eq., 1.0 mmol) in 2.2 mL tetrazole (0.45 M in MeCN, 1.0 mmol, 1.0 eq.) was added and the suspension was stirred for 2 hours at room temperature. The transformation was verified by ^31^P NMR (shift of **2** at ca. 95 ppm) and the entire crude reaction mixture was further used without purification when **2** constituted more than 70% of the total phosphorus-containing species, as determined by ³¹P NMR signal integration.

### **1-ethoxy-1-ethynyl-*N,N*-diisopropylphosphanamine (3)**

A 25-mL Schlenk flask was loaded with 267 mg bis(diisopropylamino)chlorophosphine (Sigma-Aldrich, 1.0 mmol, 1.0 eq.) under an argon atmosphere, cooled to −78 °C and 2.2 mL ethynylmagnesium bromide solution (Sigma-Aldrich, 0.5 M in THF, 1.1 mmol, 1.1 eq.) was added drop wise. The solution was allowed to warm to room temperature and stirred for 30 minutes. A solution of 56 µL of dry ethanol (1.0 eq., 1.0 mmol) in 2.2 mL tetrazole (0.45 M in MeCN, 1.0 mmol, 1.0 eq.) was added and the suspension was stirred for 2 hours at room temperature. The reaction was then followed by ^31^P NMR. Phosphonamidite **3** presents a shift of ca. 93 ppm and after verifying the transformation the entire crude mixture was further used without purification.

### **Synthetic route to SM-PN**

#### **N-(4-azido phenethyl) biotinamide (4)**

A 25-mL round-bottom flask was charged with 205 mg of biotin-N-hydroxysuccinimide (NHS) ester (MedChemExpress, 0.6 mmol, 1.2 eq.) dissolved in 3.0 mL of dry DMF and 262 µL DIPEA (1.5 mmol, 3.0 eq.). 100 mg 2-(4-azidophenyl) ethylamine hydrochloride ^[7]^ (0.5 mmol, 1.0 eq.) were added and the resulting yellow solution was stirred for 1 h. The solvents were removed under reduced pressure, 50 mL water was added, and the suspension was basified with 1 N NaOH and extracted three times with EtOAc. The combined organic fractions were washed two times with water, dried over MgSO_4_ and all volatiles were removed under reduced pressure. The product **4** was obtained as a yellowish powder (191 mg, 0.49 mmol, 98%).

**^1^H NMR** (300 MHz, DMSO-*d*_6_) δ 7.86 (t, *J* = 5.6 Hz, 1H), 7.24 (d, 2H), 7.03 (d, 2H), 6.40 (d, *J* = 18.5 Hz, 2H), 4.30 (dd, *J* = 7.8, 5.0 Hz, 1H), 4.12 (ddd, *J* = 7.4, 4.5, 1.8 Hz, 1H), 3.24 (q, *J* = 6.8 Hz, 2H), 3.12 – 3.02 (m, 1H), 2.90 – 2.72 (m, 1H), 2.68 (t, *J* = 7.2 Hz, 2H), 2.58 (d, *J* = 12.4 Hz, 1H), 2.02 (t, *J* = 7.3 Hz, 2H), 1.63 – 1.55 (m, 1H), 1.45 (h, *J* = 7.0, 6.4 Hz, 3H), 1.31 – 1.21 (m, 2H).

**^13^C NMR** (75 MHz, DMSO-*d*_6_) δ 172.4, 163.2, 137.6, 137.1, 130.7, 119.4, 61.5, 59.7, 55.9, 35.6, 34.9, 28.6, 28.5, 25.8.

**HR-MS (ESI) m/z** calcd. for C_18_H_25_N_6_O_2_S^+^: 389.1754 [M+H] ^+^, found: 389.1763.

#### **O-4-acetoxy-benzyl-N -(4-(2-biotinamidoethyl)phenyl)-P-ethynylphosphonamidate (5)**

In a round bottom flask flushed with nitrogen, 156 mg of N-(4-azido phenethyl) biotinamide **4** (0.4 mmol, 1.0 eq.) was stirred together with 154 mg of di-(4-acetoxy benzyl) ethynylphosphonite 1 (0.4 mmol, 1.0 eq.) in 2.0 mL dry DMF (0.2 mmol/mL) overnight. The organic solvent was removed under reduced pressure and the residue purified by preparative RP-HPLC. Compound **5** was obtained as a yellowish powder (89 mg, 0.15 mmol, 38 %).

**^1^H NMR** (300 MHz, DMSO-*d*_6_) δ 8.47 (d, *J* = 8.8 Hz, 1H), 7.49 – 7.39 (m, 2H), 7.17 – 7.10 (m, 2H), 7.09 – 6.96 (m, 4H), 5.25 – 4.95 (m, 2H), 4.42 (d, *J* = 12.7 Hz, 1H), 4.21 (ddd, *J* = 56.3, 7.7, 4.5 Hz, 2H), 3.25 – 3.14 (m, 2H), 3.07 (dt, *J* = 8.4, 5.8 Hz, 1H), 2.82 (dd, *J* = 12.5, 5.0 Hz, 1H), 2.64 – 2.56 (m, 2H), 2.26 (s, 3H), 2.03 (t, *J* = 7.3 Hz, 2H), 1.69 – 1.34 (m, 4H), 1.34 – 1.20 (m, 2H).

**^31^P NMR** (122 MHz, DMSO-*d*_6_) δ -9.16.

**^13^C NMR** (75 MHz, DMSO-*d*_6_) δ 172.4, 169.7, 163.2, 150.9, 138.2, 133.8 (d, *J*_C-P_ = 8.1 Hz), 133.1, 129.7 (d, *J*_C-P_ = 3.4 Hz), 122.4, 118.4 (d, *J*_C-P_ = 7.5 Hz), 91.8 (d, *J*_C-P_ = 45.6 Hz), 75.8, 66.3 (d, *J*_C-P_ = 4.4 Hz), 61.5, 59.7, 55.9, 35.7, 34.9, 28.7, 28.5, 25.8, 21.3.

**HR-MS (ESI) m/z** calcd. for C_29_H_35_N_4_O_6_PS^+^: 599.2088 [M+H] ^+^, found: 599.2084.

#### **N-(4-(2-biotinamidoethyl)phenyl)-P-ethynylphosphonamidic acid (6) – SM-PN**

Phosphonamidate **5** (20 mg, 0.033 mmol) was dissolved in a volume of 0.5 mL Tris buffer pH 7.4 containing 20% DMSO. Another 0.5 mL of a 50 μM stock solution of esterase form porcine liver in Tris buffer pH 7.4 was added. The mixture was shaken for one hour, diluted with 3.0 mL of 10 mM NH_4_OAc pH 8.5 and directly purified by semi-preparative RP-HPLC using a basic eluent system (A: 100% 10 mM NH_4_OAc pH 8.5, B: 90% MeCN + 10% A). After lyophilization product **6** was isolated as a colorless oil (10.5 mg, 0.023 mmol, 71%).

**^1^H NMR** (300 MHz, DMSO-*d*_6_) δ 7.82 (s, 1H), 7.06 – 6.94 (m, 2H), 6.92 – 6.79 (m, 2H), 6.43 (d, *J* = 6.4 Hz, 1H), 4.40 – 4.23 (m, 1H), 4.17 – 4.05 (m, 1H), 3.20 – 3.06 (m, 4H), 3.03 – 2.97 (m, 1H), 2.90 – 2.77 (m, 1H), 2.03 (t, *J* = 7.3 Hz, 2H), 1.90 (d, *J* = 1.9 Hz, 2H), 1.53 (dq, *J* = 40.7, 7.9, 7.2 Hz, 4H), 1.27 (dt, *J* = 11.7, 6.6 Hz, 2H).

**^31^P NMR** (122 MHz, DMSO- *d*_6_) δ -17.85.

**^13^C NMR** (75 MHz, DMSO-*d*_6_) δ 171.9, 162.8, 142.8, 128.3 (d, *J*_C-P_ = 4.4 Hz), 116.4 (d, *J*_C-P_ = 6.8 Hz), 86.8 (d, *J*_C-P_ = 57.5 Hz), 79.8 (d, *J*_C-P_ = 35.6 Hz), 64.3, 61.1, 59.2, 55.5, 35.4, 34.6, 28.23, 28.1, 25.4, 21.2.

**HR-MS (ESI) m/z** calcd. for C_20_H_27_N_4_O_4_PS^+^: 451.1564 [M+H] ^+^, found: 451.1563.

### **Synthetic route to SM-PO**

#### **O-(4-acetoxybenzyl)-O-(N-Boc-tyraminyl)-P-ethynylphosphonate (7)**

A 25-mL Schlenk flask was charged with 534 mg bis(diisopropylamino)chlorophosphine (Sigma-Aldrich, 2.0 mmol, 1.0 eq.) under an argon atmosphere, cooled to −78 °C and 4.4 mL ethynylmagnesium bromide solution (Sigma-Aldrich, 0.5 M in THF, 2.2 mmol, 1.1 eq.) was added drop wise. The resulting solution was allowed to warm to room temperature and stirred for 30 minutes. A solution of 332 mg 4-acetoxybenzyl alcohol (Sigma-Aldrich, 2.0 mmol 1.0 eq.) in 4.5 mL tetrazole (0.45 M in MeCN, 2.0 mmol, 1.0 eq.) was added and the suspension was stirred for 2 hours at room temperature. Subsequently, a mixture of 476 mg *N*-Boc-tyramine (Sigma-Aldrich, 2.0 mmol, 1.0 eq.) and 4.5 mL tetrazole (0.45 M in MeCN, 2.0 mmol, 1.0 eq) was mixed in and the suspension was stirred overnight at room temperature. The next day, 0.37 mL of di-*tert.*-butylperoxide (Sigma-Aldrich, 2.0 mmol, 1.0 eq.) were added and stirred for one hour. Afterwards, 50 mL water was added, and the suspension was extracted three times with EtOAc. The combined organic fractions were washed two times with water, dried over MgSO_4_ and all volatiles were removed under reduced pressure. The crude was dry-loaded onto silica gel for flash column chromatography (50% EtOAc in hexane) and compound **7** was obtained as a white oil (245 mg, 0.52 mmol, 26%).

**^1^H NMR** (600 MHz, CDCl_3_) δ 7.40 (d, J = 8.5 Hz, 2H), 7.18 – 7.02 (m, 6H), 5.20 (dd, J = 9.1, 1.3 Hz, 2H), 3.32 (q, J = 6.8 Hz, 2H), 3.00 (d, J = 14.0 Hz, 1H), 2.74 (t, J = 7.1 Hz, 2H), 2.28 (s, 3H), 1.41 (s, 9H).

**^31^P NMR** (243 MHz, CDCl_3_) δ -12.18.

**^13^C NMR** (151 MHz, CDCl_3_) δ 169.3, 155.8, 151.0, 148.1 (d, *J*_C-P_ = 7.2 Hz), 136.6, 132.5 (d, *J*_C-P_ = 7.3 Hz), 121.9, 120.6 (d, *J*_C-P_ = 4.6 Hz), 89.8 (d, *J*_C-P_ = 52.7 Hz), 79.2, 73.5 (d, *J*_C-P_ = 302.7 Hz), 68.7 (d, *J*_C-P_ = 5.5 Hz), 41.7, 35.5, 28.4, 21.1.

**HR-MS (ESI) m/z** calcd. for the deprotected species C_24_H_28_NO_7_P^+^ [M − C₅H₉O₂ + H]^+^: 374.1149, found: 374.1154.

****Observed mass corresponds to the [M − Boc + H]⁺ ion, consistent with common Boc loss during ESI ionization.***

#### **O-(4-acetoxybenzyl)-O-(tyraminyl)-P-ethynylphosphonate (8)**

####

*O*-(4-acetoxybenzyl)-*O*-(*N*-Boc-tyraminyl)-*P*-ethynylphosphonate **7** (100 mg, 0.21 mmol, 1.0 eq.) was mixed with 0.4 mL (0.2M) TFA containing 5% H_2_O and stirred for 10 minutes at room temperature. After verifying the Boc cleavage by UPLC-UV/MS, 15 mL of H_2_O were added, the mixture was lyophilized and the oily residue used in the next step without further purification.

#### **4-(2-biotinamidoethyl)phenyl ethynylphosphonic acid monoester (9)**

A 5 mL round bottom flask was charged with 60 mg of *O*-(4-acetoxybenzyl)-*O*-(tyraminyl)-*P*-ethynylphosphonate **8** (0.16 mmol, 1.0 eq.) in 0.1 M DMF (1.6 mL). To this 65 mg of biotin-NHS (MedChemExpress, 0.2 mmol, 1.2 eq.) and 35 µL DIPEA (0.2 mmol, 1.2 eq.) was added and the solution stirred for one hour at room temperature. The reaction outcome was evaluated by UPLC-UV/MS, which revealed that under these conditions, the 4-acetoxy substituent was removed, yielding the final desired product. The solvent was removed *in vacuo*, the residue dissolved in 4 mL of 15% MeCN and 85% H_2_O containing 0.1% TFA and was immediately purified by HPLC (gradient: A = H2O +0.1% TFA, B = MeCN + 0.1% TFA, 0-50 min, 15-80% B in A, flowrate: 10 mL/min). After lyophilization the product was obtained as a white powder (48 mg, 0.11 mmol, 68%).

**^1^H NMR** (600 MHz, MeOD-d₄) δ 8.78 (d, J = 8.5 Hz, 2H), 8.71 (dd, J = 8.5, 1.5 Hz, 2H), 6.09 (dd, J = 7.9, 4.8 Hz, 1H), 5.88 (dd, J = 7.9, 4.5 Hz, 1H), 5.22 (d, J = 13.4 Hz, 1H), 5.05 – 4.92 (m, 2H), 4.75 (ddd, J = 9.1, 5.8, 4.5 Hz, 1H), 4.51 (dd, J = 12.8, 5.0 Hz, 1H), 4.35 (t, J = 7.1 Hz, 2H), 4.28 (d, J = 12.8 Hz, 1H), 3.75 – 3.67 (m, 2H), 3.30 – 3.08 (m, 4H), 2.96 – 2.87 (m, 2H).

**^31^P NMR** (243 MHz, MeOD-d₄) δ -13.66.

**^13^C NMR** (151 MHz, MeOD-d₄) δ 176.0, 165.9, 150.4 (d, *J*_C-P_ = 7.3 Hz), 137.3, 130.9, 121.6 (d, *J*_C-P_ = 4.7 Hz), 89.6 (d, *J*_C-P_ = 51.0 Hz), 77.1 (d, *J*_C-P_ = 290.2 Hz), 63.5, 61.8, 56.8, 49.1, 49.0, 48.9, 48.7, 48.6, 41.6, 40.8, 36.6, 35.5, 29.5, 29.3, 26.8.

**HR-MS (ESI) m/z** calcd. for C_20_H_26_N_3_O_5_PS^+^: 452.1401 [M+H] ^+^, found: 452.1386.

### **Synthetic route to TMR-BBP (reported phosphatase warhead)**

The synthetic protocol for TMR-BBP was adapted from previously published procedures. ^[3,8-9]^ Compounds **10**, **11** and **12** have already been synthesized and characterized.

#### **Diethyl ((4-(N-Boc-aminomethyl)phenyl)(hydroxy)methyl)phosphonate (10)**

A 25 mL round bottom flask was loaded with 0.75 g of 4-(Aminomethyl)benzaldehyde, N-boc protected (Sigma-Aldrich, 3.2 mmol, 1.0 eq.) in 10 mL dry DMF (0.32 M). To this, 650 µL (691 mg, 5.0 mmol, 1.6 eq.) of diethyl phosphonate and 0.9 g of cesium fluoride (6.4 mmol, 2.0 eq.) were added and the solution was stirred at 60 ˚C for 4 hours. Afterwards, 50 mL EtOAc was added, and the resulting solution was extracted three times with water (50 mL) and 3 times with brine (50 mL). The combined organic fractions were dried over MgSO_4_, filtered and purified by column chromatography (100% EtOAc) to yield compound **10** as a pale oil (754 mg, 2.0 mmol, 63%) in sufficient purity for next steps.

**^1^H NMR** (600 MHz, DMSO-*d*_6_) δ 7.36 (dd, *J* = 8.3, 2.3 Hz, 2H), 7.19 (d, *J* = 7.8 Hz, 2H), 6.14 (dd, *J* = 15.4, 5.8 Hz, 1H), 4.11 (d, *J* = 6.2 Hz, 1H), 4.03 (q, *J* = 7.1 Hz, 1H), 4.01 – 3.84 (m, 4H), 1.39 (s, 8H), 1.16 (dt, *J* = 19.0, 7.0 Hz, 7H).

**^31^P NMR** (243 MHz, DMSO-*d*_6_) δ 21.78.

**^13^C NMR** (151 MHz, DMSO) δ 156.3, 139.8, 137.2, 127.7, 127.7 (d, *J*_C-P_ = 5.8 Hz), 78.2, 69.6 (d, *J*_C-P_ = 162.8 Hz), 62.6 (d, *J*_C-P_ = 7.0 Hz), 62.3 (d, *J*_C-P_ = 6.9 Hz), 43.6, 28.7, 16.8 (d, *J*_C-P_ = 5.3 Hz), 16.7 (d, *J*_C-P_ = 5.2 Hz).

**UPLC-MS (ESI)** *m/z* calcd. for C_17_H_28_NO_6_P^+^: 396.17 [M+Na]^+^; found: 396.31.

#### **Diethyl (bromo(4-(N-Boc-aminomethyl)phenyl)methyl)phosphonate (11)**

A 25 mL schlenk flask was charged with 373 mg of diethyl ((4-(N-Boc-aminomethyl) phenyl)(hydroxy)methyl)phosphonate **10** (1.0 mmol, 1.0 eq.) dissolved in 10 mL of 50:50 dry MeCN and dry CH_2_Cl_2_ (0.1 M) and cooled down to 0 ˚C. To this, 845 mg (2.0 mmol, 2.0 eq.) of triphenylphosphine bromide and 178 µL of dry pyridine (2.2 mmol, 2.2 eq.) were added and the reaction was stirred for 30 min. The solution was allowed to warm to room temperature and was then stirred overnight. After the reaction outcome was verified by UPLC-UV/MS, all volatiles were removed under reduced pressure and the crude mixture was used in the next step.

#### **Diethyl ((4-(aminomethyl)phenyl)bromomethyl)phosphonate (12)**

The full amount of crude intermediate **11** (assumed 1.0 mmol) was loaded in a 25 mL round bottom flask and was stirred with 5 mL of neat TFA for 10 minutes at room temperature. TFA was co-evaporated with 50 mL CH_2_Cl_2_ under reduced pressure (repeated three times). The residue was dissolved in 20 mL 1 M HCl to extract the amine into the aqueous layer via protonation and organic impurities like triphenylphosphine were extracted with EtOAc (3 x 50 mL). Subsequently, the aqueous layer was basified with 1 M NaOH to pH 10 to deprotonate the free amine and was extracted with EtOAc (3 x 50 mL). The combined organic fractions were dried over MgSO_4_ and all volatiles were removed under reduced pressure. Compound **12** was obtained in sufficient purity as a pale waxy solid. (205 mg, 0.61 mmol, 61% from **10**)

**^1^H NMR** (600 MHz, CDCl_3_) δ 7.43 (dd, *J* = 8.3, 1.8 Hz, 2H), 7.23 (d, *J* = 8.0 Hz, 2H), 4.79 (d, *J* = 12.9 Hz, 1H), 4.11 (dtd, *J* = 11.8, 7.2, 3.2 Hz, 2H), 4.00 – 3.74 (m, 4H), 1.24 (t, *J* = 7.1 Hz, 3H), 1.07 (t, *J* = 7.1 Hz, 3H).

**^31^P NMR** (243 MHz, CDCl_3_) δ 16.94.

**^13^C NMR** (151 MHz, DMSO) δ 146.8, 142.6, 130.1 (d, *J*_C-P_ = 6.2 Hz), 129.6, 127.7, 63.9 (d, *J*_C-P_ = 6.4 Hz), 63.7 (d, *J*_C-P_ = 6.4 Hz), 49.0, 42.2, 16.7 (d, *J*_C-P_ = 5.5 Hz), 16.6 (d, *J*_C-P_ = 5.3 Hz).

**UPLC-MS (ESI)** m/z calcd. for C_12_H_19_BrNO_3_P^+^ [M+Na]^+^: 358.03, 360.03 (isotopes ^79Br/^81Br); found: 358.11, 360.12.

#### **Diethyl (bromo(4-(N-5/6-TAMRA-aminomethyl)phenyl)methyl)phosphonate (13)**

A 10 mL round bottom flask was loaded with 30 mg of **12** (0.1 mmol, 1.0 eq.) and 52 mg 5/6-TAMRA-NHS ester (BLD Pharm, 0.1 mmol, 1.0 eq.) in 2 mL dry DMF (0.05 M). To this solution 36 µL of DIPEA (0.2 mmol, 2.0 eq.) was added and the reaction was stirred for 1 hour at room temperature. Afterwards, 20 mL of CH_2_Cl_2_ were added and the product was extracted three times with water (50 mL) and three times with brine (50 mL). The organic fraction was dried over MgSO_4_, filtered and the solvent was removed under reduced pressure. Product **13** was used without further purification for the next step.

#### **α-bromo(4-((N-5/6-TAMRA-aminomethyl)phenyl)methyl)phosphonic acid (14)**

In a 10 mL schlenk flask under an argon atmosphere, crude **13** (46 mg, 0.063 mmol, 1.0 eq.) was suspended in 4 mL dry CH_2_Cl_2_ (0.015 M) and cooled to −30 °C before dropwise addition of TMSBr (112 µL, 0.94 mmol, 15 eq.). The reaction was allowed to warm to room temperature after 30 minutes and was stirred overnight. The next day, 15 mL of MeOH was added and all volatiles removed *in vacuo*. The residue was dissolved in a mixture of 20% MeCN and 80% H_2_O containing 0.1% TFA and was immediately purified by HPLC (gradient: A = H2O +0.1% TFA, B = MeCN + 0.1% TFA, 0-60 min, 20-80% B in A, flowrate: 10 mL/min). After lyophilization the product was obtained as a pink powder (28 mg, 0.04 mmol, 63%).

**^31^P NMR** (243 MHz, MeOD-d₄) δ 14.95.

**HR-MS (ESI)** m/z calcd. for C_33_H_31_BrN_3_O_7_P^+^ [M+H]^+^: 692.1153, 694.1153 (isotopes ^79Br/^81Br); found: 692.1098, 694.1132.


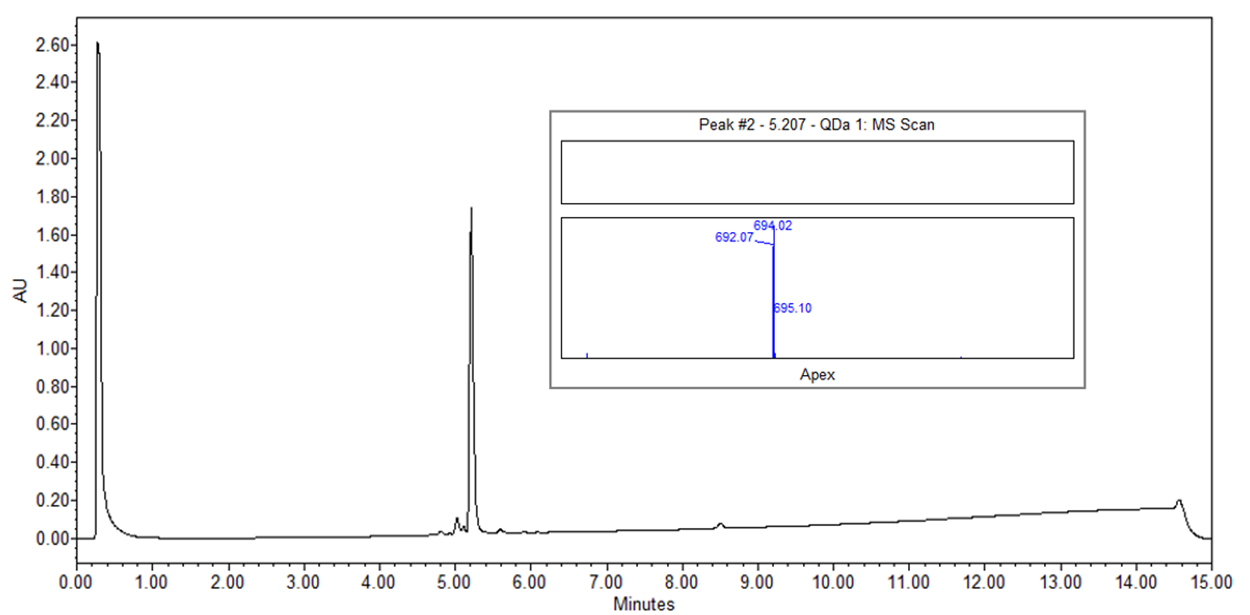


## **pH and lysate stability studies**

Stability of **SM-PN** and **SM-PO in buffer**

Internal standard triphenylphosphine oxide (TPPO, 12 µL of a 100 mM stock in DMSO, final concentration 2 mM) and **SM-PN** or **SM-PO** (36 µL of a 50 mM stock in DMSO, final concentration 3 mM) were added in microcentrifuge tubes containing 552 µL of each test buffer solution (0.1% TFA pH 2, 50 mM **NaOAc** pH 3.5, 50 mM Tris pH 7.4, 50 mM Tris pH 8.5, 50 mM TEAB pH 11.0, containing 10% D_2_O (492 μL buffer and 60 μL D_2_O) and mixed right before the measurement. The mixtures were transferred to NMR tubes and one-dimensional ^31^P-NMR spectra were recorded for each sample at room temperature, in intervals of 4 h over the course of 48 h on a Bruker AV-III 600 or AV-III 300 spectrometer. For analysis, the spectra were plotted in MestReNova and the intensity of the ^31^P-NMR signal was analyzed by integration.

Stability of **bio-PN** and **bio-PO in HEK293T cell lysate**

A solution of 480 µL of HEK293T cell lysate (1 mg/mL) was supplemented with 60 µL of D_2_O. To this, 60 µL of **bio-PN** or **bio-PO** (10 mM stock in DMSO, final concentration 1 mM) were added right before the start of the measurement. The mixtures were transferred to NMR tubes and one-dimensional ^31^P-NMR spectra were recorded for each sample at room temperature, in intervals of 2.5 h over the course of 18 h on a Bruker AV-III 600 spectrometer. For analysis, the spectra were Fourier-transformed, baseline corrected and the intensity of the ^31^P-NMR signal was obtained by integration.

## **Thiol reactivity studies with glutathione**

For testing the thiol reactivity of **bio-PN** and **bio-PO** at pH 7.4, 10 µL of a 10 mM caffeine (internal standard) stock solution in Tris buffer pH 7.4 (final concentration 1 mM) and 10 µL of a 10 mM peptide stock solution in DMSO were added in 75 µL of 50 mM Tris buffer, pH 7.4. Immediately prior to the measurement, 5 µL of a 200 mM stock solution of reduced glutathione (final concentration 10 mM, 10.0 eq.) in Tris buffer (pH adjusted to 7.4) were added. The solutions were transferred to a sample vial and measured by UPLC-UV/MS at room temperature and at time points t_0_, t_0.5h_, t_1h_, t_2h_, t_4h_, t_8h_, t_16h_ 1 µL of the reaction mixture was injected. For the analysis, the peak area of the internal standard was correlated with that of the starting material. The same protocol was followed at pH 8.5 using Tris buffer adjusted accordingly.

Similar conditions were applied in the case of **SM-PN** and **SM-PO**. Specifically, in 87 µL of 50 mM Tris buffer, pH 7.4 or 8.5 were added 1 µL of a 100 mM TPPO (internal standard) stock solution in DMSO (final concentration 1 mM) and 2 µL of a 50 mM DMSO stock solution of **SM-PN** or **SM-PO**. Finally, 10 µL of reduced glutathione (100 mM stock solution adjusted in the desired pH, final concentration 10 mM, 10.0 eq.) were mixed in and the same measurement and analysis protocol as before was employed.

## **Recombinant protein labeling**

For the labeling of recombinant proteins PTP1B, TEV protease and human serum albumin (New England biolabs) with the electrophilic peptides **bio-PN** and **bio-PO**, proteins were adjusted to a concentration of 30 µM in 50 mM HEPES pH 7.4, 150 mM NaCl and 0.5 mM TCEP. From each solution 48.5 µL were mixed with 1.5 µL of 10 mM peptide stock (300 µM final concentration, 10.0 eq.) and incubated at room temperature, at 450 rpm and the reaction was monitored over the course of 20 hours. PTP1B was additionally incubated with the same equivalents of **SM-PN** or **SM-PO**. Samples were collected at various time points, diluted as necessary, and analyzed by intact protein mass spectrometry using a QToF instrument.

## **LC-MS/MS for labeling site identification**

Labeling reaction & In-gel digestion

Freshly thawed PTP1B (in HEPES pH 7.4, 150 mM NaCl, 0.5 mM TCEP) was adjusted to a concentration of 30 µM. A volume of 48.5 µL of PTP1B solution were mixed with 1.5 µL of a 10 mM stock solution in DMSO of either **bio-PN** or **bio-PO**. The reaction was incubated at room temperature and 650 rpm for 40 hours in total. At time points t_0_, t_2h_, t_4h_, t_18h_, t_40h_ 2 µL of the reaction mixture and 3 µL of the buffer were mixed with 5 µL of Laemmli buffer containing β-mercaptoethanol and boiled at 95 °C for 5 min. The samples were subjected to SDS-PAGE and proteins were stained by Coomassie brilliant blue. Immediately after, gel bands were excised and incubated with 50% acetonitrile (MeCN) in 50 mM triethylammonium bicarbonate (TEAB) pH 8.5 at 30 °C for 10 min. After removing the washing buffer, 50 mM TEAB buffer was added, incubated, and removed. Gel pieces were then dehydrated with two consecutive MeCN washes. Proteins were reduced with 5 mM DTT in 50 mM TEAB at 56 °C for 45 min, then alkylated with 40 mM chloroacetamide (CAA) in 50 mM TEAB at 25 °C for 30 minutes. Gel pieces were washed and dehydrated as before. Digestion was performed by incubating the gel pieces with 0.1 µg trypsin in 50 mM TEAB for 16 h at 37 °C. The reaction was stopped by adding three volumes of 0.5% TFA in MeCN. The supernatant was dried along with peptides extracted from the gel pieces after an additional MeCN wash.

Liquid chromatography and mass spectrometry for the identification of cysteine modifications

Desalted peptides were resuspended in 1 % MeCN with 0.05 % TFA and 0.5 µg were injected into a Thermo Scientific Vanquish Neo system connected to a PepMap C-18 trap-column (0.075 mm x 50 mm, 3 μm particle size, 100 Å pore size, Thermo Fisher Scientific) followed by an in-house packed C18 column for reverse phase separation (Poroshell 120 EC-C18, 2.7 μm, Agilent Technologies). With a flowrate of 250 nL/min, peptides were separated using a 117 min gradient of increasing MeCN concentration and analyzed on an Orbitrap Exploris 480 mass spectrometer (Thermo Fisher Scientific) equipped with a FAIMS Pro interface and Instrument Control Software version 4.2. MS1 scans were acquired in the Orbitrap with a mass resolution of 120,000. MS1 parameters were as following: scan range m/z 375 – 1,200, 300% normalised AGC target, automatic maximum injection time. MS2 scans were acquired in the Orbitrap with the following parameters: 30,000 mass resolution, automatic scan range, standard AGC target, automatic maximum injection time, isolation window 1.6 m/z, NCE 30%. Previously isolated precursors were excluded from fragmentation for 20 s. Only precursors with charges +2 – +4 (or in case of bio-PO in a separate injection also +3 – +8) were subjected to MS2. Data acquisition cycled between FAIMS compensation voltages (CVs) -50 and -70 with a cycle time of 2 seconds per CV. For the quantification plot in Figure 6e, we used the mean peptide intensity from the bio-PO samples acquired with the two different charge filters.

Identification and quantification of cysteine modifications

The raw data files were analyzed using FragPipe version 22.0.^[10-11]^ Data were searched against a database containing common contaminants, the sequence of PTP1B and reverse decoys. MSFragger was run with the following parameters: 10 ppm MS1 tolerance, 20 ppm MS2 tolerance, 10 – 50 peptide length, 500 – 5,000 Da peptide mass range, and trypsin digestion with maximum 2 missed cleavages. Oxidation of methionine (+15.9949 Da), protein N-terminal acetylation (+42.0106 Da), carbamidomethylation of cysteine (+57.0215 Da), and +105.9820 Da (for **bio-PN**, as upon sample preparation cleavage of the labile P-N bond removes the rest of the peptide) or +1560.6378 (for **bio-PO**, full peptide mass) on cysteine were set as variable modifications. PTMProphet was run for site localization. IonQuant was run to compute the LFQ with normalization of intensity across runs activated and match between runs deactivated. Only sites with a localization probability > 0.75 were considered. Exemplary spectra were exported using the FragPipe integrated PDV viewer.

## **Microscale thermophoresis (MST)**

First, PTP1BC215A was fluorescently labeled using Nanotemper RedNHS labeling kit (NanoTemper Technologies, cat. No. MO-L011) in accordance with the manufacturer's instructions. MST measurements were performed on a NanoTemper Monolith TM NT.115 Nano instrument. Measuring conditions were 50 nM RedNHS labeled PTP1BC215A in PBS-Tween20 (0.05%), using 40% LED excitation and medium MST power at 22 °C. The fluorescently labeled protein was tested for homogeneity prior to the experiment by comparing the Fnorm values of at least three samples. If the variability of absolute values was more than 8%, the samples were considered unfit for measurement. The same experiment was repeated with added ligand to determine the highest tolerable ligand concentration. For the titration experiment, peptide ligands were serially diluted and mixed in a 1:1 (v:v) ratio with the labeled interaction partner to obtain 16-18 samples. For pY and spY the concentration range spanned from 0.25 mM down to 7.6 nM, while for **bio-PN** and **bio-PO** additional conditions included higher concentrations of 0.5 mM and 1 mM. The samples were examined for initial fluorescence values and samples deviating by more than 10% from the average were excluded. Each sample was then measured at least in triplicates to obtain the average Fnorm value for K_D_ determination. The results were analyzed using the software MO.Affinity Analysis v2.3 (NanoTemper Technologies).

## **Kinetic characterization by *p*NPP assay**

For measuring enzyme kinetics for the labeling of PTP1B by the electrophilic peptides the well-established the p-nitrophenyl phosphate (pNPP) assay was employed.^[12]^ Briefly, reactions were prepared in a final volume of 100 µL in 96-well plates containing phosphatase buffer (50 mM HEPES pH 7.4, 150 mM NaCl and 0.5 mM TCEP), 2 mM *p*NPP, a range of concentrations of **bio-PN** and **bio-PN** (2mM, 1.5 mM, 1 mM, 0.75 mM, 0.5 mM - final DMSO concentrations adjusted to maximum 5 %) and 2 µM purified PTP1B. The peptides were preincubated with the enzyme for total 60 (**bio-PO**) or 90 (**bio-PN**) minutes at room temperature. At certain time intervals, *p*NPP substrate was added and the mixture incubated at 30 ˚C for 30 minutes. The reaction was stopped by adding 50 µL of 1 M NaOH, and the release of p-nitrophenol was quantified by measuring absorbance at 405 nm using a plate reader (TECAN, Switzerland). All experiments were performed in triplicates, and background absorbance from substrate-only controls was subtracted from all readings for all the different time points. For analysis the absorbance was normalized to a 100% active control and inactivation plots were generated for each peptide concentration, correlating absorbance to activity (V). The calculations for the final efficiency of the inactivation are listed below.

**Mathematic considerations for the calculation**

Pseudo–first-order rate constant (*k*_obs_) from time course:

$$\ln\left( \frac{V_{t}}{V_{0}} \right)= -k_{obs} \times t$$

Full hyperbolic Kitz–Wilson equation:

$$k_{obs}=\frac{k_{inact}\times\left[ I \right]}{K_{I}+ \left[ I \right]}$$

No saturation kinetics$: K_{I}\gg\left[ I \right]$_,_$k_{obs}=\left( \frac{k_{inact}}{K_{I}} \right) \times\left[ I \right]$ 🡪 reciprocal: $\frac{1}{k_{obs}}=\frac{K_{I}}{k_{inact}} \times\frac{1}{\left[ I \right]}$

Final relation of efficiency from slope:

$$\frac{k_{inact}}{K_{I}}=\frac{1}{slope}$$

Error propagation for 1/kobs values:

$$SE \left( \frac{1}{k_{obs}} \right)\approx\frac{SE \left( k_{obs} \right)}{k_{obs}^{2}}$$

Error propagation for reciprocal slope:

$$SE \left( \frac{k_{inact}}{K_{I}} \right)\approx\frac{SE \left( slope \right)}{{slope}^{2}}$$

## **Labeling in human lysate – gel scanning and western blotting**

### **General procedure for fluorescence gel scanning and western blotting**

For fluorescence gel scanning of TMR labeled probes samples resolved by SDS-PAGE and first visualized at a Bio-Rad imaging system equipped with a 532 nm laser for excitation. Afterwards, wherever applicable gels were stained with Coomassie brilliant blue.

For western blot analysis, samples resolved by SDS-PAGE were transferred to a polyvinylidene difluoride (PVDF) membrane using a Trans-Blot Turbo (Bio-Rad) semi dry blotting system according to the manufacturer`s instructions. The PVDF membranes were blocked with 5 % milk in Tris-Buffer Saline 0.1% Tween® 20 (TBST) at 25°C for 1 hour. Wherever applicable, for visualization of the total protein amount No-Stain Protein Labeling Reagent (ThermoFischer Scientific) was used according to the manufacturer’s instructions. PTP1B was probed using a primary PTP1B monoclonal antibody (ThermoFischer Scientific, MA5-25642) in a 1:2.500 dilution in 0.5 % milk in TBST at 4°C overnight. This was followed by incubation with a HRP conjugated rabbit polyclonal anti-mouse antibody (Abcam, ab6728) in a dilution of 1:5000 at 25°C for 1 h. For blotting against oxidized PTP1B, a primary PTP1B-oxidized antibody was used (Sigma-Aldrich, MABS456) in a 1:1000 dilution in 0.5 % milk in TBST at 4°C overnight, followed by incubation with a HRP conjugated goat polyclonal anti-chicken antibody (Invitrogen, A16054) in a dilution of 1:5000 at 25°C for 1 h. The HRP signal was detected using the Pierce ECL Western Blotting Substrate (ThermoFisher Scientific) and imaged using the ChemiDoc (Bio-Rad) imaging system. Before probing for biotin, the blot was stripped using 7 mL of Restore Stripping Buffer (Thermo Fisher Scientific, 21059) at 37°C for 15 min. Next, the membrane was blocked with 10 mL 1x ROTI®block (Carl Roth) in TBST. Biotin was probed against using Streptavidin-horse radish peroxidase (HRP) conjugate (Thermo Fisher Scientific, N100) at a 1:5000 dilution for 1 hour at room temperature and the HRP signal was detected as mentioned above.

### **Optimization of lysis conditions**

HEK293T cell pellets were lysed in 50 mM Tris buffer (pH 7.5) containing 150 mM NaCl, with or without 0.5 mM TCEP, using sonication (4 cycles of 1 min at 25% intensity, with 1-minute breaks between cycles). The lysates were then adjusted to a concentration of 2 mg/mL and treated either with DMSO or with a 100 µM **bio-PO** (19 µL lysate, 1 µL probe) for 1 hour at room temperature. Samples were mixed 20 laemmli buffer, resolved by SDS-PAGE and analyzed by western blot. Similarly, for testing the effect of detergents, HEK293T cell pellets were lysed in 50 mM Tris buffer pH, 150 mM NaCl, 0.5 mM TCEP either with 0.1% SDS and 0.1% Triton-X or without. The exact same procedure for labeling and analysis was followed. Lastly for probing the oxidation of the catalytic cysteine in PTP1B upon storage, HEK293T cell lysate, lysed in 50 mM Tris buffer pH, 150 mM NaCl, 0.5 mM TCEP was stored for three months at -70 °C. Afterwards this sample was compared to cell pellets lysed in the same buffer on the day of the analysis. After SDS-PAGE, the samples were analyzed by western blot, probing for oxidized (in the catalytic cysteine-C215) PTP1B.

### **Further evaluation using TMR-PO**

For the comparison of the labeling pattern between **TMR-PO** and a general phosphatase probe (**TMR-BBP**) freshly lysed HEK293T cell lysate was adjusted to 2 mg/mL. For TMR-PO labeling cell pellets were lysed in 50 mM Tris buffer pH 7.4, 150 mM NaCl, 0.5 mM TCEP. For labeling with **TMR-BBP** cells were lysed in 50 mM sodium succinate buffer pH 6.0, 150 mM NaCl, 1 mM EDTA, and 1 mM DTT as previously reported.^[3]^ In the case of **TMR-PO** labeling, 24.5 µL of the corresponding lysate was mixed with 0.5 µL of a 5 mM **TMR-PO** stock solution in DMSO, while for **TMR-BBP**, 24µL of the corresponding lysate was mixed with 1 µL of a 25 mM **TMR-BBP** stock solution in DMSO. The reactions were incubated for 1 hour at room temperature and subsequently analyzed by fluorescence gel scanning.

For the labeling with **TMR-PO** of different mammalian cell lysates, HEK293T, MCF-7 and Ramos cell pellets were freshly lysed (50 mM Tris buffer pH 7.4, 150 mM NaCl, 0.5 mM TCEP) and adjusted to a concentration of 4 mg/mL. A volume of 49 µL of each lysate was mixed with 1 µL of a 5 mM **TMR-PO** stock solution in DMSO and incubated for 1 hour at room temperature. Samples were resolved by SDS-PAGE and analyzed by fluorescence gel scanning.

### **Time course labeling using bio-PO**

For the time course labeling experiment freshly lysed HEK293T, MCF-7 and Ramos lysate (50 mM Tris buffer pH 7.4, 150 mM NaCl, 0.5 mM TCEP) were adjusted to 4 mg/mL. The reaction volume was set at 100 µL. For this, 99 µL of lysate was mixed with 1 µL of a 10 mM **bio-PO** stock solution in DMSO (final concentration 100 µM. At indicated time points (0,1,2,3,4,5,6 and 18 hours) 10 µL samples were drawn and immediately mixed with 10 µL of laemmli buffer containing mercaptoethanol and boiled at 95 °C for 5 min and kept until they were analyzed by western blot.

### **Concentration dependent labeling using bio-PO**

For the concentration dependent labeling experiment presented in Fig. 5a freshly lysed HEK293T, MCF-7 and Ramos lysate (50 mM Tris buffer pH 7.4, 150 mM NaCl, 0.5 mM TCEP) were adjusted to 4 mg/mL. In a total reaction volume of 40 µL, 38 µL of lysate was mixed with 2 µL of the following **bio-PO** stock solutions in DMSO: a 2 mM stock for final concentration 100 µM, a 5 mM stock for final concentration 250 µM and a 10 mM stock for final concentration 100 µM. Additionally a DMSO control was prepared. The solutions were incubated for 4 hours at room temperature and subsequently proteins were resolved by SDS-PAGE and analyzed by western blot.

### **Labeling of H_2_O_2_ treated lysate with bio-PO**

Freshly lysed HEK293T and MCF-7 (50 mM Tris buffer pH 7.4, 150 mM NaCl, 0.5 mM TCEP) were adjusted to 4 mg/mL. For the oxidation of the proteome 1 µL of a 50 mM H_2_O_2_ (30% in water) was added in 100 µL lysate. A volume of 1 µL of a 10 mM **bio-PO** stock in DMSO was mixed with 19 µL of native or oxidized lysate and incubated for 4 hours at room temperature. A DMSO control was prepared as well. After the incubation the mixtures were resolved by SDS-PAGE and analyzed by western blot.

### **Lysate labeling with bio-PO(OEt)**

The synthesis of *O*-ethyl substituted ethynyl P(V) electrophilic peptides is described in section 3.2. For the labeling experiment, 48 µL of freshly lysed MCF-7 (50 mM Tris buffer pH 7.4, 150 mM NaCl, 0.5 mM TCEP) adjusted to a concentration of 4 mg/mL were incubated for 1-4 hours at room temperature with 2 µL of **bio-PO(OEt)** (5 mM stock in DMSO, 200 µM final concentration). Samples were drawn every hour and analyzed by western blot, however already at 1 hour a saturation of the biotin signal was observed.

### **Lysate labeling with SM-PN and SM-PO**

For the concentration dependent labeling with **SM-PN** and **SM-PO** similar experimental conditions were applied. Briefly, freshly lysed MCF-7 lysate (50 mM Tris buffer pH 7.4, 150 mM NaCl, 0.5 mM TCEP) was adjusted to 4 mg/mL. In a total reaction volume of 20 µL, 19 µL of lysate was mixed with 1 µL of the following **SM-PN** or **SM-PO** stock solutions in DMSO: a 0.5 mM stock for final concentration 25 µM a 1 mM stock for final concentration 50 µM, a 2 mM stock for final concentration 100 µM, a 5 mM stock for final concentration 250 µM and a 10 mM stock for final concentration 100 µM. Additionally a DMSO control was prepared. The solutions were incubated for 4 hours at room temperature and subsequently proteins were resolved by SDS-PAGE and analyzed by western blot.

## **Labeling in human lysate – LC-MS/MS-based proteomics**

### **Quantitative proteomic analysis for protein abundance**

For the determination of protein abundance ranks, HEK293T, MCF-7 and Ramos cell pellets were resuspended in 8 M Urea in 50 mM TEAB pH 8.5 and homogenized in a Bioruptor (30s on, 30 s off, 10 cycles). Nucleic acids were broken down using Benzonase. The mixture was supplemented with 5 mM TCEP and 40 mM CAA, 1:200 (enzyme:protein wt:wt) LysC and 1:100 Trypsin for 16 h at 37 °C. Peptides were acidified to final 1% FA and desalted with C18 StageTip. Desalted peptides were resuspended in 1 % MeCN with 0.05 % TFA and 1 µg was injected into a Thermo Scientific Dionex UltiMate 3000 system. Peptides were eluted over a 117 min gradient of increasing MeCN concentration and analyzed on an Orbitrap Fusion mass spectrometer (Thermo Fisher Scientific) with Instrument Control Software version 4.0. MS1 scans were acquired in the Orbitrap with a mass resolution of 120,000. MS1 parameters were as following: scan range m/z 375 – 1,500, default AGC target, 50 ms maximum injection time. MS2 scans were acquired in the linear IonTrap with the following parameters: scan rate rapid, automatic scan range, standard AGC target, 35 ms maximum injection time, isolation window 1.6 m/z, NCE 30%. Previously isolated precursors were excluded from fragmentation for 40 s. Only precursors with charges +2 – +4 were subjected to MS2. Data acquisition cycle time was 1 s.

Raw data files were analyzed with FragPipe version 22.0 and the default LFQ-MBR workflow. ^[10]^ Data were searched against a database containing the human proteome with one protein per gene retrieved from the UniProt. Fragment tolerance was set to 0.5 Da. The abundance rank of a protein in a specific cell type was determined by its topN (N=6) intensity after removing contaminants.

### **Proteomic profiling of bio-PO**

For proteome labelling using the SP2E workflow, a modified protocol by Becker et al. was followed.^[13]^ Freshly thawed MCF-7 cell lysates were adjusted to a concentration of 4mg/mL. The labeling started by mixing 95 µL of lysate (ca. 400 µg per sample) and 5 µL of **bio-PO** (10 mM stock solution in DMSO, final concentration 500 µM) or 5 µL DMSO for the control sample set. Both conditions were prepared in triplicates and the solutions were incubated at room temperature for 4 hours. Afterwards, 400 µL of 8 M urea was added to each replicate. A total of 100 μL of mixed hydrophobic and hydrophilic carboxylate-coated magnetic beads (1:1) was washed thrice with 500 μL of water. The reaction mixture was directly transferred onto the equilibrated carboxylate-coated magnetic beads, resuspended, and 600 μL of ethanol was added. After resuspending the beads via vortexing, the suspension was incubated for 5 min at rt and 950 rpm. The beads were washed thrice with 500 μL of 80% ethanol in water using a magnetic rack and the proteins were separately eluted by the addition of 0.5 mL of 0.2% SDS in PBS. For this, the beads were resuspended, incubated for 5 min at 950 rpm, rt, and the supernatant was directly transferred onto 50 μL of equilibrated streptavidin-coated magnetic beads (three times prewashed with 500 μL of 0.2% SDS in PBS). The procedure was repeated once and the supernatants were combined and incubated for 1 h, rt and 950 rpm for biotin/streptavidin binding. The streptavidin-coated magnetic bead mixture was washed thrice with 500 μL of 0.1% NP-40 in PBS, twice with 500 μL of 6 M urea, and thrice with 500 μL of water. Washed bead mixtures were resuspended in 80 μL of 125 mM ammonium bicarbonate (ABC) buffer, and proteins were reduced and alkylated by the addition of 10 μL of 100 mM TCEP and 10 μL of 400 mM CAA, followed by 5 min incubation at 95 °C. Proteins were digested overnight at 37 °C with 1.5 μL of sequencing grade trypsin (0.5 mg/mL). For the workflow in HEK293T cell lysate, the total protein amount and subsequently all reagents used were doubled. The following day, the beads were washed with 40 μL of 100 mM ABC buffer and the supernatants were combined and acidified with 2 μL of formic acid. Tryptic peptides were eluted from the magnetic beads using a magnetic rack, samples were diluted and analyzed by qToF for purity i.e. absence of detergents and successful trypsin digestion. Pure samples were transferred to MS vials and typically 1-2 µL was injected into a Thermo Scientific Dionex UltiMate 3000 system and the same procedure as described above in 2.11.2 was followed for data acquisition.

Raw data files were analyzed with FragPipe version 22.0 and the default LFQ-MBR workflow. ^[10]^ Data were searched against a database containing the human proteome with one protein per gene retrieved from the UniProt. Fragment tolerance was set to 0.5 Da. Analyzed files were further processed using FragPipe-Analyst ^[14]^ as follows: Proteins have to be quantified in >66% of the files for at least one condition and in >66% of the files globally. Median normalization was enabled and imputation disabled so that only experimentally observed intensities were used. For statistical testing, log₂-transformed MaxLFQ intensities were compared using a two-sample t-test statistic with empirical Bayes moderation of the variance estimates.^[15]^ The following significance cut offs were used: log2 fold-change >2.0; -log10 p-value >1.3 (corresponding to p < 0.05).

### **Proteomic profiling of SM-PO**

The proteomic profiling workflow of **SM-PO** in MCF-7 lysate follows the exact same procedure outlined for **bio-PO** in section 3.11.2, using 500 µM of **SM-PO**. Quantitative proteomic analysis for protein abundance in MCF-7 cell lysate can be found in 3.11.1.

# **Supplementary References**

[1] J. Calvert-Evers, K. Hammond, The influence of lysis buffer composition on the expression and activity of protein tyrosine phosphatase, *Electrophoresis* **2000**, *21*, 2944-2946.

[2] R. L. M. van Montfort, M. Congreve, D. Tisi, R. Carr, H. Jhoti, Oxidation state of the active-site cysteine in protein tyrosine phosphatase 1B, *Nature* **2003**, *423*, 773-777.

[3] S. Kumar, B. Zhou, F. Liang, W.-Q. Wang, Z. Huang, Z.-Y. Zhang, Activity-based probes for protein tyrosine phosphatases, *Proc. Natl. Acad. Sci. U.S.A.* **2004**, *101*, 7943-7948.

[4] M. S. Choy, Y. Li, L. Machado, M. B. A. Kunze, C. R. Connors, X. Wei, K. Lindorff-Larsen, R. Page, W. Peti, Conformational Rigidity and Protein Dynamics at Distinct Timescales Regulate PTP1B Activity and Allostery, *Mol. Cell* **2017**, *65*, 644-658.e645.

[5] M. A. Kasper, A. Stengl, P. Ochtrop, M. Gerlach, T. Stoschek, D. Schumacher, J. Helma, M. Penkert, E. Krause, H. Leonhardt, C. P. R. Hackenberger, Ethynylphosphonamidates for the Rapid and Cysteine-Selective Generation of Efficacious Antibody-Drug Conjugates, *Angew. Chem. Int. Ed.* **2019**, *58*, 11631-11636.

[6] D. S. Munasinghe, M.-A. Kasper, R. Jasiński, K. Kula, M. Palusiak, M. Celeda, G. Mlostoń, C. P. R. Hackenberger, (3+2)-Cyclization Reactions of Unsaturated Phosphonites with Aldehydes and Thioketones, *Chem. Eur. J.* **2023**, *29*, e202300806.

[7] M.-A. Kasper, M. Glanz, A. Stengl, M. Penkert, S. Klenk, T. Sauer, D. Schumacher, J. Helma, E. Krause, M. C. Cardoso, H. Leonhardt, C. P. R. Hackenberger, Cysteine-Selective Phosphonamidate Electrophiles for Modular Protein Bioconjugations, *Angew. Chem. Int. Ed.* **2019**, *58*, 11625-11630.

[8] S. Kumar, B. Zhou, F. Liang, H. Yang, W.-Q. Wang, Z.-Y. Zhang, Global Analysis of Protein Tyrosine Phosphatase Activity with Ultra-Sensitive Fluorescent Probes, *J. Proteome Res.* **2006**, *5*, 1898-1905.

[9] F. Texier-Boullet, A. Foucaud, A Convenient Synthesis of Dialkyl 1-Hydroxyalkanephosphonates using Potassium or Caesium Fluoride without Solvent, *Synthesis* **1982**, *1982*, 165-166.

[10] A. T. Kong, F. V. Leprevost, D. M. Avtonomov, D. Mellacheruvu, A. I. Nesvizhskii, MSFragger: ultrafast and comprehensive peptide identification in mass spectrometry-based proteomics, *Nat. Methods* **2017**, *14*, 513-520.

[11] F. Yu, G. C. Teo, A. T. Kong, S. E. Haynes, D. M. Avtonomov, D. J. Geiszler, A. I. Nesvizhskii, Identification of modified peptides using localization-aware open search, *Nat. Commun.* **2020**, *11*, 4065.

[12] L. Tautz, E. A. Sergienko, High-throughput screening for protein tyrosine phosphatase activity modulators, *Methods Mol. Biol.* **2013**, *1053*, 223-240.

[13] T. Becker, A. Wiest, A. Telek, D. Bejko, A. Hoffmann-Röder, P. Kielkowski, Transforming Chemical Proteomics Enrichment into a High-Throughput Method Using an SP2E Workflow, *JACS Au* **2022**, *2*, 1712-1723.

[14] Y. Hsiao, H. Zhang, G. X. Li, Y. Deng, F. Yu, H. Valipour Kahrood, J. R. Steele, R. B. Schittenhelm, A. I. Nesvizhskii, Analysis and Visualization of Quantitative Proteomics Data Using FragPipe-Analyst, *J. Proteome Res.* **2024**, *23*, 4303-4315.

[15] M. E. Ritchie, B. Phipson, D. Wu, Y. Hu, C. W. Law, W. Shi, G. K. Smyth, limma powers differential expression analyses for RNA-sequencing and microarray studies, *Nucleic Acids Res.*, **2015**, 43, e47.

# **Uncropped gels and western blots**

**Figure 5a**


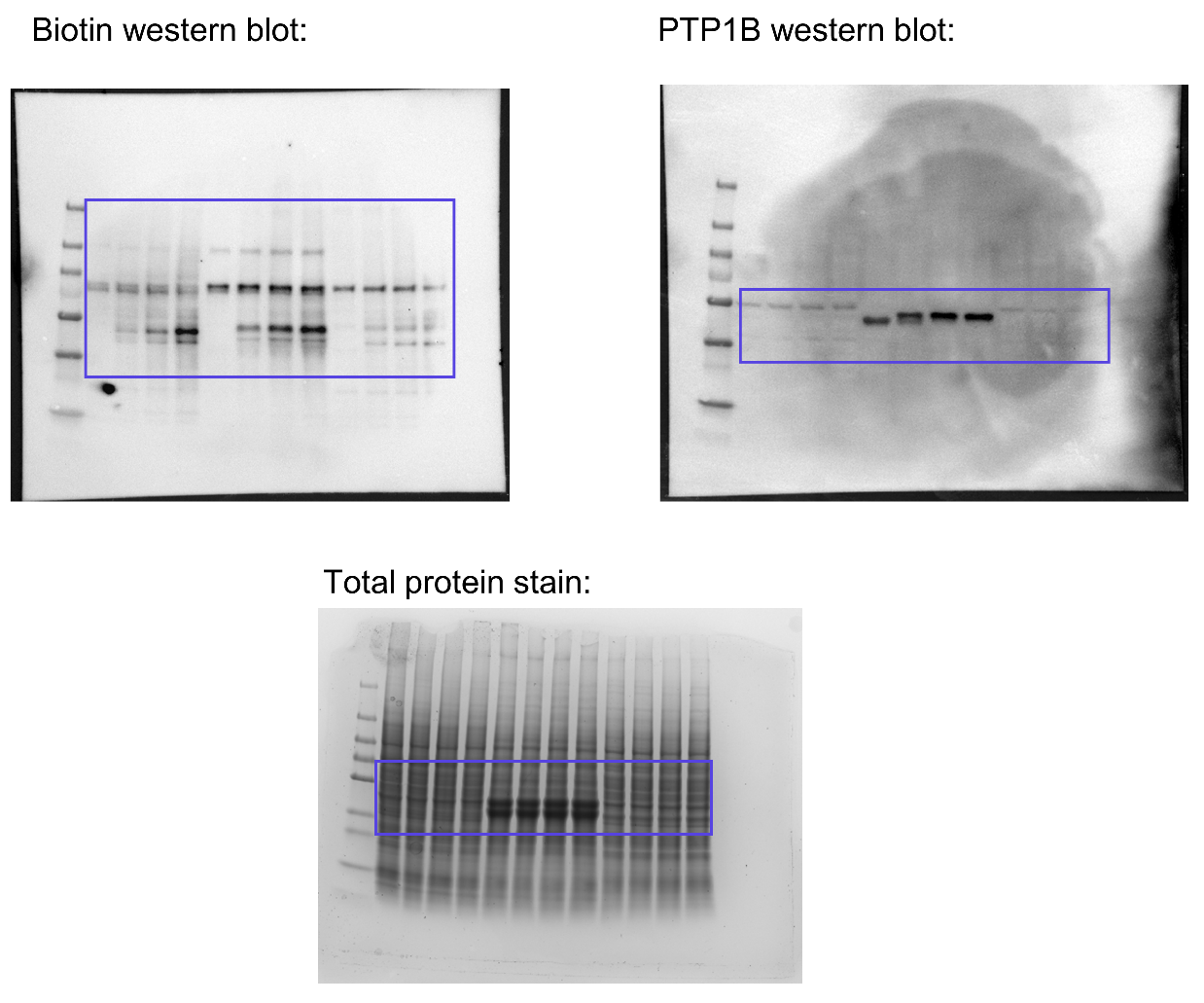


**Figure S7a, b**

**
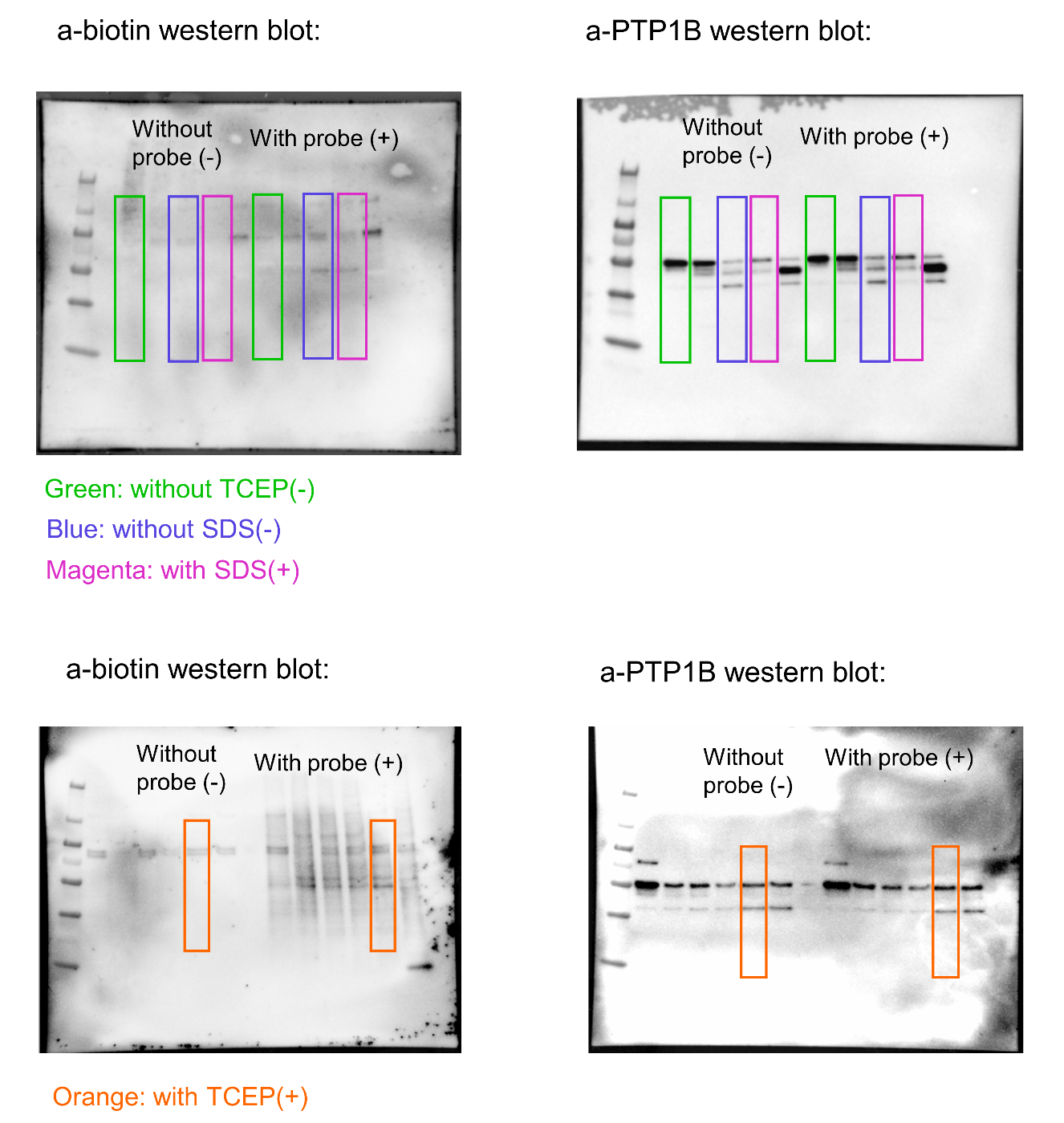
**

**
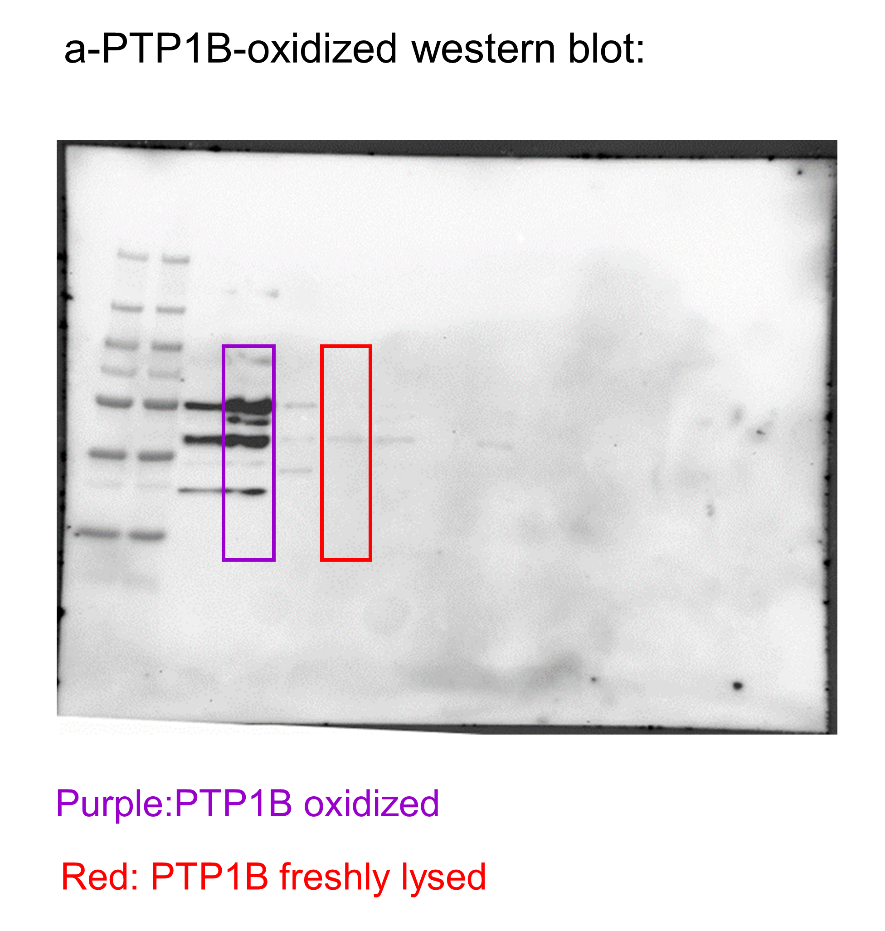
Figure S7c**

**Figure S8**


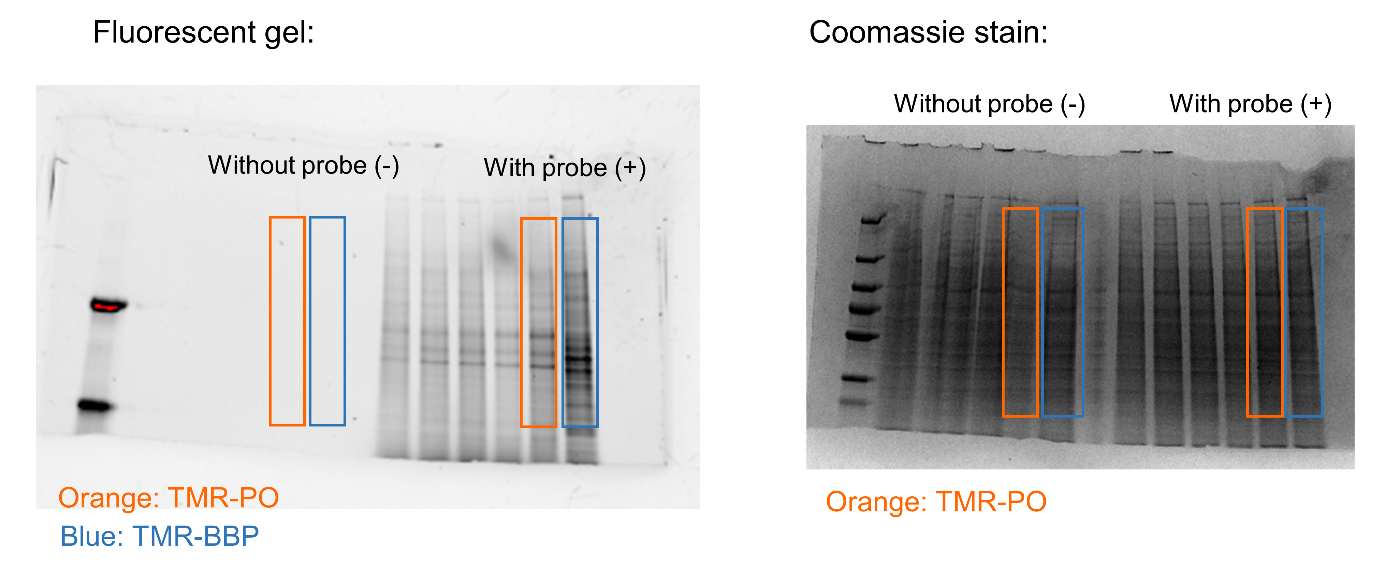


**
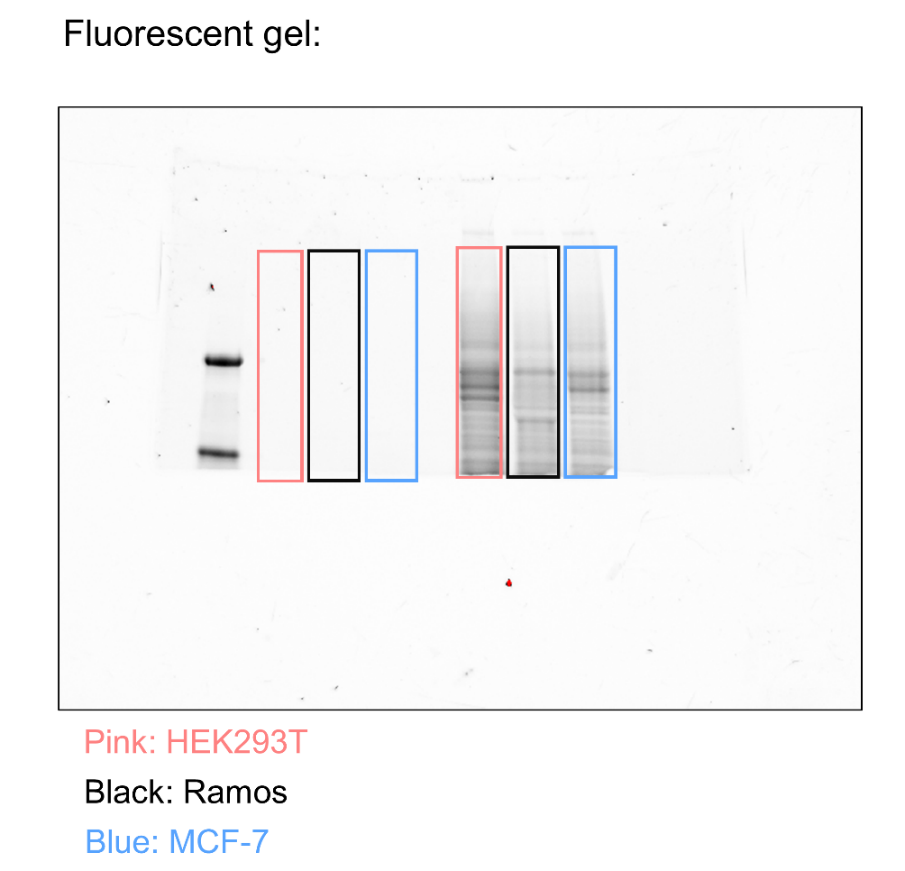
Figure S9**

**Figure S10a**


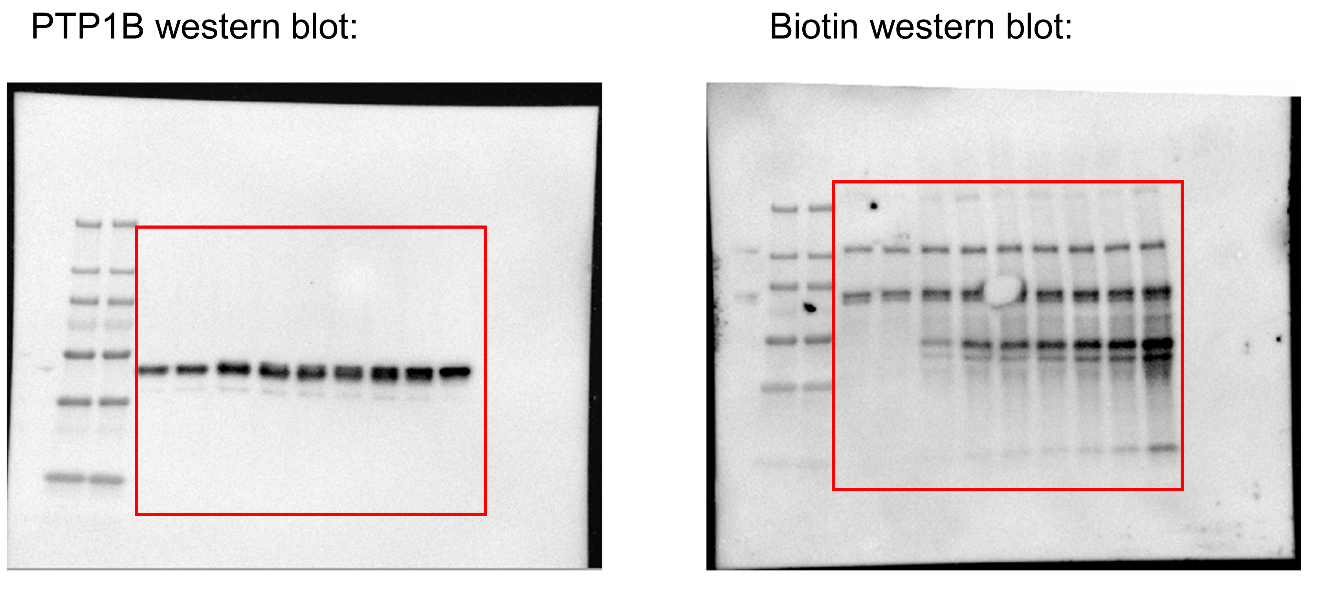


**Figure S10b**


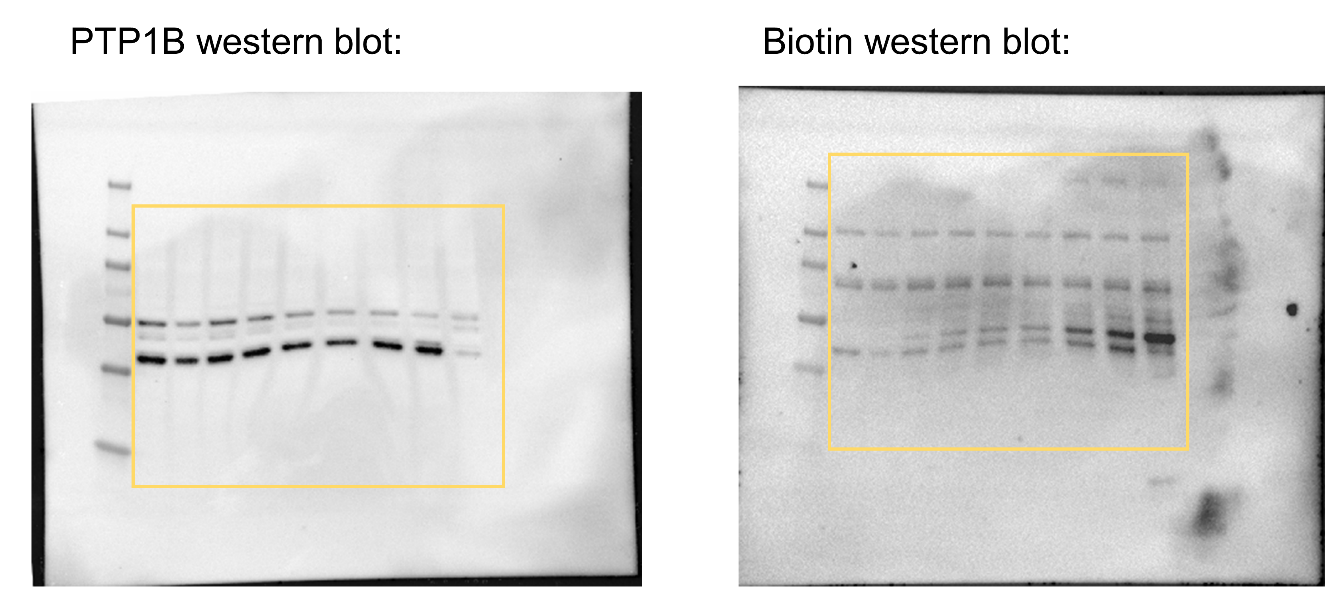


**Figure S10c**


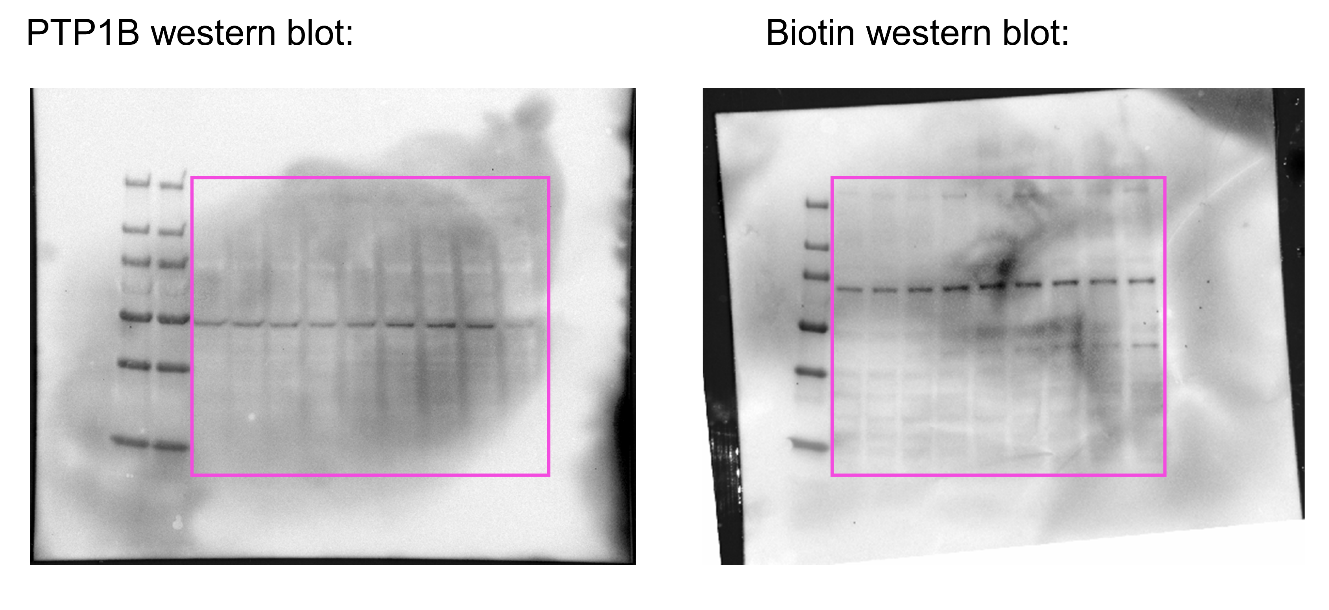


**Figure S11**


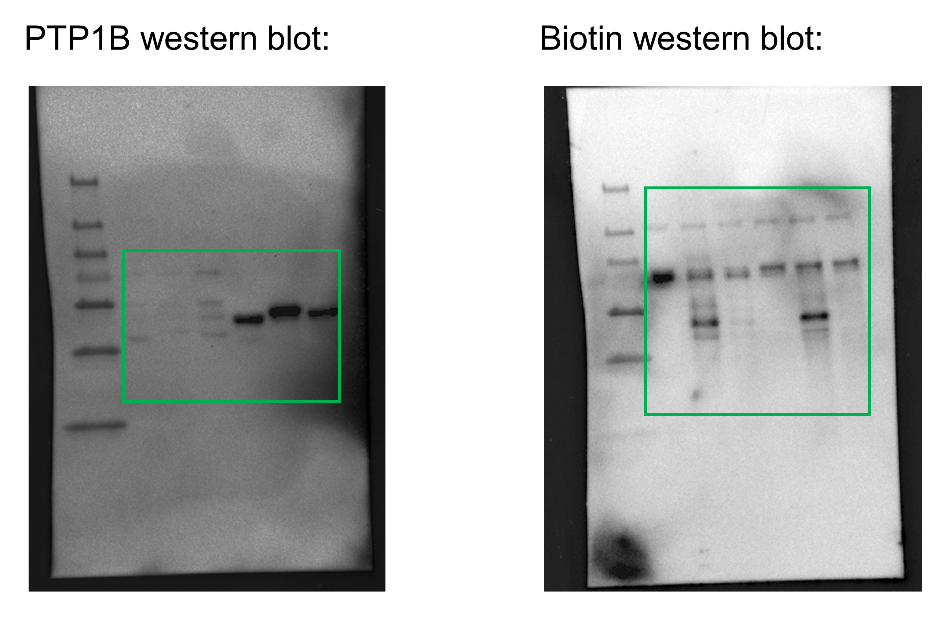


**Figure S12**


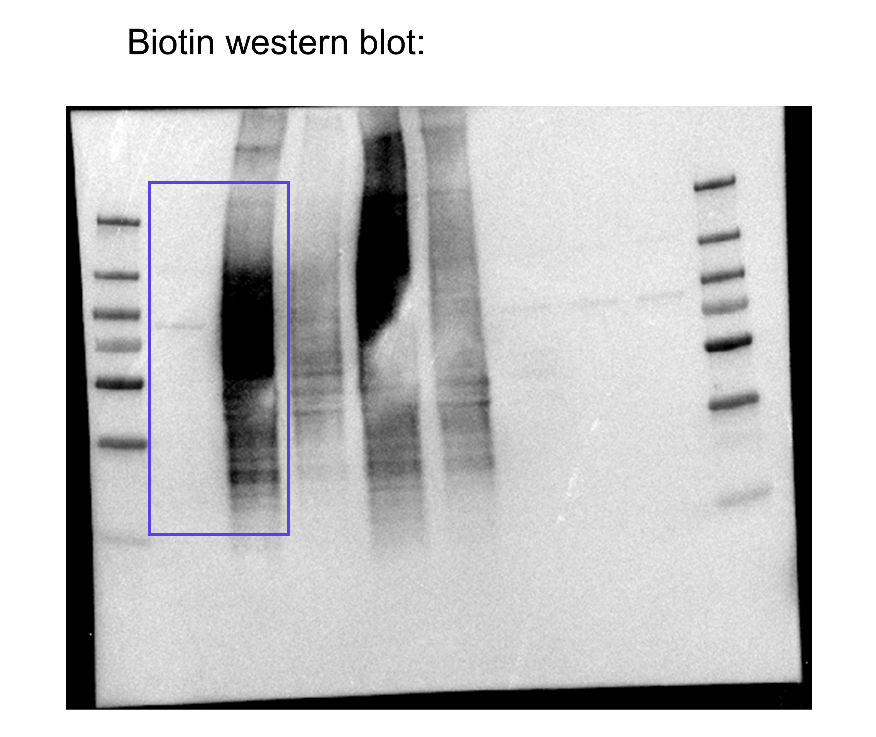


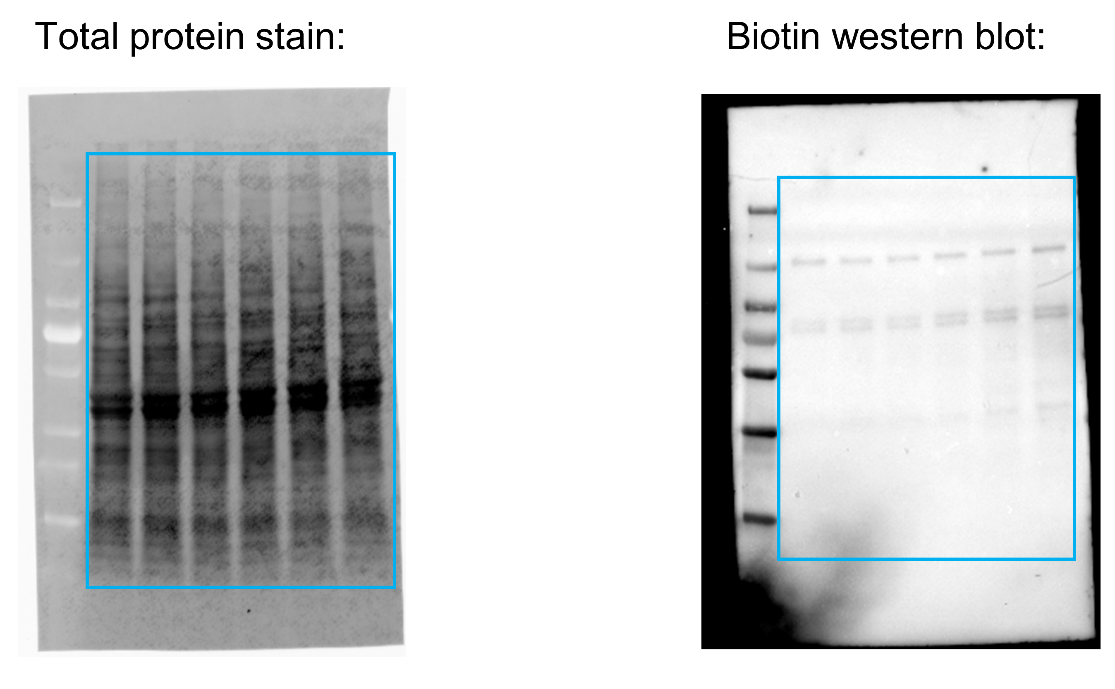
**Figure S13b**

**Figure S13c**


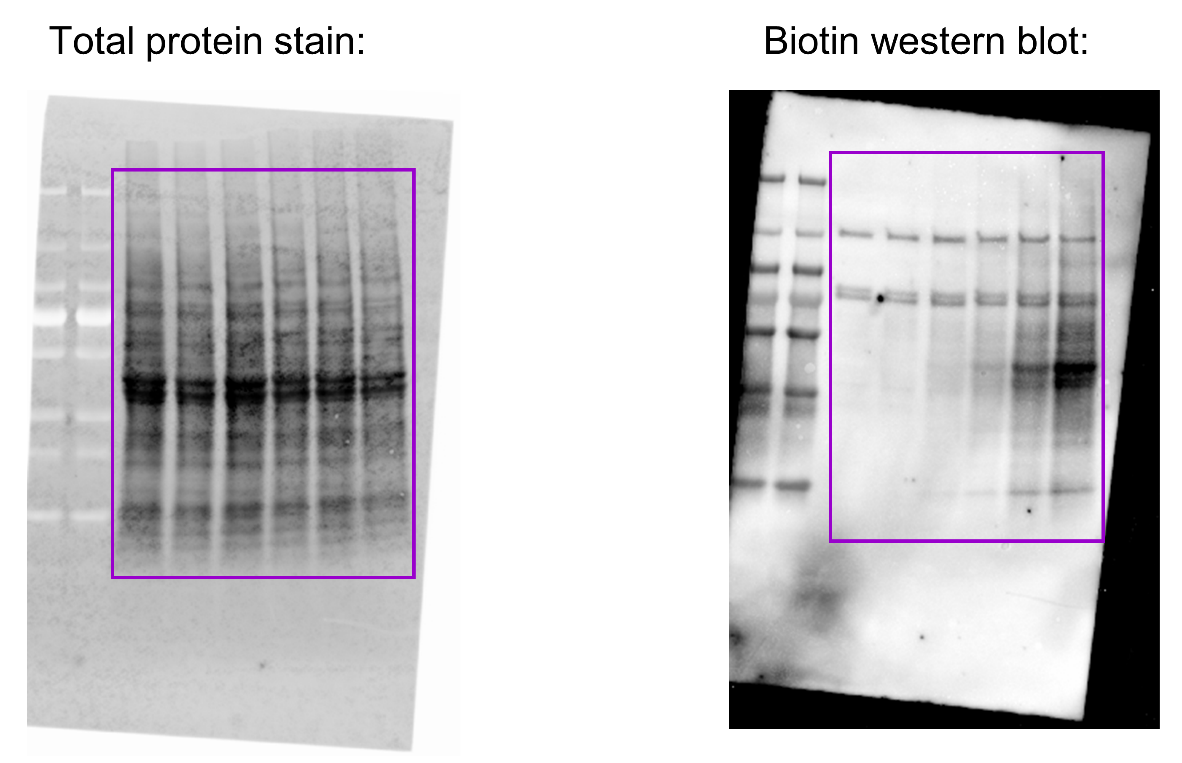


# **NMR Spectra**

Di-(4-acetoxy benzyl) ethynylphosphonite **(1)**


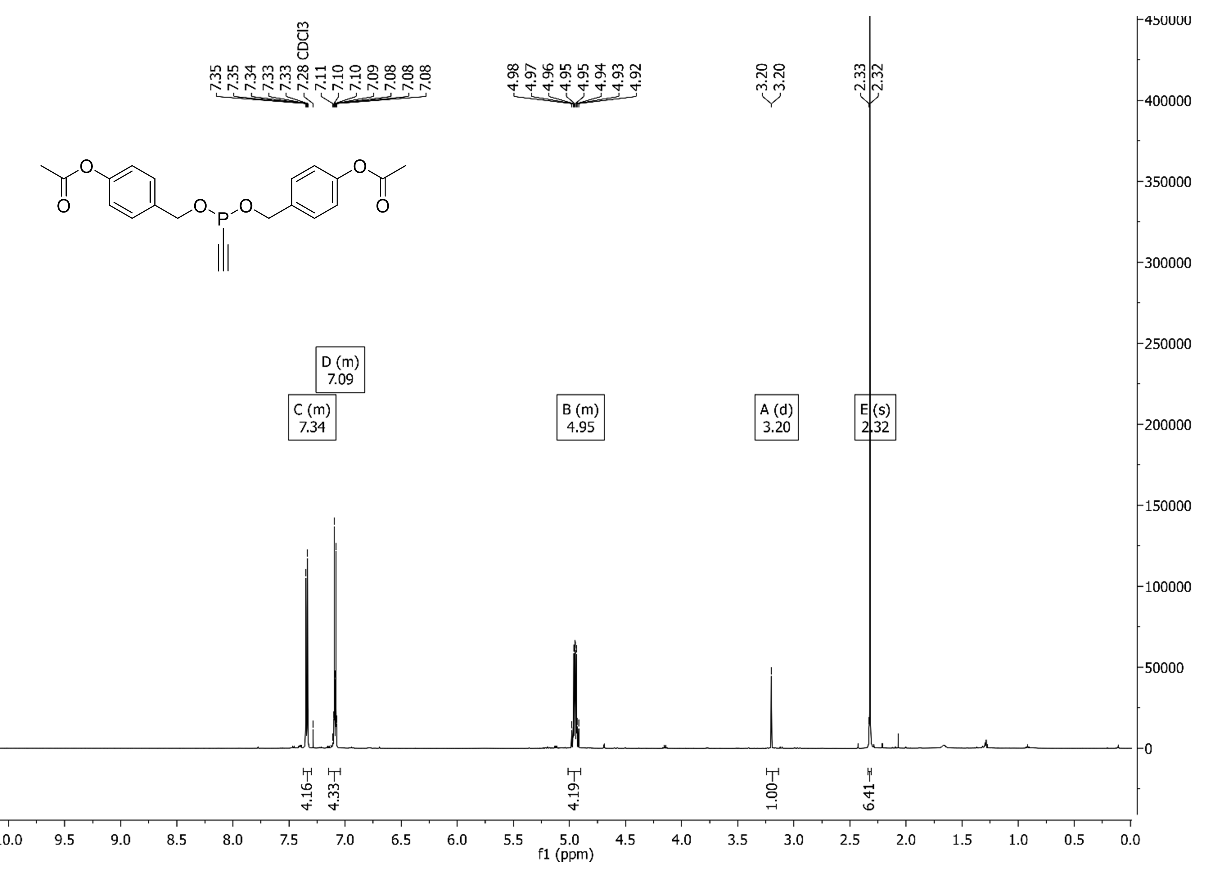


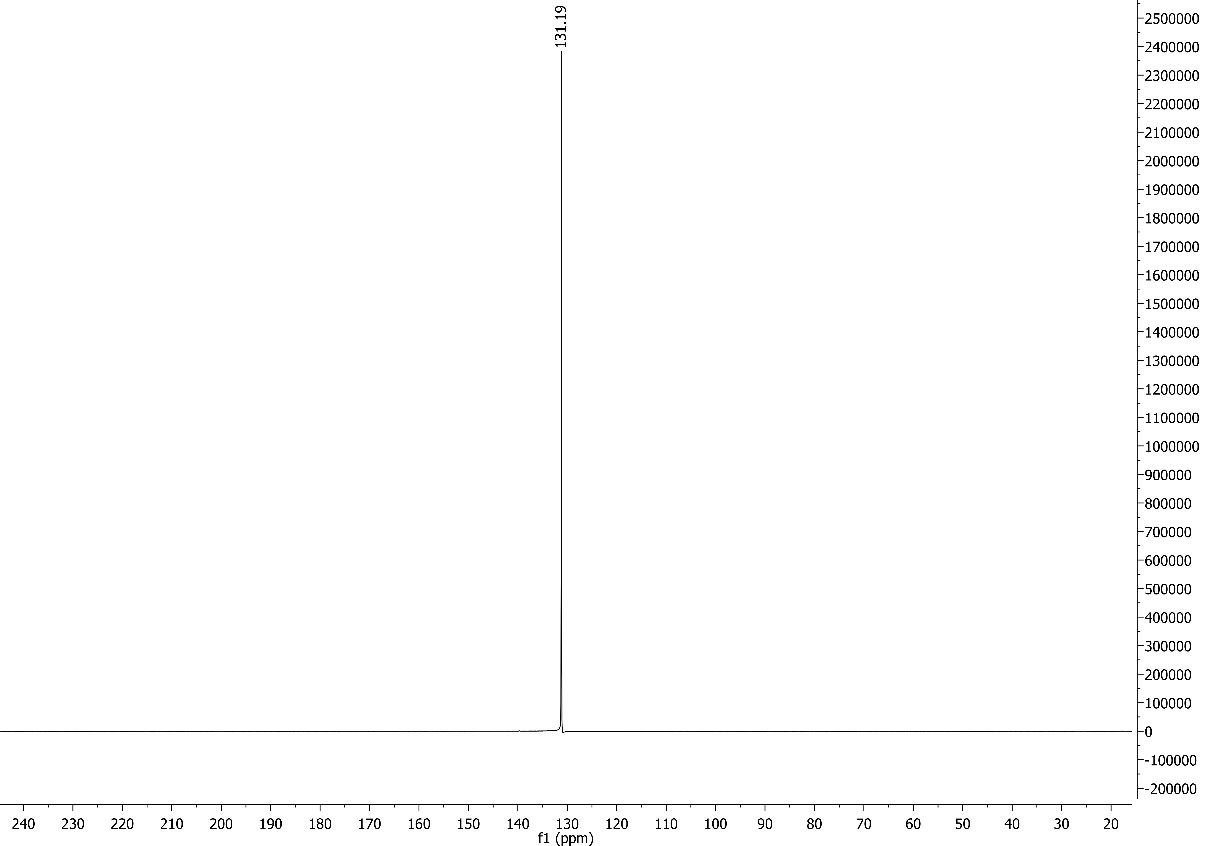


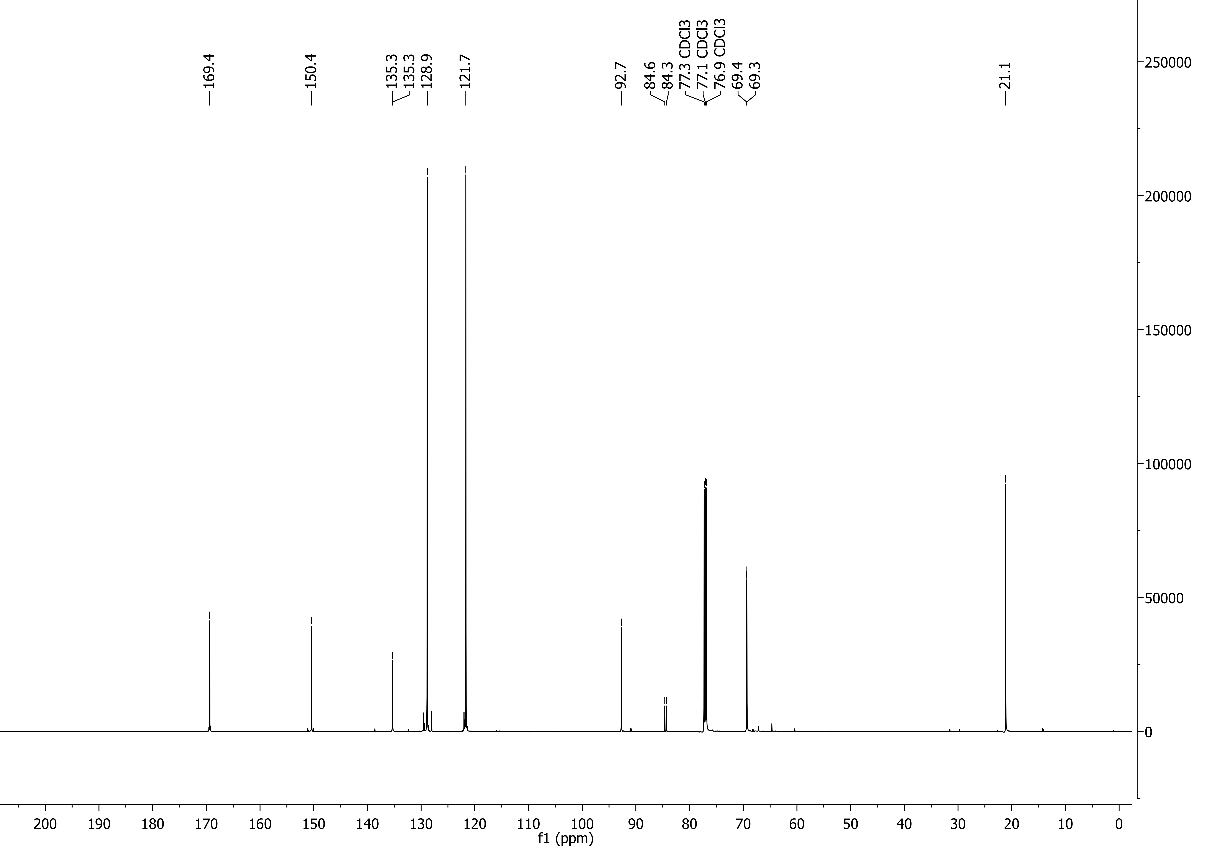


1-(benzyloxy)-1-ethynyl-*N,N*-diisopropylphosphanamine – (crude ^31^P NMR) **(2)**

**
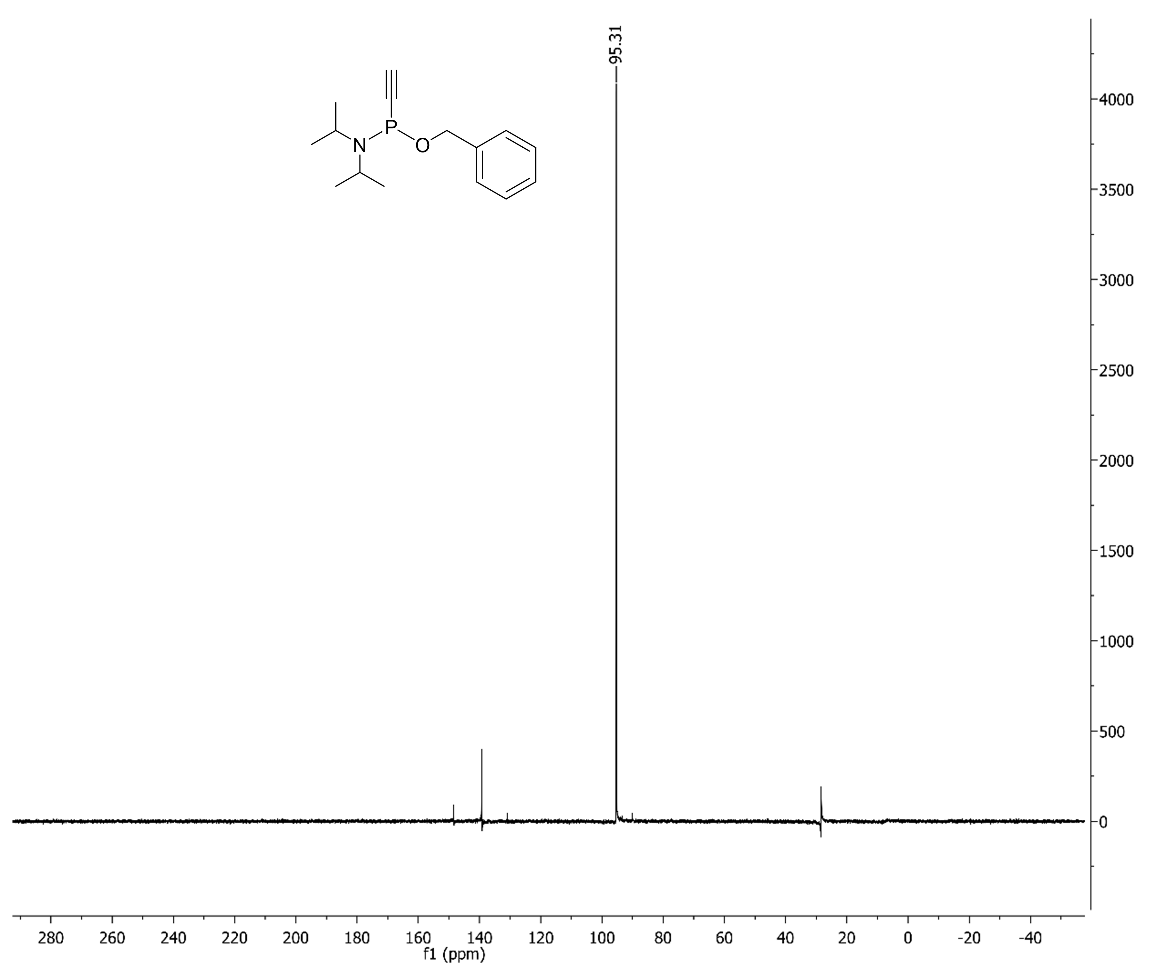
**

**
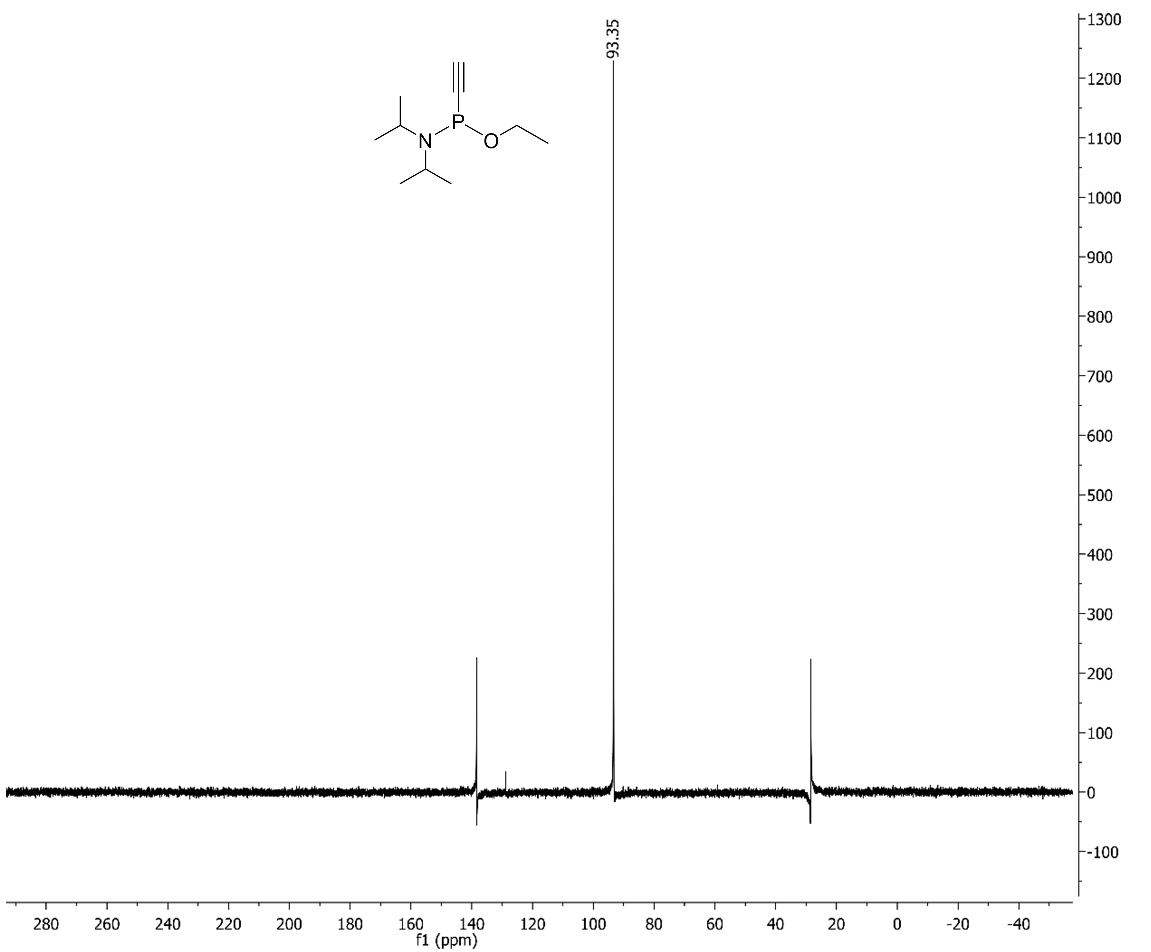
**1-(ethoxy)-1-ethynyl-*N,N*-diisopropylphosphanamine – (crude ^31^P NMR) **(3)**

*N*-(4-azido phenethyl) biotinamide **(4)**


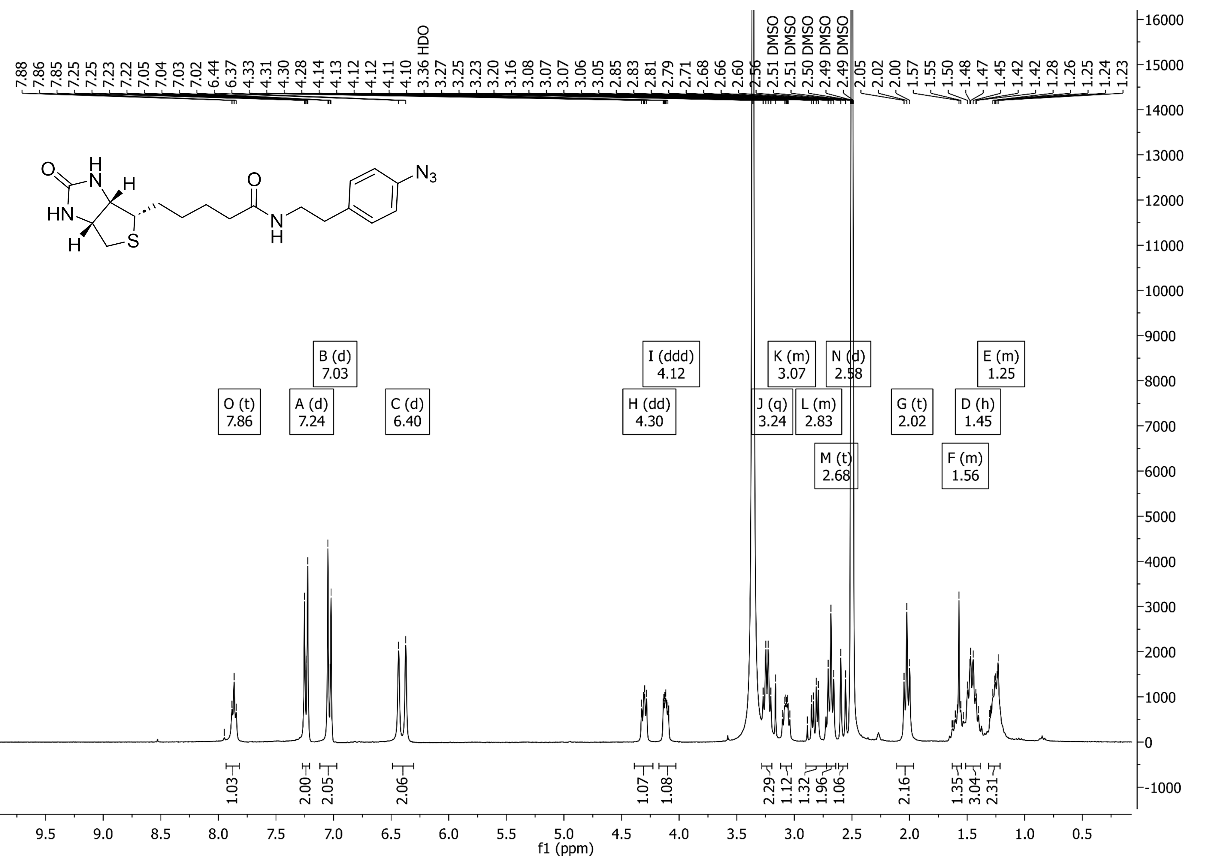


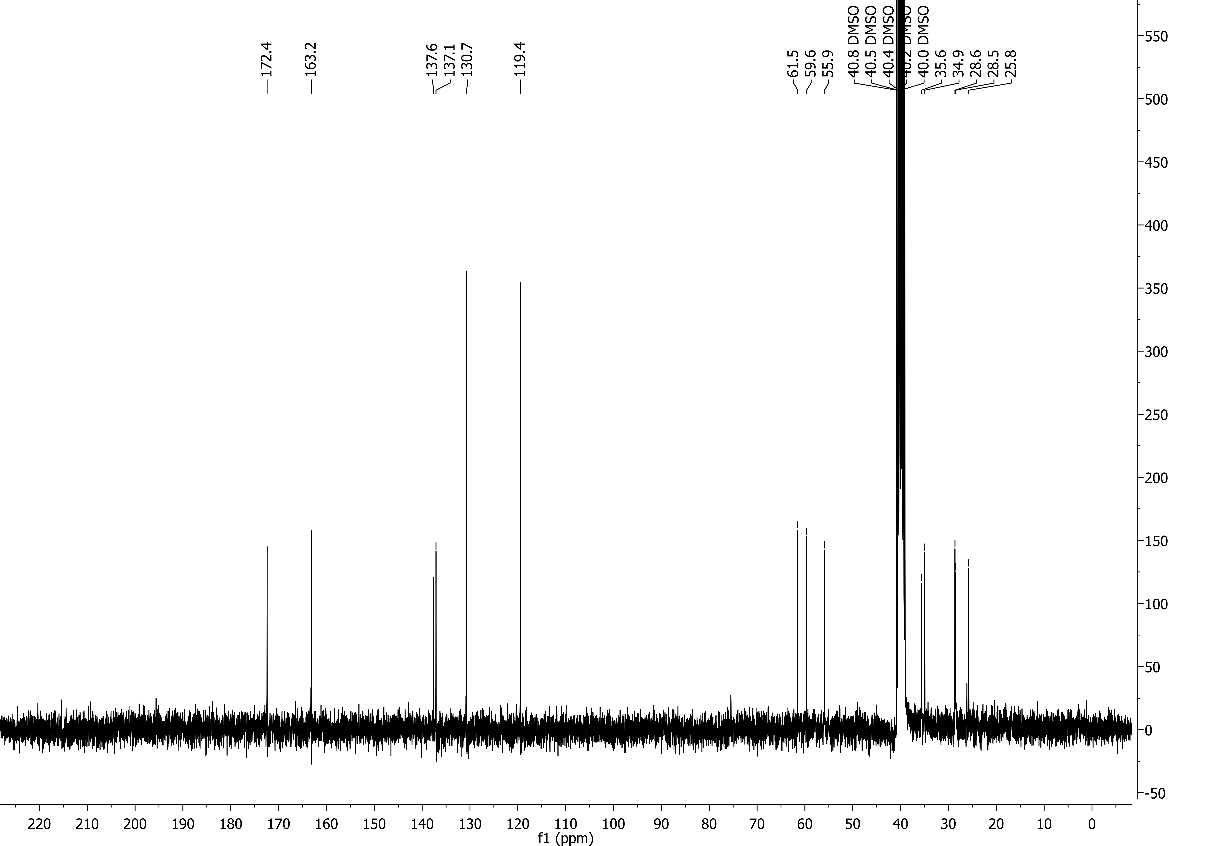


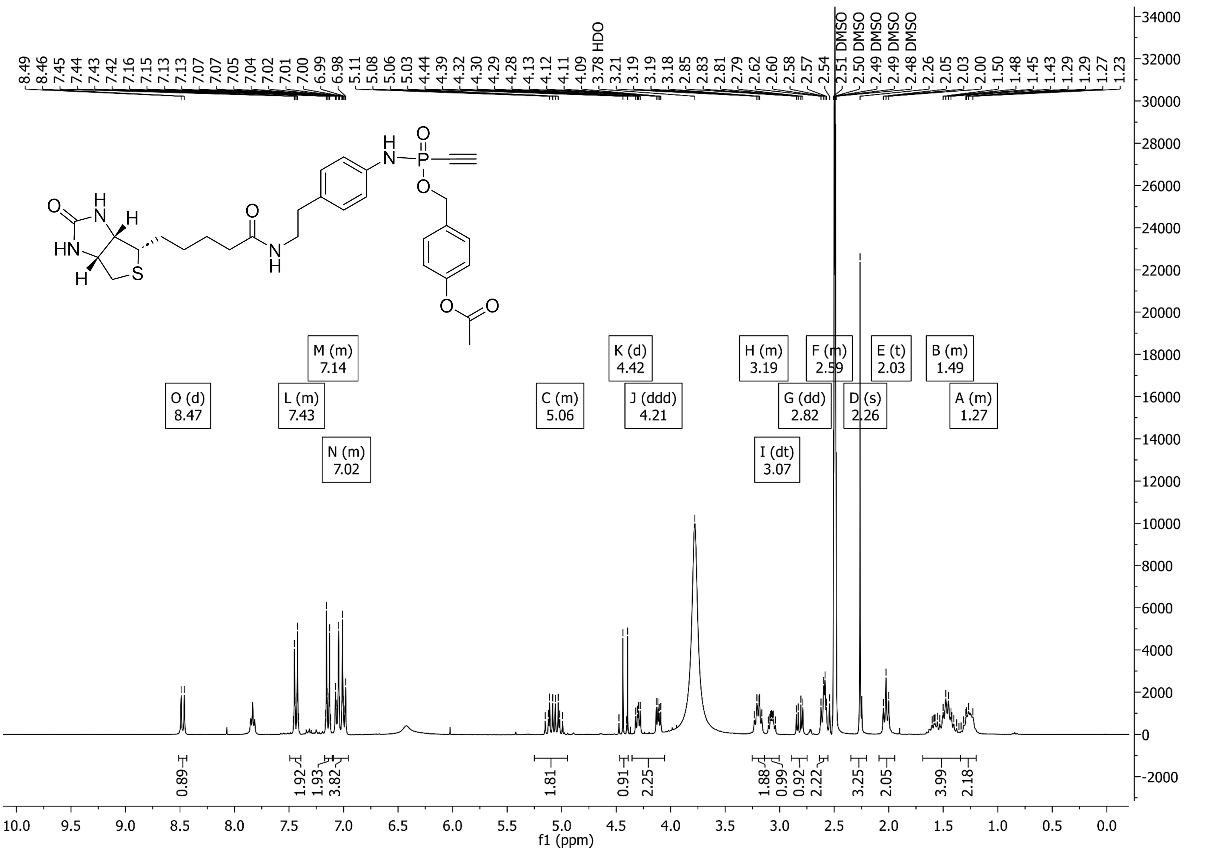
*O*-4-acetoxy-benzyl-*N* -(4-(2-biotinamidoethyl)phenyl)-*P*-ethynylphosphonamidate **(5)**


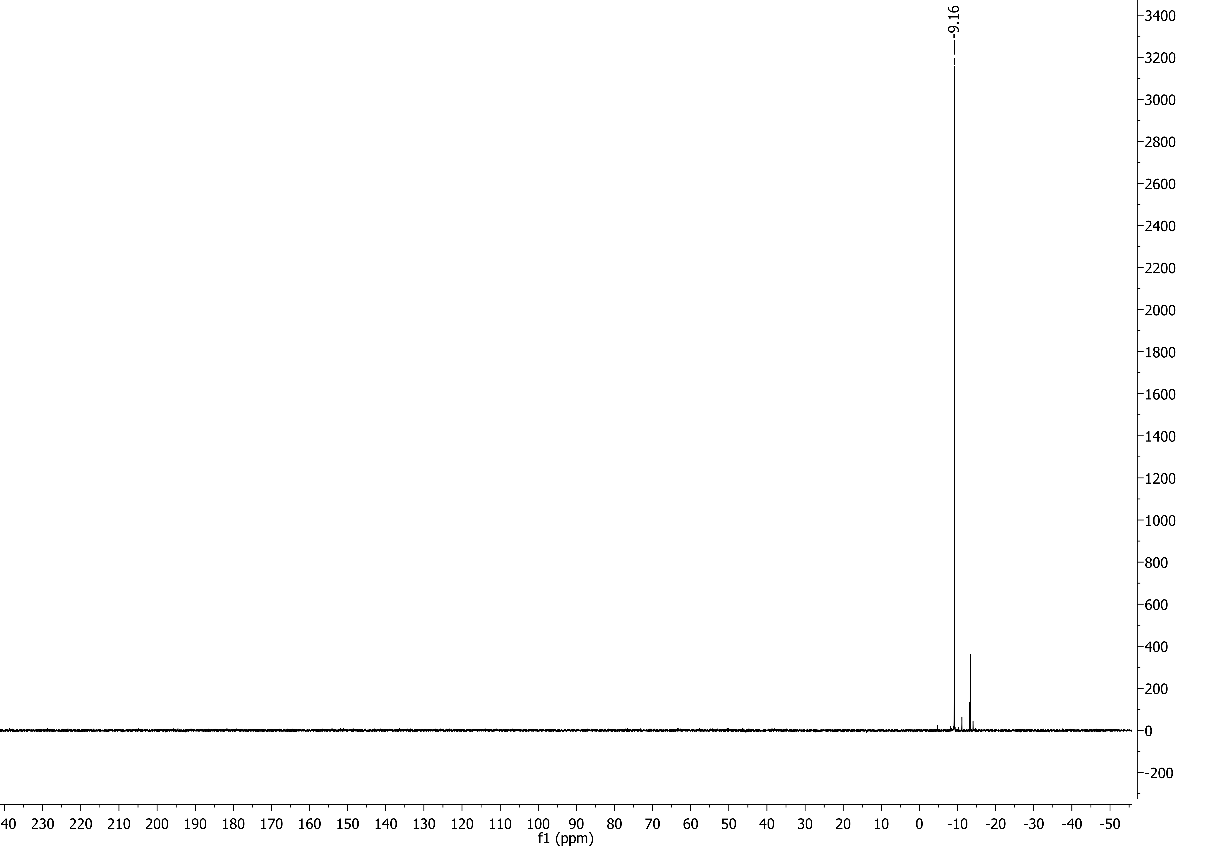


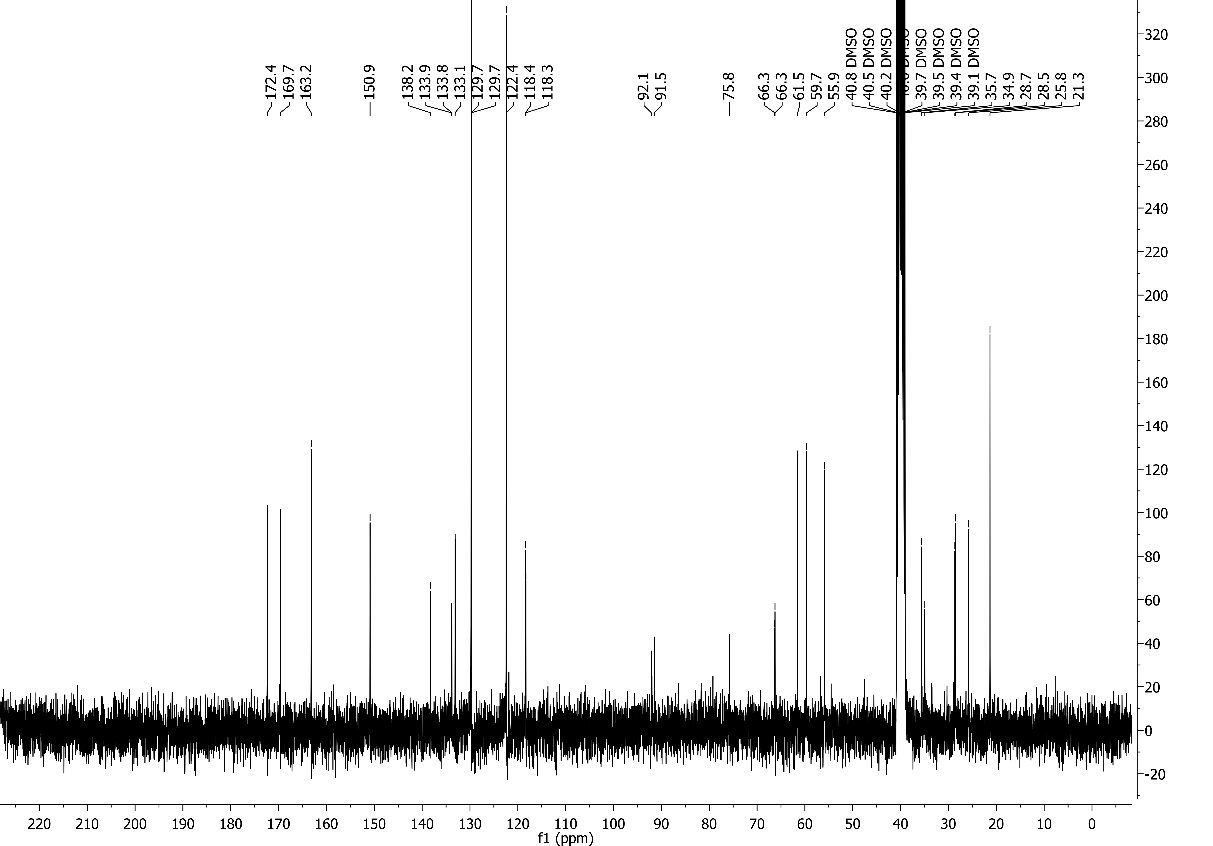


*N*-(4-(2-biotinamidoethyl)phenyl)-*P*-ethynylphosphonamidic acid **(6)**


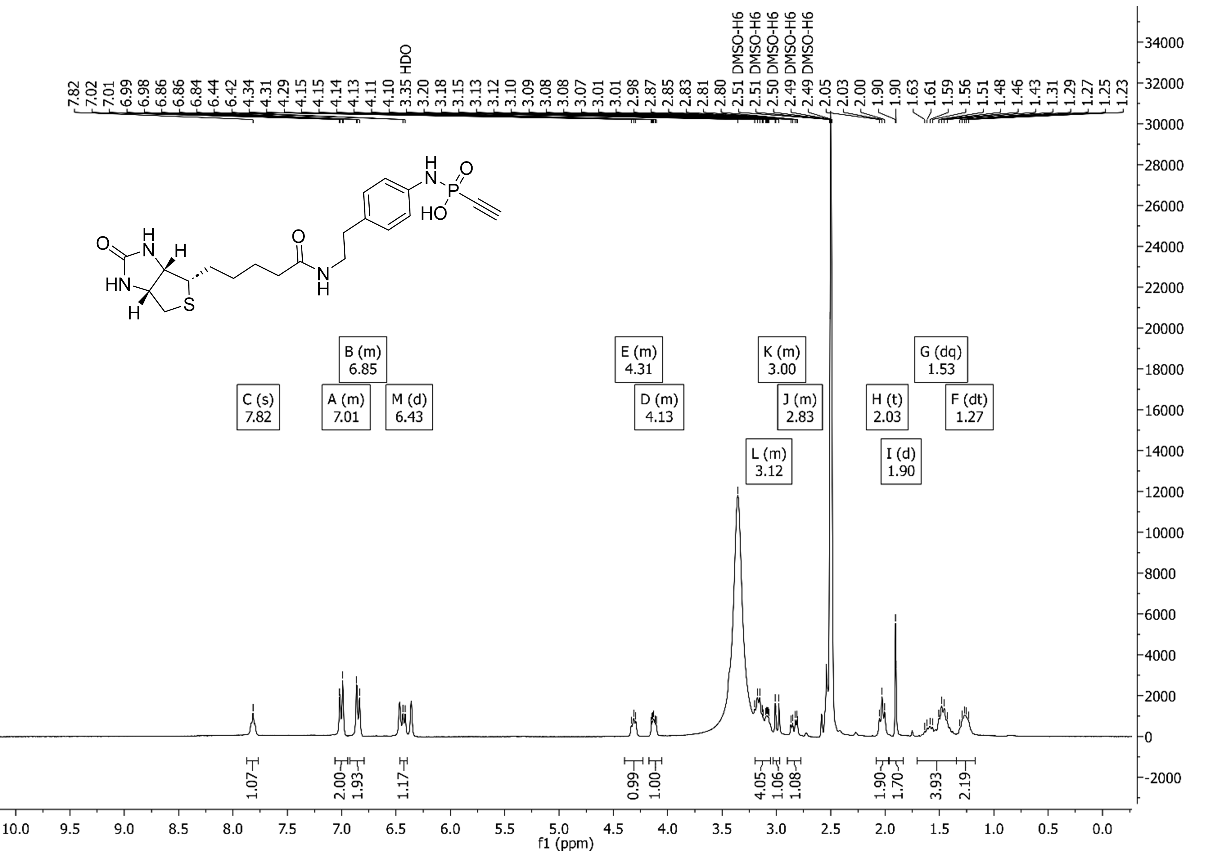


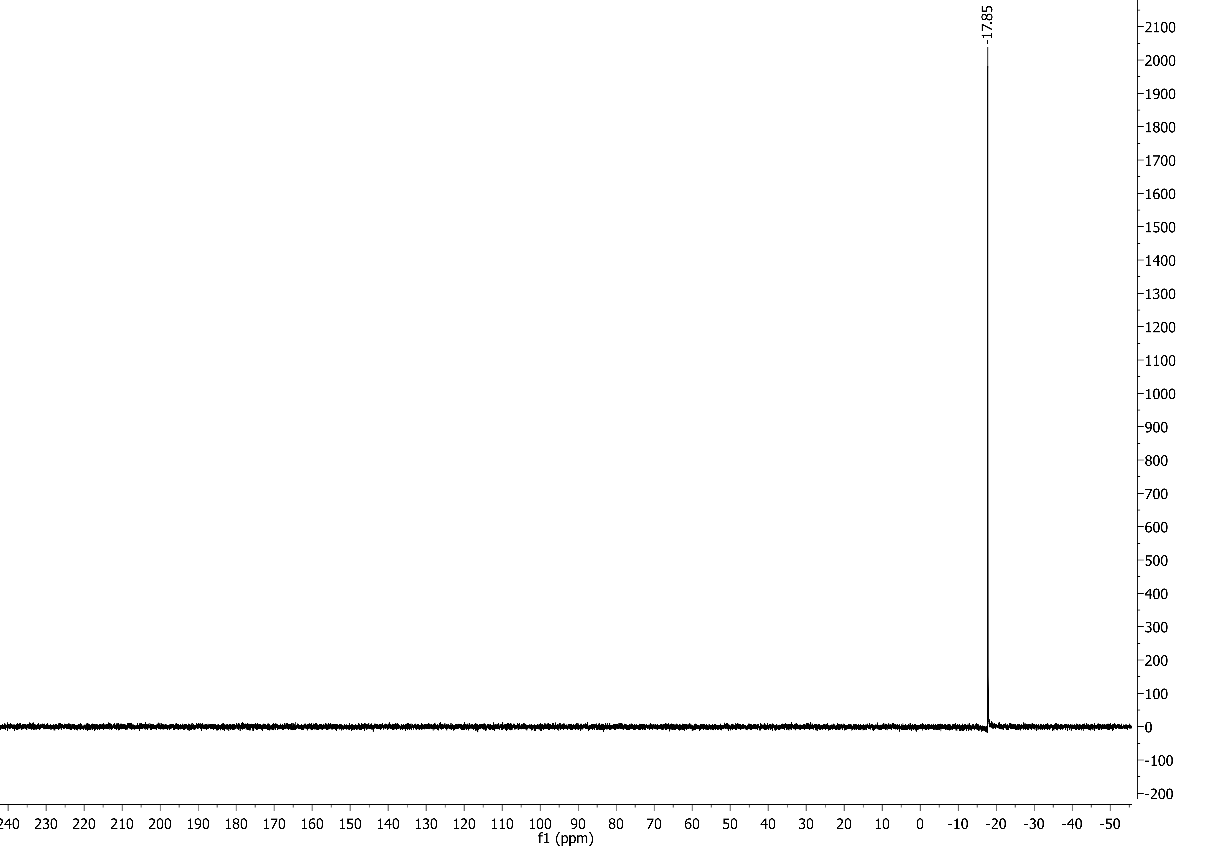


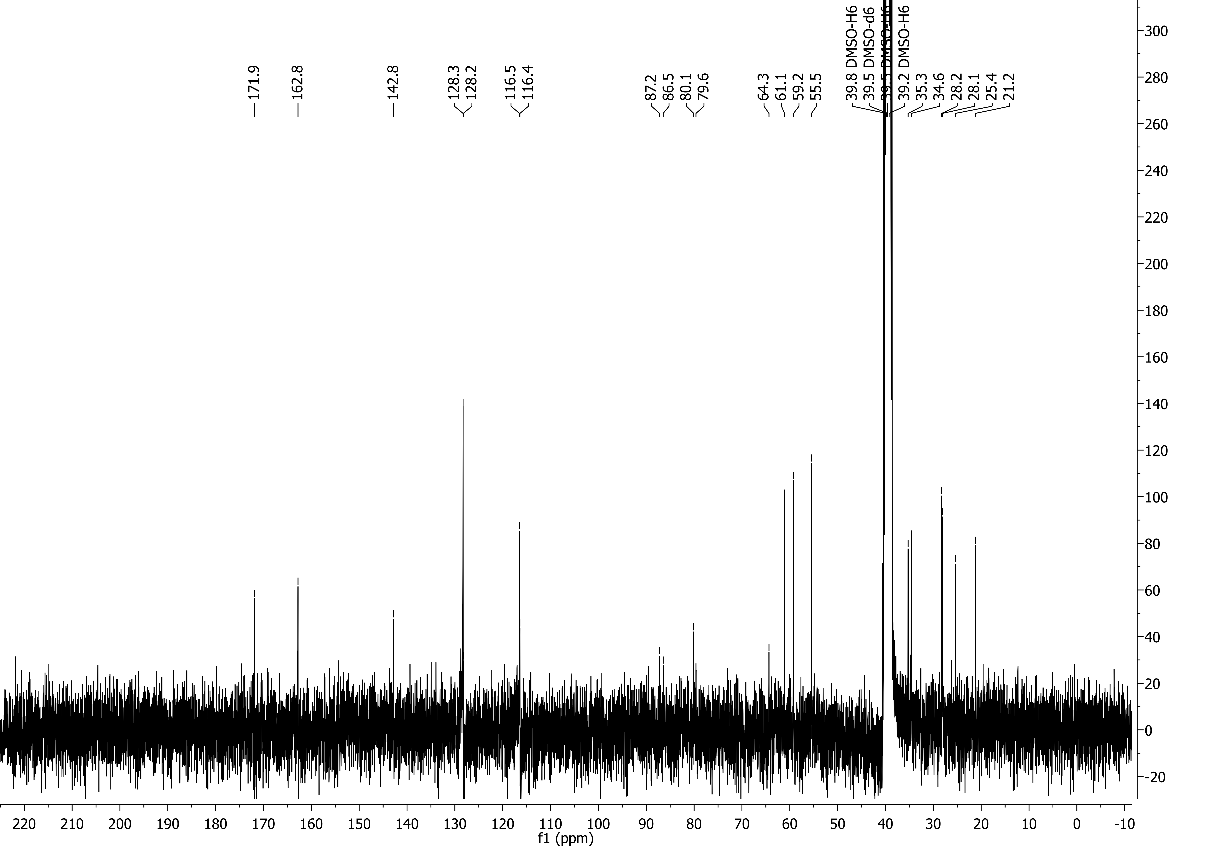


*O*-(4-acetoxybenzyl)-*O*-(*N*-Boc-tyraminyl)-*P*-ethynylphosphonate **(7)**


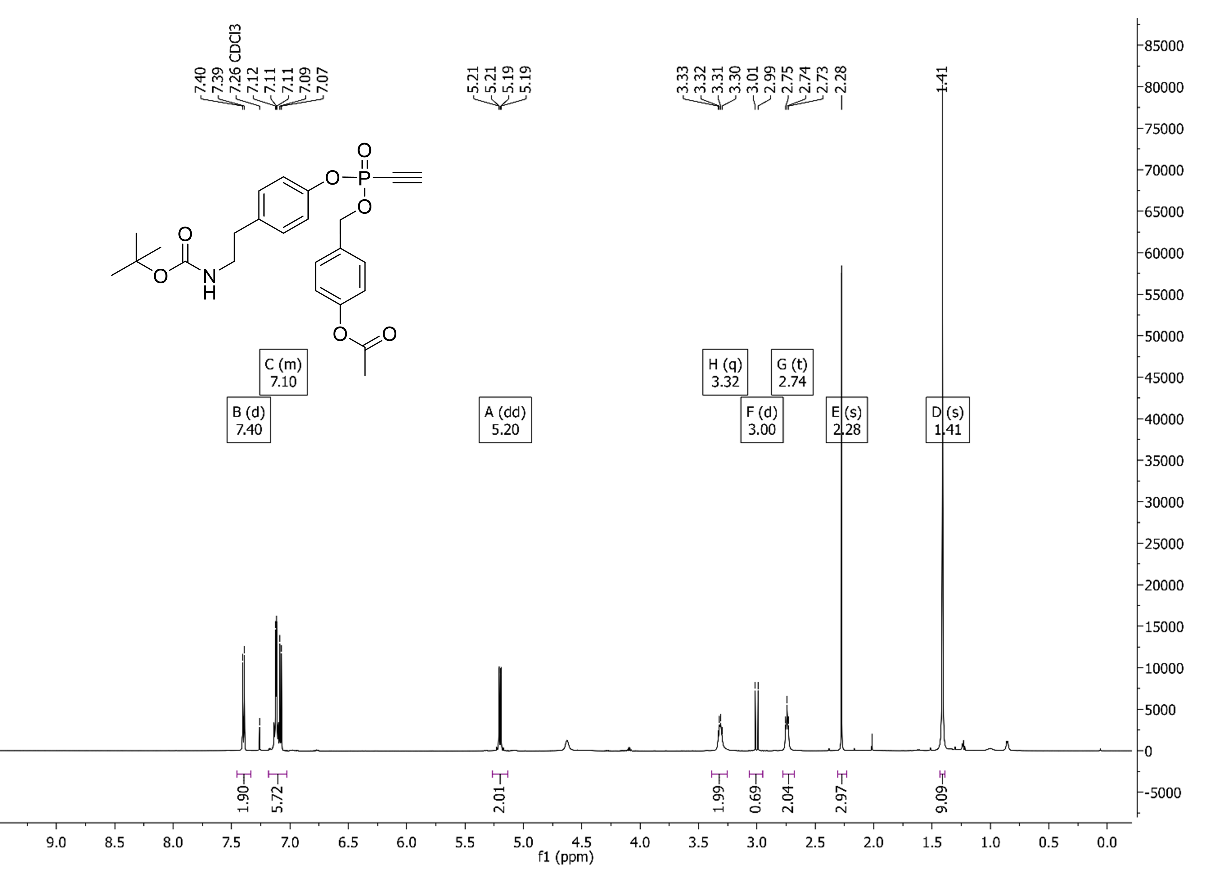


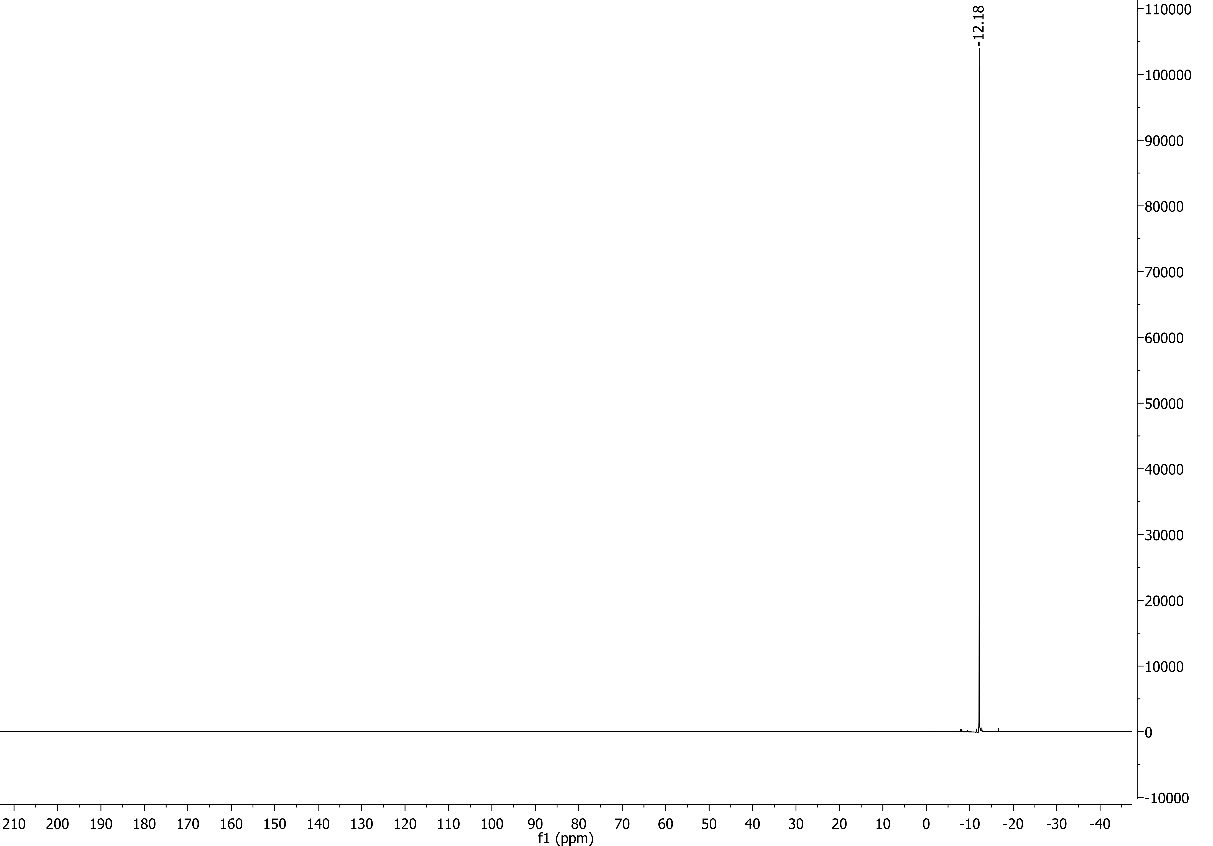


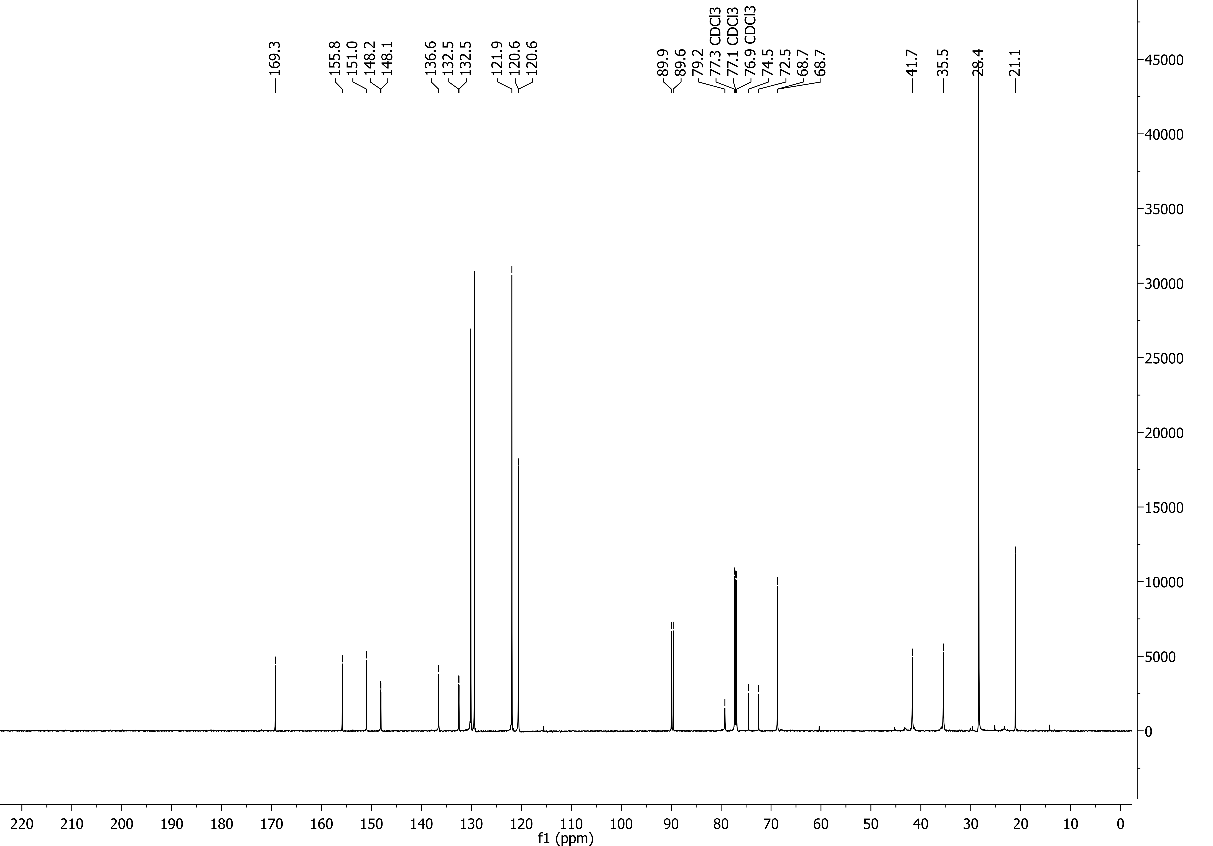


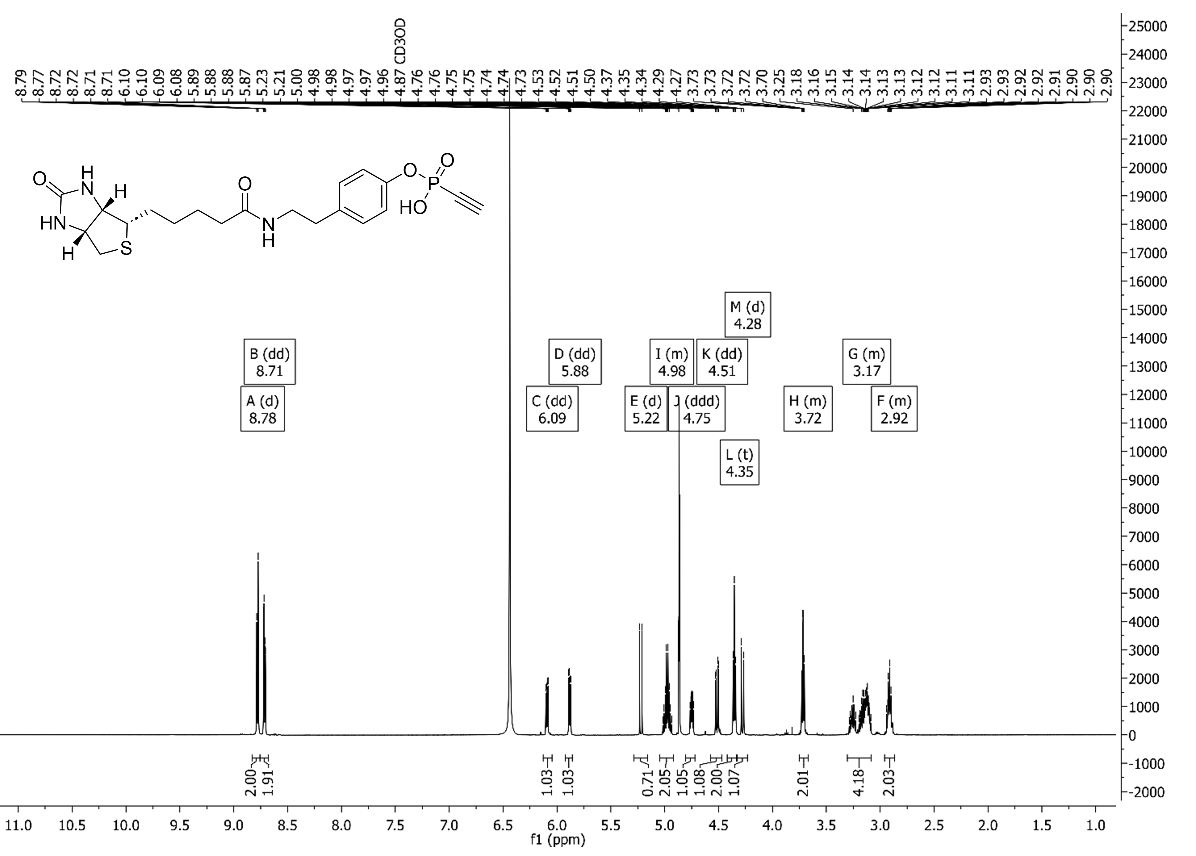
4-(2-biotinamidoethyl)phenyl ethynylphosphonic acid monoester **(9)**


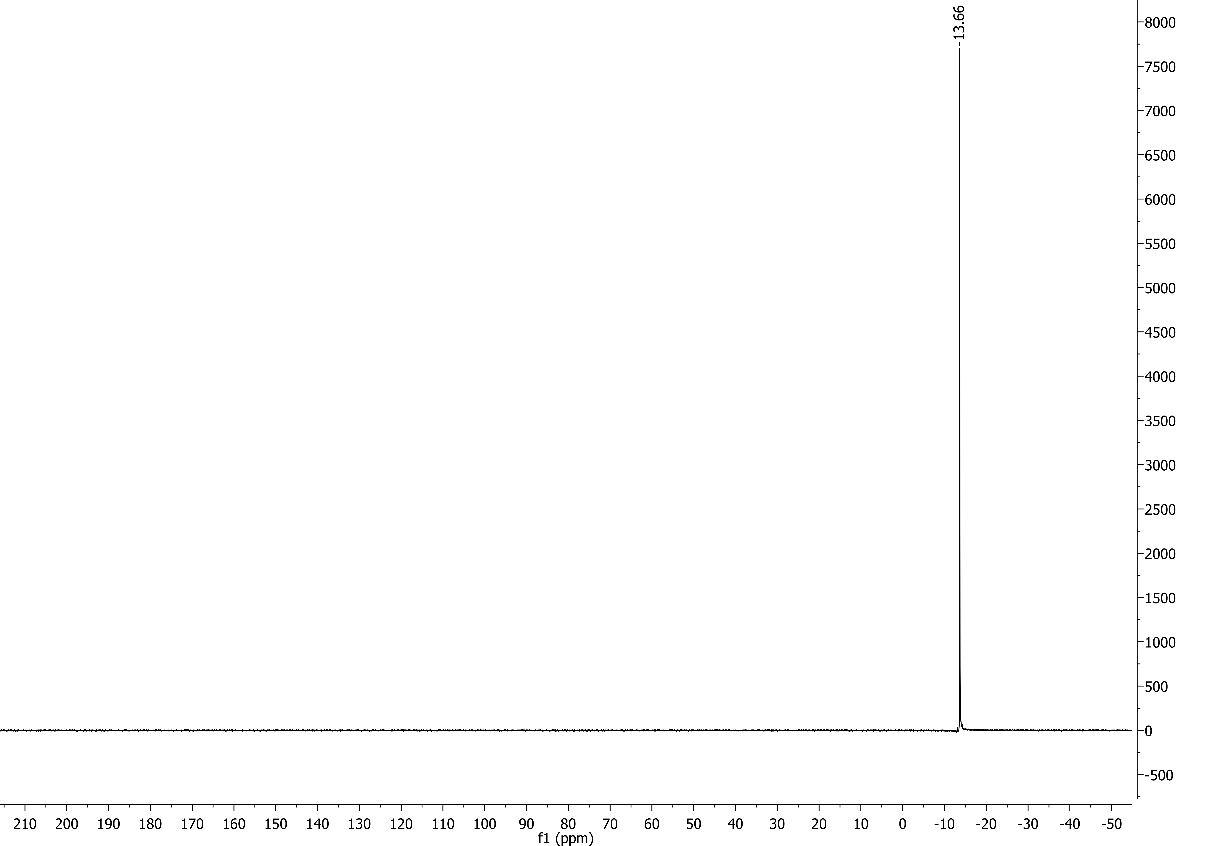


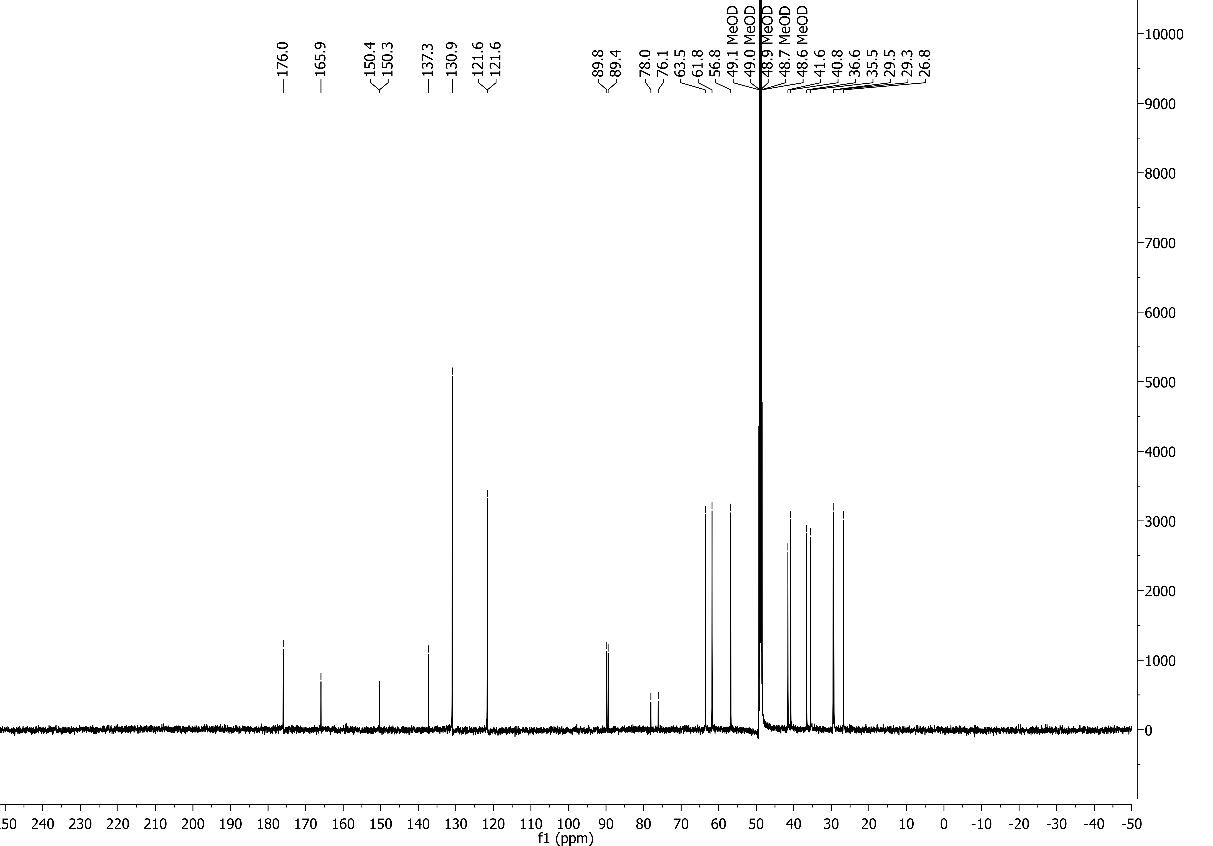


Diethyl ((4-(N-Boc-aminomethyl)phenyl)(hydroxy)methyl)phosphonate **(10)**


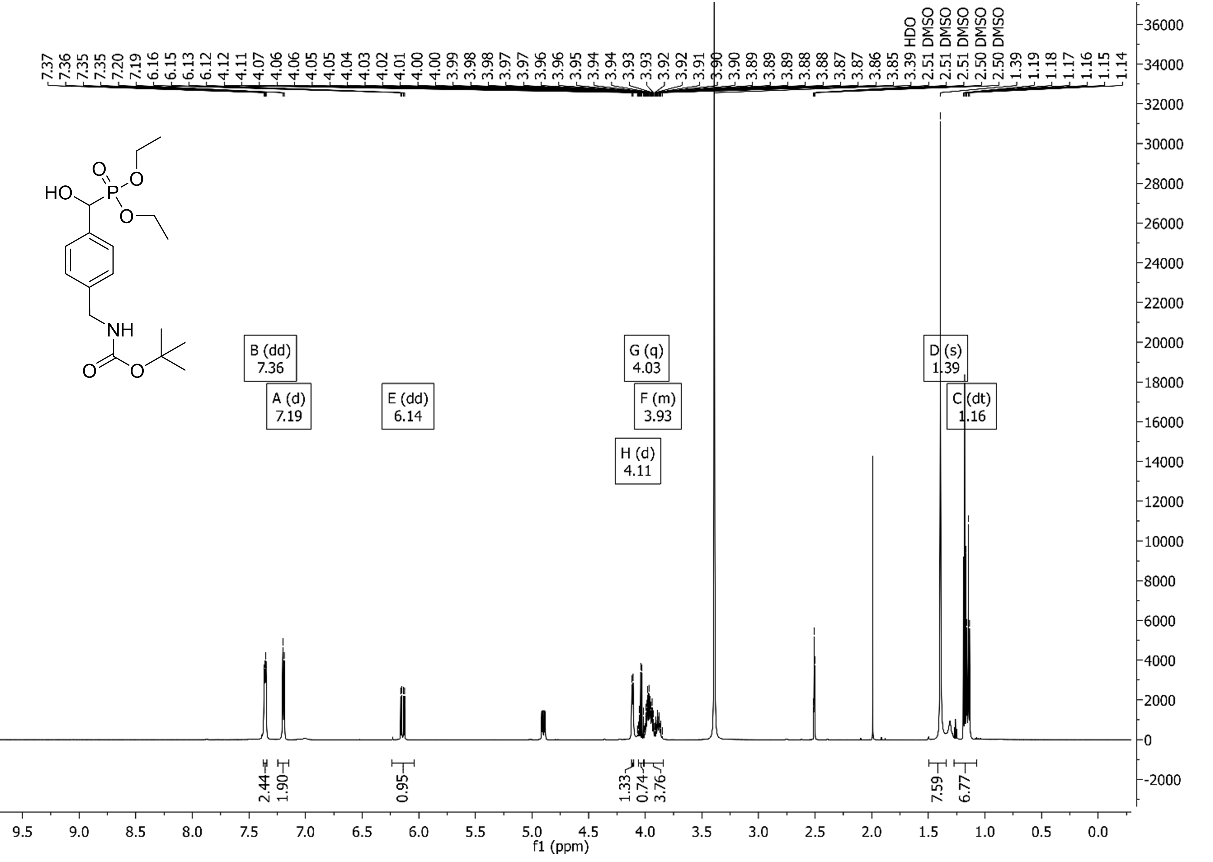


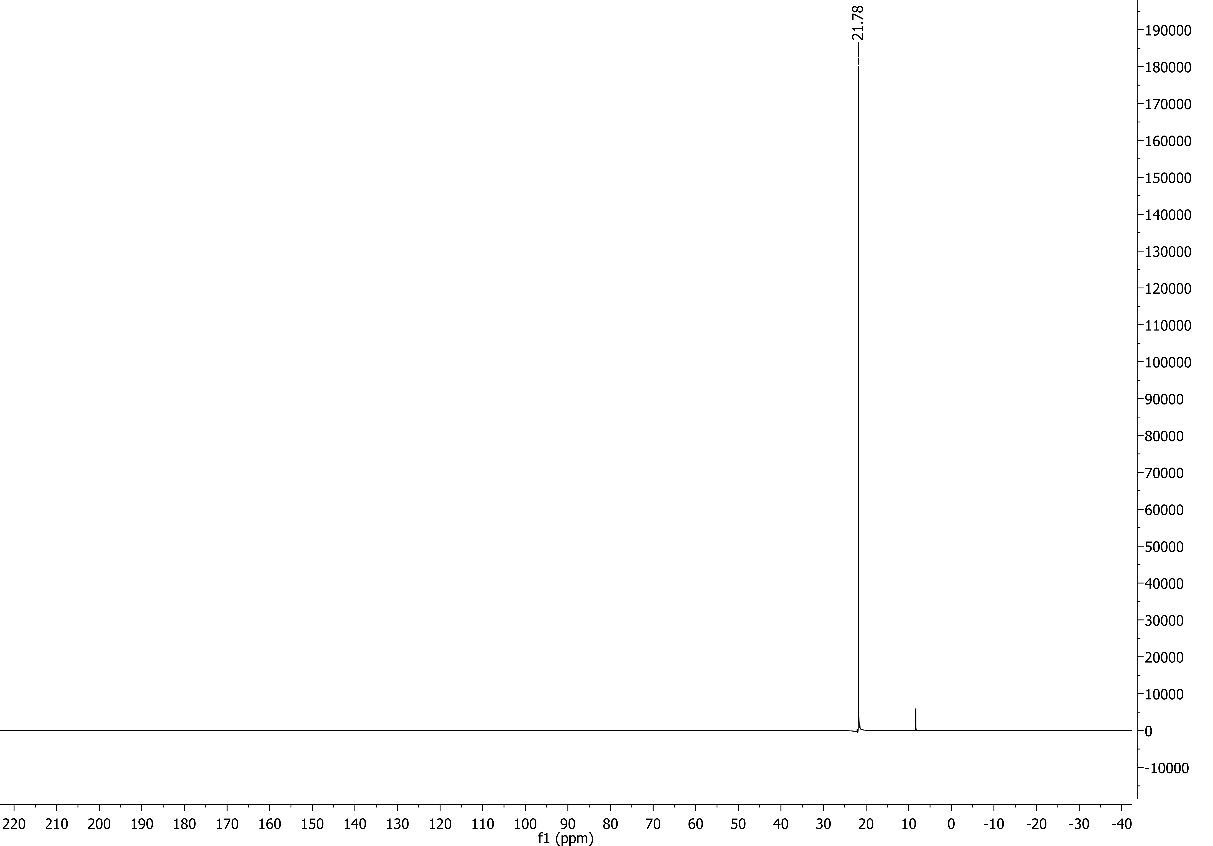


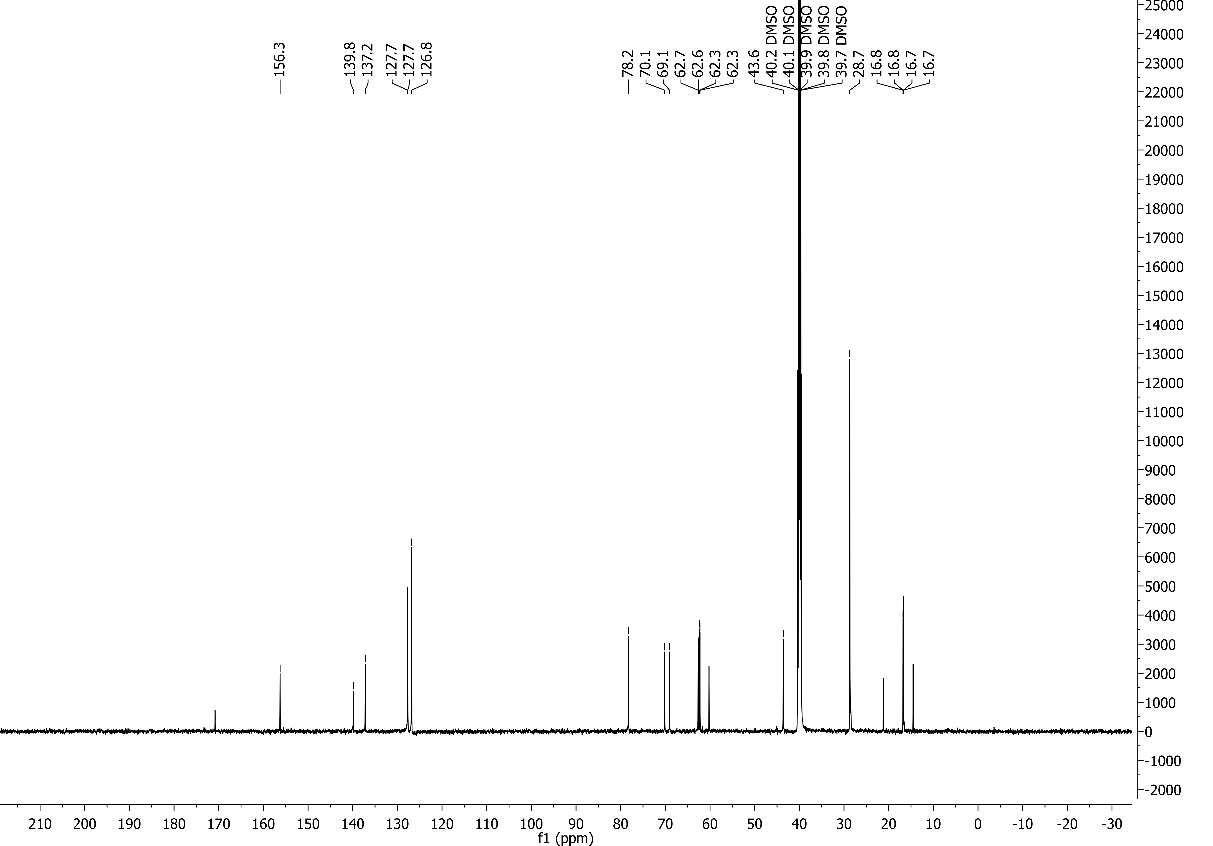


Diethyl ((4-(aminomethyl)phenyl)bromomethyl)phosphonate **(12)**


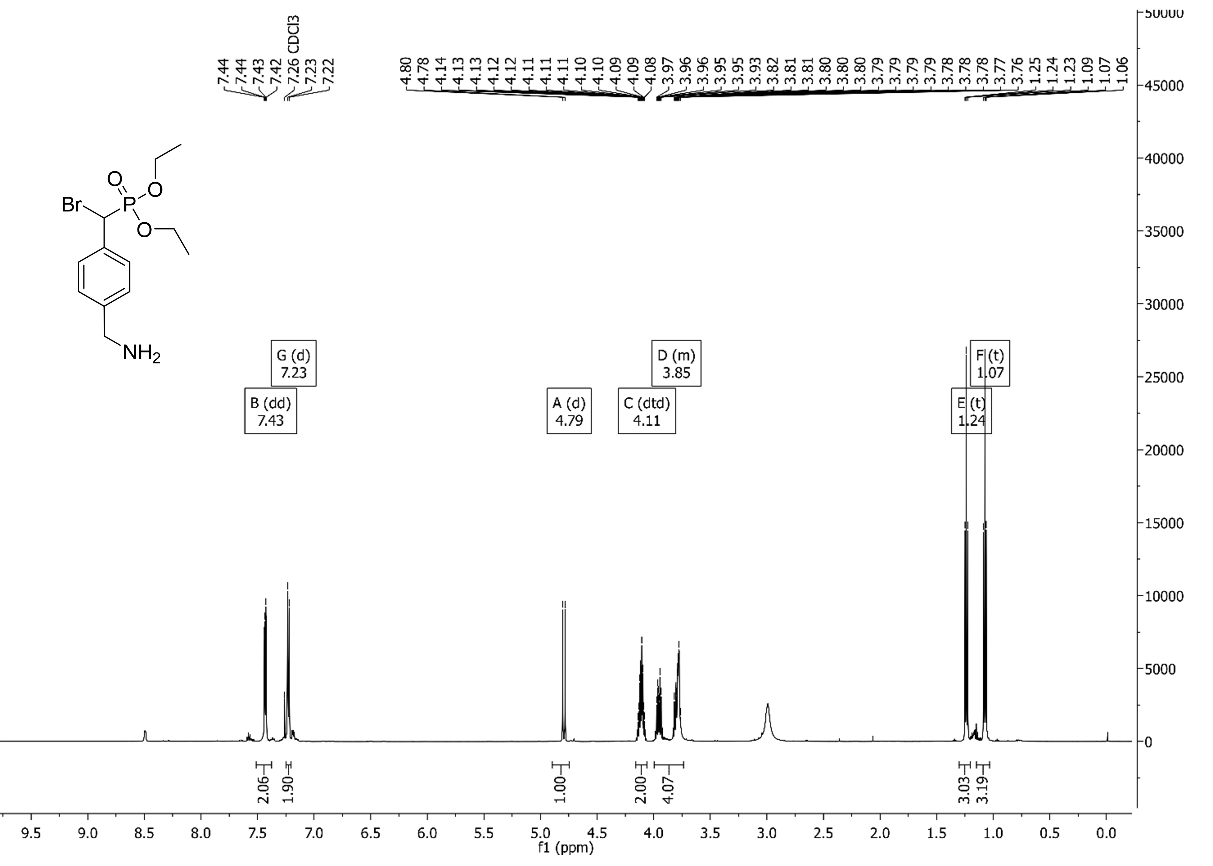


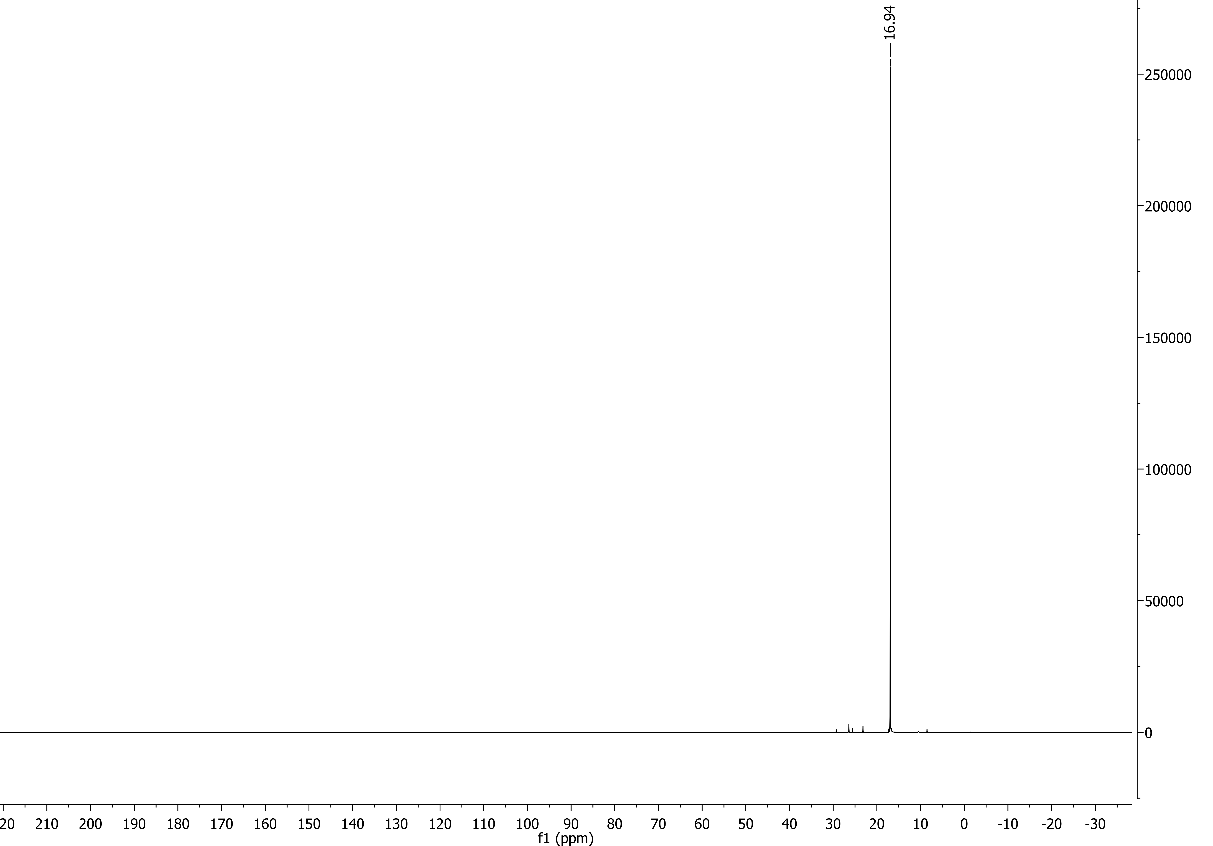


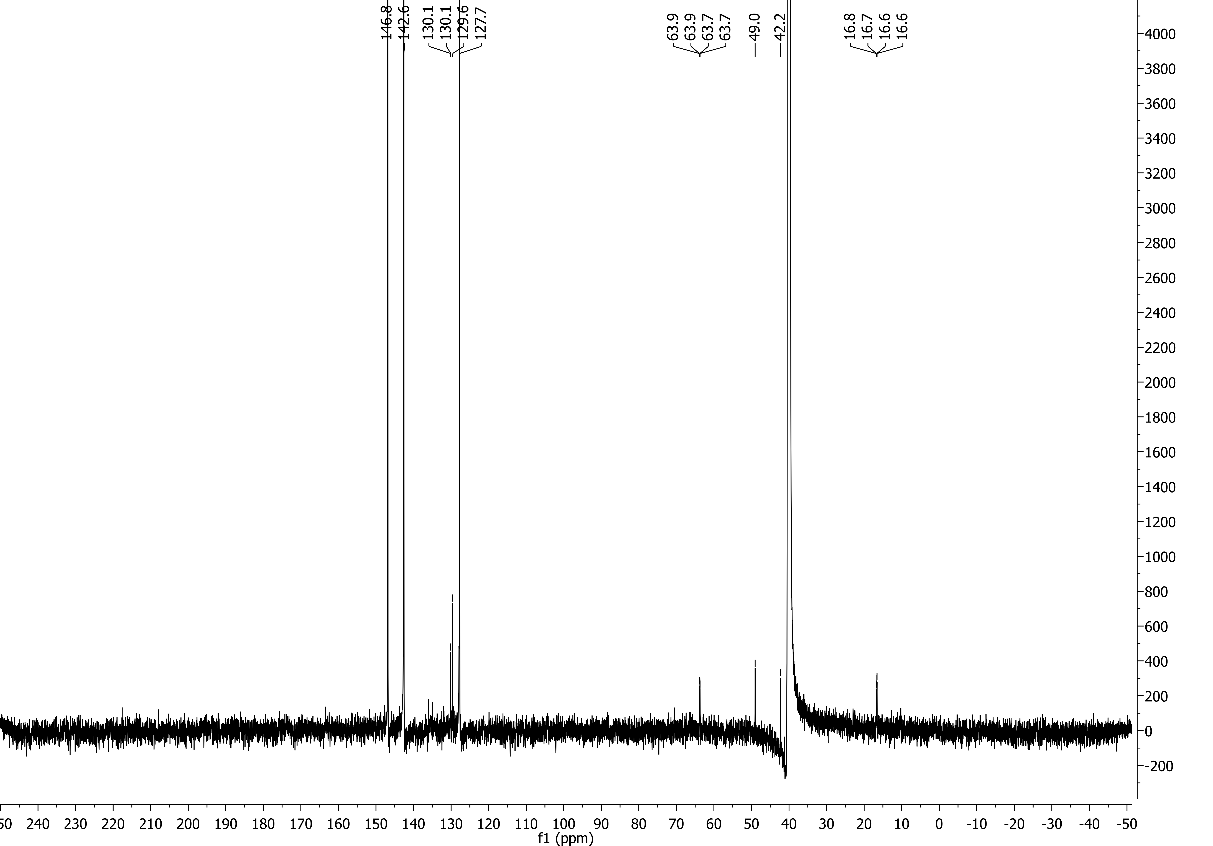


α-bromo(4-((N-5/6-TAMRA-aminomethyl)phenyl)methyl)phosphonic acid **(14)**


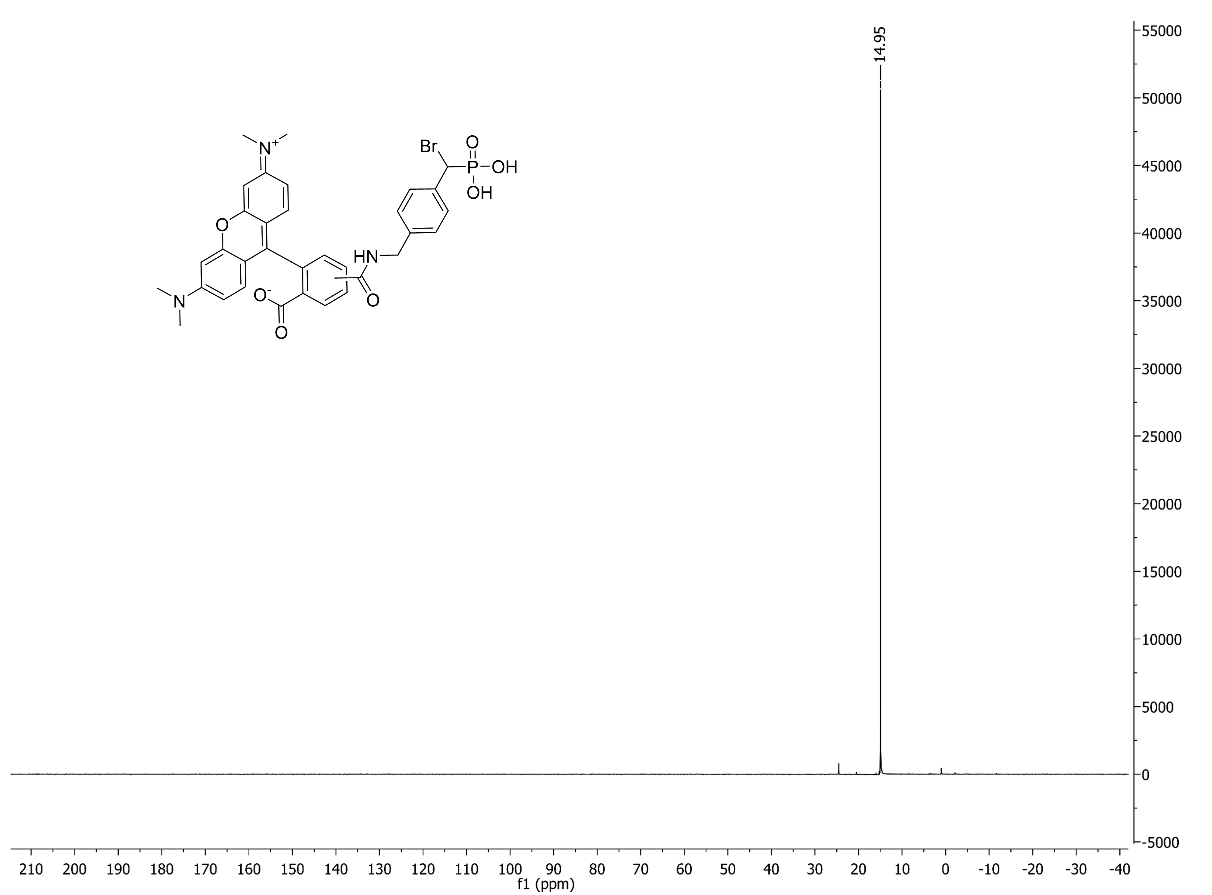

Supplement: Supplementary file 1 — Supporting File 1: The authors have cited additional references within the Supporting Information [79, 80, 81, 82, 83, 84, 85, 86, 87, 88]. [file ANIE-65-e21902-s001.docx]
